# Occurrence, Concentration, and Distribution of 35 PFASs and Their Precursors Retained in 20 Stormwater Biofilters

**DOI:** 10.1021/acs.est.4c05170

**Published:** 2024-07-30

**Authors:** Ali Beryani, Robert Furén, Heléne Österlund, Andrew Tirpak, Joseph Smith, Jay Dorsey, Ryan J. Winston, Maria Viklander, Godecke-Tobias Blecken

**Affiliations:** † Department of Civil, Environmental, and Natural Resources Engineering, Luleå University of Technology, 97187 Luleå, Sweden; ‡ NCC Sverige AB, Department of Research, and Innovation, 170 80 Solna, Sweden; § Department of Food, Agricultural, and Biological Engineering, Ohio State University, Agricultural Engineering Building, 590 Woody Hayes Dr, Columbus, Ohio 43210, United States; ∥ Department of Civil, Environmental, and Geodetic Engineering, Ohio State University, Hitchcock Hall, 2070 Neil Avenue, Columbus, Ohio 43210, United States; ⊥ Core Faculty, Sustainability Institute, Ohio State University, Smith Lab 174 W, 18th Avenue, Columbus, Ohio 43210, United States

**Keywords:** urban runoff, emerging contaminants, bioretention, filter media, fate and transport, retention, TOP assay

## Abstract

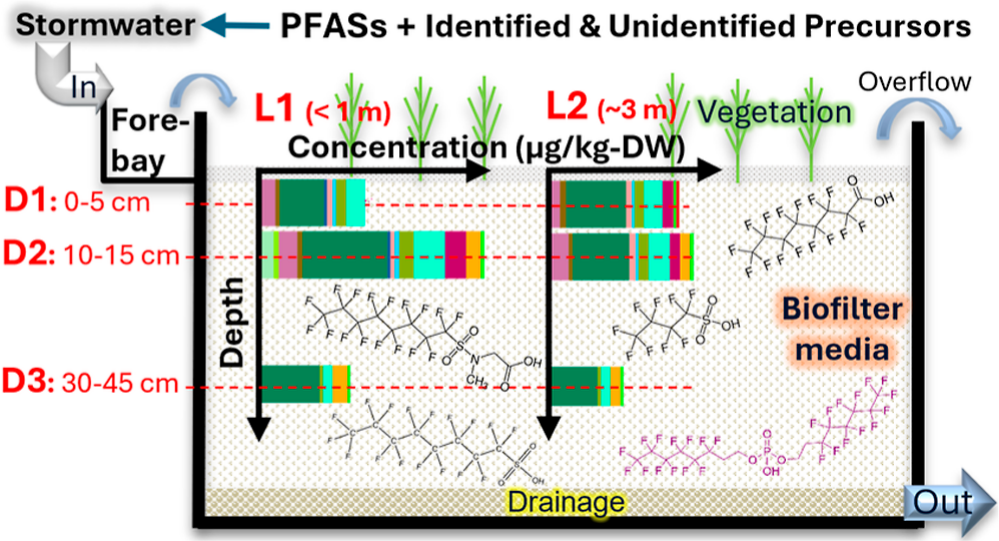

Current knowledge about the fate and transport behaviors of per- and polyfluoroalkyl substances (PFASs) in urban stormwater biofilter facilities is very limited. C5–14,16 perfluoroalkyl carboxylic acids [perfluorinated carboxylic acids (PFCAs)], C4,8,10 perfluoroalkanesulfonic acids (PFSAs), methyl-perfluorooctane sulfonamide acetic acid (MeFOSAA, a PFSA precursor), and unknown C6–8 PFCA and perfluorooctanesulfonic acid precursors were frequently found in bioretention media and forebay sediments at Σ_35_PFAS concentrations of <0.03–19 and 0.064–16 μg/kg-DW, respectively. Unknown C6–8 PFCA precursor concentrations were up to ten times higher than the corresponding PFCAs, especially at forebays and biofilters’ top layer. No significant trend could be attributed to PFAS and precursor concentrations versus depth of filter media, though PFAS concentrations were 2–3 times higher in the upper layers on average (significant difference between the upper (0–5 cm) and deepest (35–50 cm) layer). PFASs had a similar spatial concentration distribution in each filter media (no clear difference between short- and long-chain PFASs). Commercial land use and organic matter were important factors explaining the concentration variations among the biofilters and between the sampling depths, respectively. Given the comparable PFAS accumulations in deeper and superficial layers and possible increased mobility after precursor biotransformation, designing shallow-depth, nonamended sand biofilters or maintaining only the top layer may be insufficient for stormwater PFAS management.

## Abstract 2

15 PFASs and unknown C6−8 PFCA precursors were frequently found in forebay sediments and at various depths in biofilter media, often with higher concentrations in the top layers.

## Introduction

Per- and polyfluoroalkyl substances (PFASs) are synthetic organic fluorochemicals with surfactant properties and numerous industrial/commercial applications. The presence of PFASs in the environment is a major concern due to their detrimental effects on biota and human health.^1,2^ PFASs degrade slowly due to the strength and stability of carbon–fluorine bonds in their perfluoroalkyl chain and also relatively high solubility and mobility in aquatic environments due to their hydrophilic functional head.^3,4^ Until now, several states in America and the European Union have listed some PFASs (including perfluoroalkanesulfonic acids (PFSAs) such as perfluorooctanesulfonic acid (PFOS), perfluorohexanesulfonic acid (PFHxS), perfluorobutanesulfonic acid (PFBS), C8–14 perfluorinated carboxylic acids (PFCAs), and some of their salts and precursors) as bioaccumulative and environmentally persistent contaminants and have banned or proposed to restrict their use.^5,6^ Long-chain (LC) PFASs (LC-PFASs) (i.e., PFCAs ≥ C8 and PFSAs ≥ C6) are particularly concerned due to their longer half-life and higher toxicity and bioaccumulation.^7^ In contrast, short-chain (SC) PFASs (SC-PFASs), used as a replacement for the LC-PFASs, are less toxic but more soluble and mobile in aquatic environments.^8^

The limited available data indicates that urban runoff is a relevant input pathway of PFASs into the environment.^9−13^ Traffic-related materials (e.g., grease and fluids in steering, brake, and suspension systems, and road marking paints), aqueous film-forming foams (AFFFs) for firefighting, infrastructure/building materials (e.g., cables, hoses, and cement additives), house/street dust and litters (e.g., food packaging), and atmospheric deposition are some of the potential PFAS sources in urban stormwater systems.^9,11,12,14−16^ However, literature on PFAS fate and behaviors in urban environments and stormwater is still limited.^12,17^

Blue-green infrastructure, which is designed to receive and treat urban runoff on-site, may serve an important role in organic micropollutant (OMPs) accumulation/removal.^18^ Stormwater biofilters (also known as bioretentions and rain gardens) are one of the most common nature-based treatment technologies and usually consist of a filter media (often sand-based) covered by a planted topsoil layer with/without mulch.^19,20^ Water percolates vertically through the filter and is discharged through a drainage system. When inflow exceeds infiltration, water is stored temporarily in a depression on top. Several field studies have demonstrated that stormwater biofilters effectively remove many inorganic and (mainly hydrophobic) OMPs (e.g., aliphatic and aromatic hydrocarbons, phenolic compounds, phthalates, and metals).^21−26^ However, the literature on PFASs removal efficiency in stormwater biofilters is still very limited.^27^ A few column tests have shown relatively inefficient PFAS removals with sand-based filter materials,^28−31^ suggesting that their performance can be improved/restored using, e.g., biochar or black carbon amendments, addition of polymers or iron minerals, and/or controlling (un)saturation conditions.^28−33^

Fate and transport of PFASs possessing a range of hydrophobic, hydrophilic, surfactant type, and surface active properties are more complex than many better-known OMPs in soil–water–air media.^17^ For instance, polycyclic aromatic hydrocarbons (PAHs), polychlorinated biphenyls (PCBs), and petroleum hydrocarbons, the main OMPs investigated in biofilter fate studies, show high hydrophobic adsorption to organic matter (OM), suspended solids (SS), sediments, and filter material, with their fate controlled by particle transport/retention mechanisms.^22,26,34−38^ PFAS molecules and precursors, however, are involved in both hydrophobic and electrostatic adsorption to soil/sediment/SS particles and air–water interfaces (AWI), a significant PFAS retention mechanism under unsaturated conditions.^39−41^ PFASs, especially LC compounds, bind to OM through their hydrophobic head and to the media’s charged sites through their ionic functional head (either directly or indirectly via metal oxides).^42^ The electrostatic sorption/desorption interactions depend on the functional head charge (i.e., anionic, cationic, or zwitterionic), soil material (sorbent type), soil cation exchange capacity, microbial population, DOC, and changes in solution/soil pH, cations (e.g., Na^+^ and Ca^2+^), and ionic strength.^42−45^ In general, LC-PFASs are expected to be adsorbed to particles faster and thus removed by biofilters more efficiently relative to SC-PFASs.^7^ Conversely, SC-PFASs may break through the deeper layers of the filter media and therefore be more available to downstream biota and, with time, result in exposure levels similar to longer chain substances. Furthermore, PFASs that accumulate at AWI under unsaturated flow conditions^41,46,47^ (e.g., during less intense everyday rainfall events) may leach into the water phase when the media becomes saturated (e.g., during high-flow events or due to clogging of the filter material), collapsing these unstable interfaces.^29,48^ Biotransformation of the retained PFAS precursors into less sorptive terminal PFASs (e.g., SC-PFCAs, PFOA, and PFOS), which depends on oxic/anoxic conditions at the accumulation depth, is also an expected phenomenon in soil media.^15,49^ Thus, besides PFAS desorption and/or particle-bound remobilization, the transport of newly transformed PFASs may enhance their mobility and/or retention.^50^ Apart from the known perfluoroalkyl acids (PFAAs: PFCAs + PFSAs) and measured precursors, stormwater facilities may also contain many unmeasured hazardous PFAS precursors and intermediates. Total oxidizable precursors (TOP) assay provides insights into the identification and contribution of unknown precursors/intermediates.^50^ The overall stability, persistence, hydrophobicity, hydrophilicity, mobility, and potential transformation of PFAS molecules in aquatic environments make it challenging to predict their fate and transport behaviors.^51^ Thus, given the potential presence of PFASs and their precursors in stormwater runoff, assessing and understanding the retention and transport of PFASs and their precursors in filter-based stormwater treatment systems are needed from performance and management perspectives.

To date, PFAS studies on soil/sediments have mostly focused on agricultural soils impacted by contaminated wastewater treatment biosolids or surface soils near fluorochemical manufacturing facilities,^52^ along with a few on the stormwater sediments of sewers, ponds, and gully pots.^9,53−55^ However, no study has investigated PFASs and precursors in the forebay and filter material of mature stormwater biofilters in urban areas. Although some lab-scale experiments reported unpromising PFAS adsorption by conventional soil media for highly contaminated groundwater/soil,^28,56−60^ a critical review argued that PFAS sorption behaviors are more complex than can be described by a single soil/sediment property (e.g., soil organic carbon–water partitioning coefficient, K_OC_, the only parameter considered in most studies); instead, combination of several physio-chemical and kinetic factors (e.g., OM, pH, clay content, index cations, ionic strength, cocontaminants, contact time, and unsaturated/saturated flow conditions) may have significant increasing effects on PFAS sorption.^17,29,30,61^ There is particularly a critical knowledge gap regarding the fate of PFASs in biofiltration systems under field conditions, wherein the following numerous factors and their variations may also play a role: catchment land use and area, biofilter’s design characteristics such as surface hydraulic loading rate, media depth, age, initial and accumulated OM and fine particle content in the filter material, surface chemistry of soil particles, number of inlets, existence/type of forebay (pretreatment), and stormwater chemistry (e.g., TSS, pH, OM, and competing ions and OMPs).^62^ Field data on the type and concentration of PFASs and precursors accumulated in mature (around 10 years old) stormwater biofilter facilities will deepen the current knowledge about (1) the actual role of biofiltration systems in the sorption and fate of PFASs,^17^ (2) the potential remobilization and transformation of PFASs in biofilters, (3) design modifications for targeted PFAS removals in the future, (4) the long-term operation of biofilters, including maintenance/disposal needs and measures throughout their lifecycle,^26^ and (5) the health risk assessment associated with accumulated PFASs.

This study aimed to investigate the presence and long-term accumulation of 35 PFASs and their precursors (legacy and new emerging compounds) in mature urban stormwater biofilters for the first time. The study explored the PFAS distributions across various locations within these systems, including forebay sediments and biofilter materials of 20 aged sites (8–16 years old). Intra- and intersite variations/patterns for PFAS occurrences, concentrations, and distributions were also assessed using a multivariate analysis of environmental parameters, including the catchments and biofilter characteristics.

## Methods

### Field Sites

This study was carried out on 20 mature vegetated urban stormwater biofilter facilities (aged 8–16 years) located in Ohio, Michigan, and Kentucky (USA) in November 2019. Most filters had forebays and filter media depths in the range of 30–50 cm. Filters ranged from 0.1 to 20% of their respective catchment areas. To the best of our knowledge, no known PFAS emission point sources exist in the catchments. Characteristics of the biofilters, including land use type, age, catchment and biofilter surface areas, forebay presence, filter depth, media composition, and hydraulic properties are summarized in Tables S1-1 and S1-2.

### Sampling Procedure

**Figure 1:**
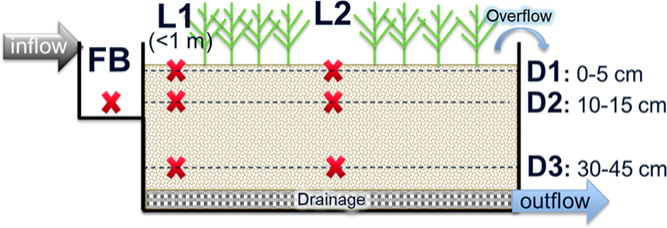
Cross-section of a biofilter facility showing the seven sampling locations: one from forebay (FB) and six from filter material at two distances (L1 and L2) from the inlet and at three depths (D1, D2, and D3).

Filter material sampling followed a method similar to that proposed by Tedoldi et al.^36^ At each site, one sample was taken from forebay sediments and six samples from the filter material at two different distances from the inlet (L1< 1 m and L2, approximately 3 m) and three depths (D1: 0–5 cm, D2: 10–15 cm, and D3: 35–50 cm; Figure 1). Overall, a total of 128 samples were collected from 20 facilities. The deepest sample’s depth (i.e., D3) varied depending on the filter media depth. For sites with multiple inlets (i.e., sites #10, #14, and #18), the sampling distances (L1 and L2) were determined based on the main inlet location (i.e., the inlet receiving the majority of the inflow, determined after a site survey examining the patterns of sediment deposition/erosion).^26^ PFAS-free equipment was utilized for sampling, including a steel spade to extract filter material cores or to scrape forebay sediments, diffusion-tight nylon plastic bags (18 × 35 cm) for sample collection, and cotton gloves/clothes. The outdoor temperature during sampling was between −12 to +6 °C. The samples were stored in a freezer (−18 °C) before laboratory analysis.

### Chemical Analysis

Samples were analyzed for PFASs by Eurofins AB, Sweden, using two techniques described in detail in Table S2: (1) targeted analysis for 35 PFASs, including C4–14,16 PFCAs, C4–13 PFSAs, three fluorotelomer sulfonic acids (FTSAs) (*n*:2 FTS; *n* = 4, 6, or 8), seven perfluoroalkane sulfonamido substances (PFASAs) (EtFOSA, EtFOSE, EtFOSAA, MeFOSA, MeFOSE, MeFOSAA, and FOSAA), perfluorooctane sulfonamide, 7H-perfluoroheptanoic acid (HPFHpA), and perfluoro-3,7-dimethyloctanoic acid (P37DMOA) using liquid chromatography-tandem mass spectrometry (UPLC-MS/MS), and (2) the TOP assay measured for 30 PFASs using the oxidation step developed by Houtz and Sedlak^63^ and UPLC-MS/MS. The method’s limit of quantification (LoQ) of substances ranged between 0.03–1 μg/kg-dry weight (DW) in 35 PFAS analysis and 0.1–2 μg/kg-DW in TOP assay (chemical characteristics and LoQ of the methods for all substances are in Table S3). Measurement uncertainties of ±23 and ±36% were reported for PFAS and TOP analysis, respectively. Additionally, water content, soil composition (to identify the total and mineral contents of gravel, sand, and fine fractions), and loss on ignition at 550 °C (LOI, to estimate OM content) were measured for all samples, specific surface area (SSA) for L1–D1 and L1–D2 samples, and pH for L1–D1 samples. Surface intake rate (SIR) was also measured at L1 and L2 to estimate infiltration rates and other hydraulic properties. The methods for each analysis are detailed in Table S2.

### Data Analysis

The increase of each PFAA (ΔPFAA) was calculated for TOP assay data to estimate the total oxidizable unknown precursors (ΣPre-PFAA). For statistical analyses, including correlation analysis (nonparametric Kendell’s tau), and tests for significance of differences (Peto & Peto modification of Wilcoxon test), the NADA-R package was used, which accounts for data including left-censored values (i.e., nondetects; concentrations below LoQ). However, substances with >80% nondetects were excluded.^64^

A multivariate redundancy analysis (RDA) was performed to explain the impact of environmental parameters on PFAS and precursor concentration variations in filter media (i.e., response variables). The environmental parameters included site age (Age), ratio of filter to catchment area (Ratio %), depth, distance location from the inlet (Loc), soil composition (i.e., percentages of gravel, mineral sand, silt, and clay), SSA, OM, pH, five land use types of commercial (Com), industrial (Ind), residential (Res), fuel station (Fuel St.), parking/road (P/Rd), SIR, and estimated saturated hydraulic conductivities (K_S_; Table S1-2). For RDA analysis, PFASs and PFASs (TOP) with >80% censored data were excluded, and nondetects of other substances were substituted with one-half of the LoQ. The RDA involved a multistep process of data transformation, significance testing of the model/parameters, checking for collinearity, and explanatory parameter selection (details in Figure S10). The final RDA ordination aimed to explain the response variables by statistically significant and technically relevant environmental variables.

## Results and Discussion

### Occurrence and Concentration of PFASs

Of the 35 targeted PFASs and precursors, 15 substances, including C5–14,16 PFCAs, C4,8,10 PFSAs, and MeFOSAA, were frequently quantified (i.e., present in >20% of all 128 samples), while 6 substances, including PFBA, PFHxS, EtFOSAA, MeFOSE, FOSAA, and PFOSA, were observed in <20% of samples of forebay sediments and filter materials. However, 14 compounds, including C5,7,9,11–13 PFSAs, three FTSAs, MeFOSA, EtFOSA, EtFOSE, HPFHpA, and P37DMOA were never quantified. PFOS and PFOA (both very persistent LC-PFASs) were the two predominant substances in the filter materials (97 and 78%, respectively) and forebays (93 and 43%, respectively), followed by PFNA and PFDA. These substances are classified as LC-PFASs with a higher hydrophobic adsorption tendency onto soil/sediments than SC-PFASs. Despite their gradual phase-out since 2016 in the United States and 2008 in the European Union,^6,65^ PFOS and PFOA are often the most recurrent PFASs (particularly PFOS) in many surface soils and stormwater sediments,^53−55,66−68^ likely due to their widespread historical use, extremely slow degradation, and production through precursor biotransformations.^63,65^ As expected, the average occurrence of LC-PFASs was higher than SC-PFASs in both forebays (34 vs 20%) and biofilter materials (44 vs 31%). Table S5 summarizes the occurrence frequency of each PFAS substance or group at different locations/depths in the biofilters.

Generally, PFASs were quantified in biofilter material more frequently than in forebay sediments (average occurrence rate of 28% vs 20% for all PFASs, 43% vs 33% for PFCAs, and 38% vs 29% for PFSAs) (Figure S1). This might be due to different functionality and maintenance regimes. Forebays capture gross pollutants and are periodically cleaned, whereas filter media are designed to accumulate both coarse and smaller pollutants over a long time. In addition, the forebay becomes filled with sediments due to inadequate design and/or maintenance,^69^ as observed in forebays studied herein that were rarely cleaned. This allows sediment to bypass the forebay and end up in the biofilter. Conversely, PFASAs had a higher occurrence rate in forebay (18%) than in biofilter media (7%). PFCA occurrences decreased in the filter material and increased in the forebay as the length of the fluorinated carbon chain increased. This could be attributed to the greater potential for particle-bound longer-chain PFCAs to settle in the forebay before they enter the biofilter.

**Figure 2:**
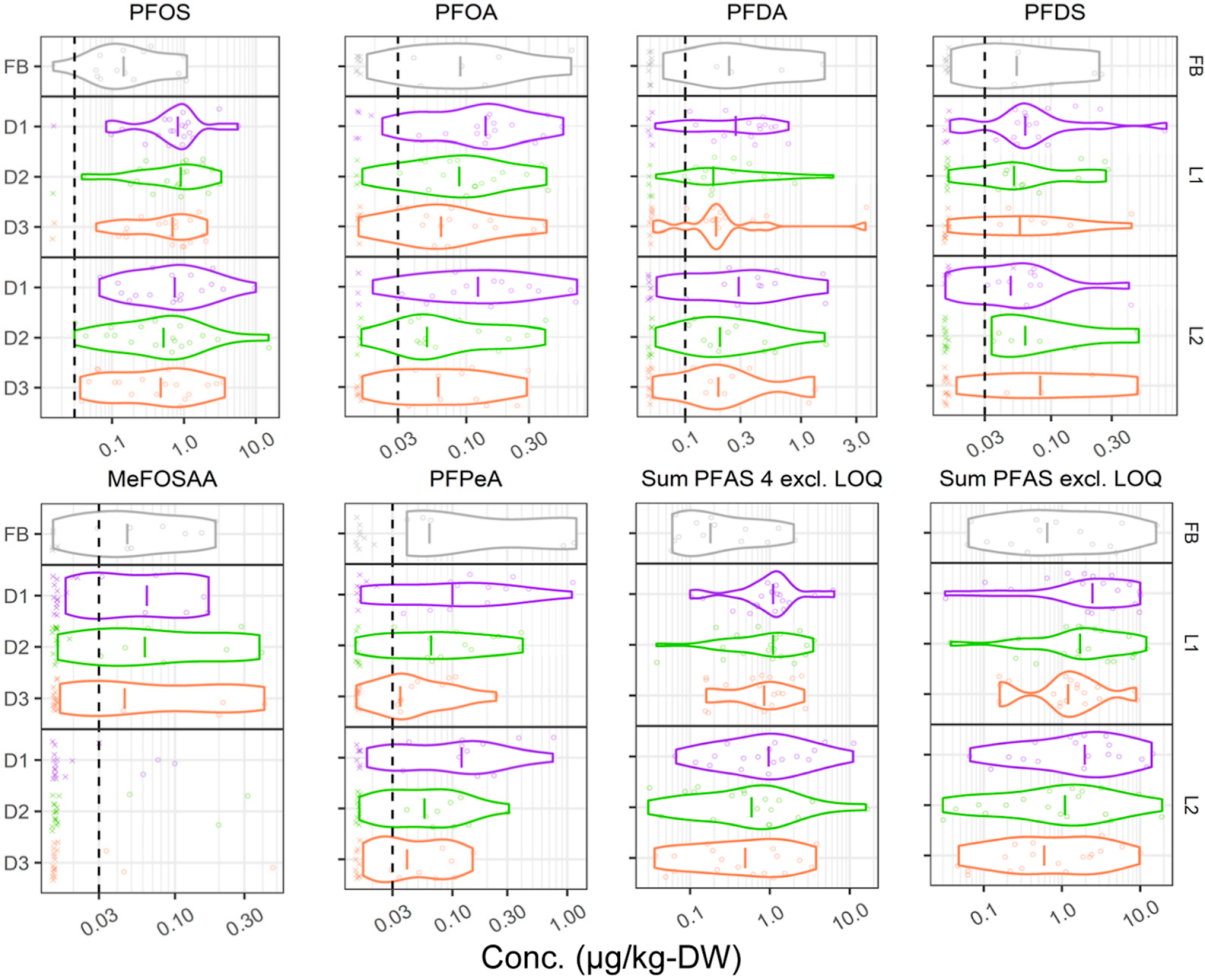
Concentration distribution of selected PFASs at forebays (FB) and biofilter materials at different depths (D1–D3) and distances from the inlet (L1 and L2). Cross and circle symbols represent censored and quantified data, respectively. Dashed lines represent the LoQ of substances, and the data below the LoQ are an approximate illustration of censored data jittered around half of the LoQ. “Sum PFAS 4” is the sum of PFOS, PFOA, PFNA, and PFHxS, and “Sum PFAS” is Σ_35_PFASs, both excluding censored data.

Figures 2 and S5 show the concentrations and distribution of the 15 most frequently (>20%) quantified PFASs in forebay sediments and biofilter media (statistical summary in Table S5). The concentration of Σ_35_PFASs (i.e., the sum of quantified PFASs at each site) ranged from <0.03 to 19 (median: 1.6) μg/kg-DW in filter media and from 0.064 to 16 (median: 0.665) μg/kg-DW in forebays. Compared to the forebays, the median concentration of all quantified PFASs in the top layer (D1) of the filter media was 5.2-fold higher for PFOS and 1.2–2.4-fold for other substances, likely because PFASs passed through the forebay freely or via finer suspended particles, coupled with the lack of maintenance in the forebays mentioned earlier. Nevertheless, Σ_35_PFASs concentration in the top layer was 1.7–2.5 times lower than that in the forebay at 4 of the 15 biofilters with a forebay. The PFAS compound with the highest concentration varied among the biofilters (Figure S6). However, generally, PFOS was the most frequently observed substance at the highest concentrations, followed by PFDoA, PFDA, and then PFUdA, PFOA, and PFTeDA. All these substances were among the LC-PFASs, which possess greater *K*_OC_ than SC-PFASs (Table S4) and therefore have a higher probability of hydrophobic adsorption onto sediment/soil under similar conditions.^7,28,42,56,70^ This could be also due to the higher surface-active properties of these LC-PFASs than SC-PFASs, causing stronger AWI adsorption under unsaturated flow conditions.^40,41^

The PFAS concentrations observed were compared to those in different urban sediments and soil media of other studies (a detailed comparison is given in Table S10). The observed concentration range of PFOS (a dominant substance in forebays; <0.03–1.1 μg/kg-DW, median: 0.15) was similar to that in sediment from urban stormwater ponds and gully pots in Sweden,^53,54^ approximately half of the urban (mixed land uses) stormwater pond sediments in Minneapolis-St. Paul, USA,^55^ and 1 to 12-fold lower than the surficial sediments of the five Great Lakes, USA,^71,72^ but 2 orders of magnitude lower than urban runoff particles/debris in highly trafficked and industrial areas in Minneapolis, USA.^9^ Compared with PFOS and PFOA concentrations found in urban road sweeping materials in Gainesville, USA,^15^ lower ranges were found in the forebays studied herein, although much higher levels have been reported in road dust within a 3 km radius of PFAS-related manufacturers in China.^73^

PFOS concentrations observed in the biofilter materials, ranging from <0.03 to 15 μg/kg-DW (median: 0.7), were higher than the background PFOS levels in uncontaminated surface soils (0–15 cm) of other studies.^68,73−75^ Further, they were higher than the estimated global PFOS median concentration of 0.47 μg/kg-DW.^68^ As shown in Table S10, comparable PFOS concentrations to the filter materials herein have been reported in surface soils of semi-industrial and mixed urban areas in North America, Europe, Korea, and some rural areas in China.^52,67,76,77^ However, the observed PFOS concentrations were often 10-fold lower than values found in surface soils of dense residential-industrial areas in China,^78^ and several orders of magnitude below the levels found in highly contaminated surface soils (which could reach hundreds of μg/kg-DW) near PFAS-related manufacturers, firefighting training facilities, and military/airport areas of different studies due to the release of AFFFs, atmospheric deposition, and/or groundwater wells recharged by PFAS-polluted surface waters.^73,79−82^

Compared to health-based PFAS guidelines for soils, the observed concentrations were far below the residential soil screening level of 6000 and 16,000 μg/kg-DW for PFOS and PFOA, respectively, as proposed by the US EPA,^83^ and did not exceed the Minnesota Pollution Control Agency’s soil reference values for recreational (2600 and 2500 μg/kg-DW for PFOS and PFOA, respectively), industrial (14,000 and 13,000 μg/kg-DW), and residential (2100 μg/kg-DW for both) land uses.^84^ However, PFOS concentrations in the filter material at 15% (*n* = 3) of the sites (especially in the upper layer) exceeded the preliminary Swedish guideline value of 3 μg/kg-DW for sensitive land uses (e.g., residential and schools). Thus, these materials could be classified as hazardous waste; however, they remained below the Swedish guideline value of 20 μg/kg-DW for less-sensitive land uses (e.g., industrial uses).^85^

### Occurrence and Concentration of “Known” and “Unknown” PFAS Precursors

The most frequently found “known” precursors in the forebays and biofilter media were three PFASAs, including MeFOSAA (57 and 23%, respectively), EtFOSAA (29 and 10%, respectively), and MeFOSE (14 and 8%, respectively). FOSAA and PFOSA were also present but only occasionally quantified (<7% in both biofilter media and forebay samples). On average, these “known” precursors contributed to 12.2 ± 22.4% and 2.6 ± 6.1% of Σ_35_PFAS concentration in the forebays and filter materials, respectively. These compounds have the potential to biologically transform into intermediate and/or terminal PFASs (i.e., PFOA and more likely PFOS), indirectly contributing to a long-term source of PFAAs of high concern in the biofilter media and potentially to treated biofilter effluent.^82,86^ As evidences, in 85% of the sites where PFASAs were quantified, an increased level of PFOS concentration was measured as one of the terminal substances after TOP assay. MeFOSAA and EtFOSAA can also be byproducts of MeFOSE and EtFOSE biotransformation, respectively. These compounds are used in textiles, paper, and packaging products and are often present in dust and water/sediment environments.^87,88^ However, none of the measured PFASAs were quantified in the oxidized samples, likely because of significantly stronger chemical oxidation (compared to more modest biological oxidation)^63^ of PFASAs into PFAAs during TOP assay and/or their higher LoQs in TOP assay compared to targeted PFAS analysis. FTSAs, well-known PFCA precursors used in AFFFs and textile, metal, plastic, and electronic industries,^14^ were not found in biofilter samples herein, although these compounds (especially 6:2 FTS) have been occasionally detected by other studies of stormwater/waterbody sediments or urban surface soils (also see Table S10).^15,54,63,67,89,90^ This could be due to FTSA biotransformation, low FTSA levels in stormwater, and/or lower concentrations expected in filter media (mixture of sediments and filter material) compared with stormwater sediments alone.

**Figure 3:**
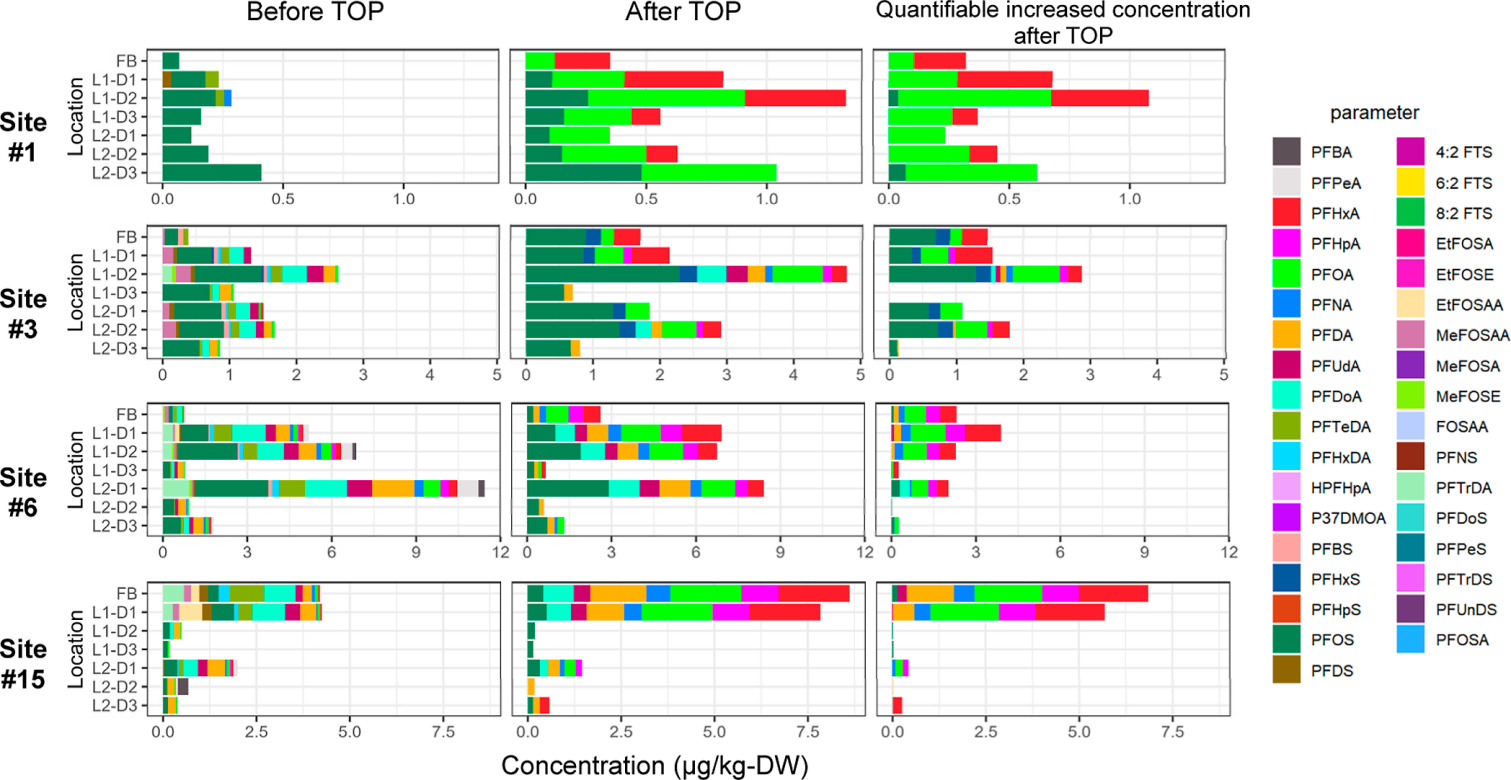
Comparison of PFAS concentrations before and after the TOP assay and the quantifiable increased concentrations after TOP assay at some selected facilities to identify the contribution of unknown precursors.

C6–12 PFCAs and PFOS were the frequently detected terminal compounds (>20%) after oxidizing the forebay and filter material samples, while C4,5,14 PFCAs and C4,6,10 PFSAs were also occasionally found (<20%) (Figures S2 and S4; Table S6). Like for the PFAS analysis, PFOS and PFOA were the most recurrently found substances after oxidation. The total concentration of 30 PFASs after TOP assay (Σ_30_PFAS_TOP_) ranged from 0.1 to 21.9 μg/kg-DW (median of 1.4 and 2 for forebay sediments and filter materials, respectively), which was higher than Σ_35_PFAS. At 18 biofilters, a comparison of C6–10 PFCAs/PFOS before and after the TOP assay (Figures 3 and S6) revealed increased concentrations after oxidation. The increase in each PFAA concentration (ΔPFAA) and its ratio (ΔPFAA/PFAA), which can be an indicator of the level of total oxidizable precursors for a given PFAA, are shown in Figure S6 for each site.

One should note that the higher LoQ (i.e., lower sensitivity) of the TOP assay than in the targeted PFAS analysis may lead to underestimation of unknown precursors; thus, Σ_30_PFAS_TOP_ had a similar or smaller range and/or median than Σ_35_PFAS at certain locations of some facilities, especially in deeper layers where the occurrences/concentrations were generally lower (Figure S6). Notably, the rise in C6–8 PFCA concentrations was particularly high, with a mean increase of 1616% in forebays and 654% in biofilter media across all sites (Table S8). Additionally, C9 PFCA showed a notable mean increase of 896% in forebays among the six sites (Table S8). PFOS, however, had a mean increase of 200% in six forebays and only 24% in the filter media of all sites. Regarding PFSAs, the increase was exceptionally high at site #3, where PFHxS and PFOS levels rose by 1010 and 345% in forebay and averaged 905 and 81% in filter media, respectively. Generally, analysis of ΔPFCAs confirmed the accumulation of numerous unidentified precursors (predominantly C6–8 PFCA precursors) in addition to those included in the targeted PFAS analysis. Transformation of unmeasured PFCA precursors, such as FTCAs and FTOHs (also found in stormwater and surface soils),^52,91^ fluorotelomer-based polymers, and polyfluoroalkyl amides might be important contributors to the elevated C6–8 PFCA concentrations.^52,92−94^

Unlike other sites, forebay sediments at sites #11 and #14 contained extremely higher unknown oxidizable PFCAs (ΔPFCAs) compared with the filter media. This could be attributed to specific sources of precursor(s) in the form of coarse pollutants trapped in those forebays.

### Distribution Patterns of PFASs and Their Precursors in Biofilter Media

The mean occurrence rate often decreased with increased depth in the filter media (Figure S3): 30 to 16% for all PFASs, 56 to 29% for PFCAs, 45 to 29% for PFSAs, and 10 to 5% for PFASAs, respectively, for D1 versus D3. However, the opposite trend (i.e., increasing occurrence with depth) was observed for MeFOSE, EtFOSAA, PFPeA, PFHxA, and PFHxS. These confounding results were likely associated with the fate/transport characteristics of different PFAS groups; for example, PFPeA and PFHxA (both among SC-PFASs having higher mobility than LC-PFASs) were expected to be transported to deeper layers, while MeFOSE, MeFOSAA, and EtFOSAA accumulated in the upper layers can be transformed via volatilization at warm temperatures, aerobic biodegradation into more stable PFASs (mainly PFOS), and/or possibly taken up by plant roots.^45−47^ At a given depth, there was no significant difference between the PFAS occurrence rates at the two distances from the inlet (i.e., L1 and L2), suggesting relatively little spatial variability in PFAS within biofilters (Figure 2).

With increased depth, PFAS concentrations varied randomly among the biofilters without any discernible trend (Figure S7). Nevertheless, on average across all sites, the concentration of Σ_35_PFASs in the top layer (D1: 0–5 cm) (median: 2.1 μg/kg-DW) was approximately 2.0 ± 1.5 and 3.0 ± 2.7 times higher than that in the middle (D2: 5–15 cm) and the deepest layers (D3: 35–50 cm), respectively. This concentration difference was statistically significant between D1 and D3 (pairwise Wilcoxon *p*-value < 0.05; Table S7 and Figure S8) for Σ_35_PFASs and many of the frequently quantified substances among all biofilters, except for MeFOSAA (likely due to limited quantified data points) and PFOS (potentially due to background levels in the blended filter material prior to operation of facilities, breakthrough of abundant PFOS in the biofilters over time, and/or site characteristics variability as discussed later). However, there was no significant difference (pairwise Wilcoxon; Table S7) between the concentrations when moving from D1 to D2 and from D2 to D3 for most PFASs. In many biofilters but specifically for sites #3, #6, and #7 with relatively higher loading rates, i.e., smaller FA/CA ratios (Table S1), D2 had higher concentrations than D1 for most quantified PFASs, likely due to more frequent saturated flow conditions and greater ponding hydraulic head which result in decreasing contact time, removing/collapsing the AWIs, preventing air–water interfacial accumulation, and/or potentially remobilizing AWI-retained PFAS fractions from the superficial layer.^29,30,47^ Although the highest PFAS concentrations were often found in the upper layers, results indicate that PFASs penetrated deeper into biofilter media, aligning with results from sand column studies on PFAS transport/removals.^28,56^ This finding contrasted with the retention patterns documented for other more hydrophobic OMPs, such as PAHs and PCBs, as well as metals and microplastics, which mostly accumulate in the upper 5 cm of biofilters.^26,36,37,95−97^ In eight biofilters, greater PFAS concentrations were observed closer to the inlet (L1) compared with L2 (Figures S6 and S7). However, similar to a study by Furén et al.^26^ on other OMPs accumulated in biofilter facilities in Ohio, Michigan, and Kentucky, the distance from the inlet (L1 vs L2) was generally not a significant factor in the accumulation of most PFASs (except for PFDS and PFTeDA, which demonstrated a significant decreasing trend along the flow path probably due to their higher hydrophobicity). This suggests an overall reduced dependency of PFASs on the retention of sediments and their transport by stormwater spreading over the biofilters. Furthermore, unlike depth profiles obtained in sand-based biofilter columns for synthetic stormwater treatment,^29^ no clear distinction in concentration reduction from D1 to D3 was observed between SC- and LC-PFCAs (Figure S6), likely due to low occurrences and concentrations of SC-PFCAs (i.e., more nondetects and higher analytical uncertainties), variability in D3 sampling depth based on the filter beds’ depth, and/or generally less effective adsorptions on sand (compared to amended sand with biochar or black carbon). A clearer difference between concentrations in surface and deep samples might be achieved in a theoretically deeper biofilter, as deeper profile results from surface soils have shown.^47^ Nonetheless, a greater reduction in concentration with depth (D1 vs D3) was noted for certain longer-chain PFCAs (C12 and C14) compared to the shorter LC-PFCAs (C8–C11), which indicated their greater adsorption tendency onto particles, filter media (Table S4), and the AWIs.

C6,8 ΔPFCAs and ΔPFOS in D1 showed significantly higher occurrences and concentrations (pairwise Wilcoxon *p*-value <0.05) compared to D3 across all sites (Table S7), which was consistent with the literature in depth profiles of some precursors in biofilter columns and contaminated soils.^29,47^ At half of the sites, the sum of C6,8 ΔPFCAs and ΔPFOS concentrations in D1 was 10.7 ± 14.6 and 16.1 ± 19.3 times higher than that in D2 and D3, respectively. Neutral, cationic, and zwitterionic precursors (e.g., fluorotelomer alcohols/acrylates and per-/polyfluoroalkyl amides/sulfonamides/ammonium salts) bind more strongly to negatively charged soil particles and AWIs than PFAAs (Table S4) and can biologically transform into less sorptive anionic PFAAs such as those observed in our study.^29,49,82,89,94^ The greater accumulation of PFAA precursors in D1 and D2 (0–15 cm) may pose a heightened concern, as their biotransformation in these aerobic layers occurs at significantly faster rates than the anaerobic zones of deeper layers.^49^ As such, their mobility in the porous media and, thus, their leaching from the biofilter media into the stormwater may be enhanced. This suggests a potential long-term, indirect source of the more mobile PFAAs that may contribute to downstream contamination. However, their bioavailability to the biofilter bacteria and their biodegradation rates remain unclear in this study.

### Impact of Environmental Variables

The final RDA model showed that 11 out of 18 environmental parameters analyzed, i.e., sampling depth, site age, FA/CA ratio (ratio %), percent sand, silt, clay, and OM, and three land uses (Com, Ind, and Fuel st.), significantly explained 45.1% of the variance in PFAS concentrations in biofilter materials across sites and sampling locations (*R*^2^ = 0.45, *p* = 0.001, which generally indicated a moderate regression). Yet, the first two statistically significant RDA components (RDA1 and RDA2) accounted for 40.9% of the variance (adjusted *R*^2^ = 0.4; *p*_RDA1_ = 0.001, *p*_RDA2_ = 0.024). The contributions of each canonical axis (RDA component) and each explanatory variable to the correlations as well as their statistical significance level are reported in Table S11.

**Figure 4:**
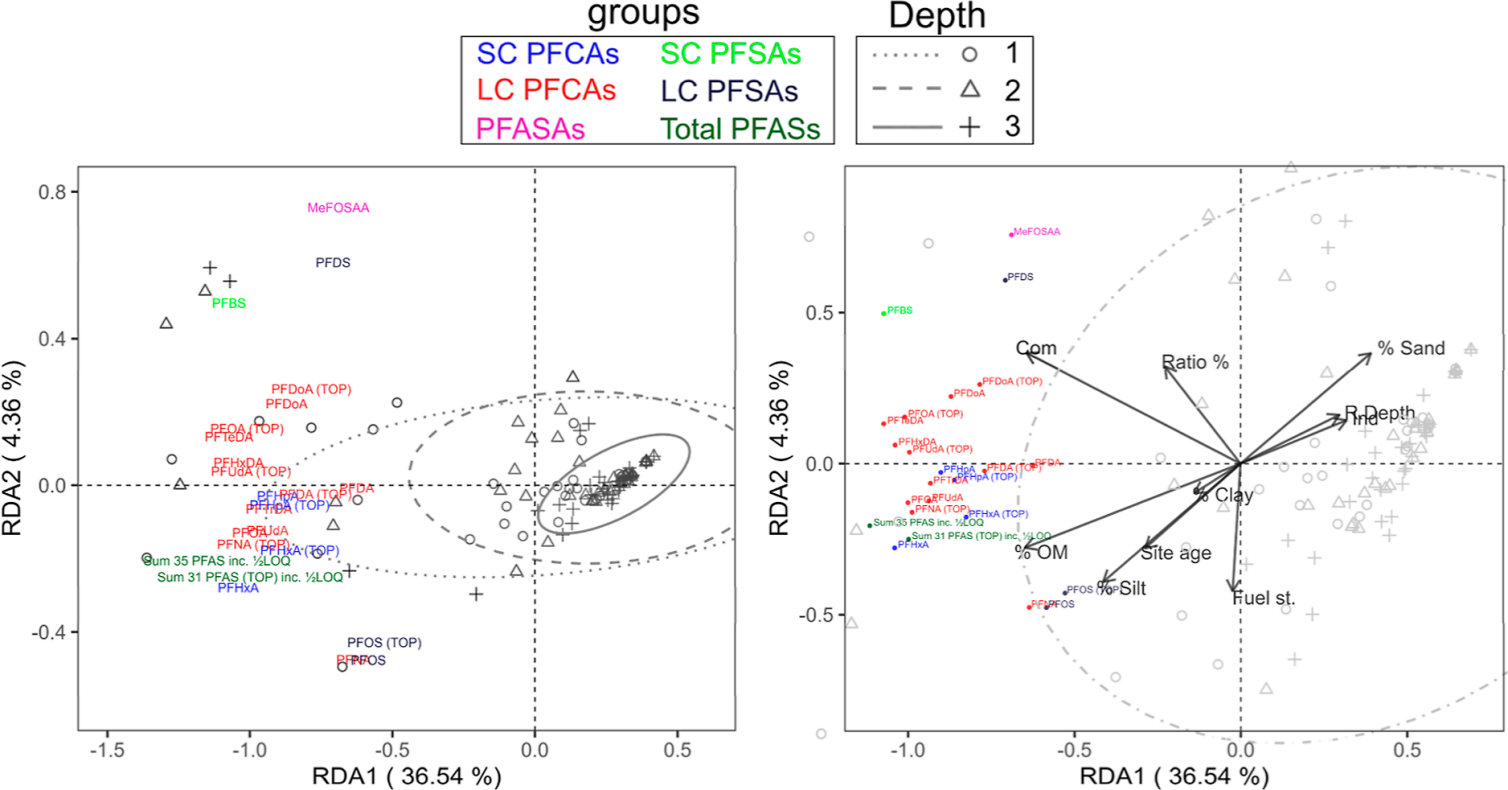
Type I (left) and type II (right) scaling plots as RDA1 and RDA2, the two most important components of the RDA model. The type I plot illustrates the similarities in the response variables (PFAS concentrations), and type II, the effect and correlation of explanatory (environmental) variables in explaining the response variations. The scores of site observations for biofilter materials at three different sampling depths are shown by symbols and categorized by their corresponding 95% confidence ellipses; the scores of response variables at six different classes are shown by colored names; the effect of explanatory variables is shown by arrows from the centroid.

Concentration variation decreased with an increased depth in the filter media (type 1 RDA plot in Figure 4). PFAS concentrations at D1 varied the most among different substances, while the clustering of samples collected at D3 indicated similar PFAS concentrations due to their lower concentrations closer to LoQ and possibly similar background concentrations in the blended filter material prior to the operation of facilities. Many of the frequently quantified PFASs were clustered together, but some (i.e., MeFOSAA, PFDS, PFBS, and to some extent PFOS and PFNA) stood out from the middle cluster along the weaker RDA2 component, meaning that their concentrations were less similar to the other substances across sites. Kendall’s Tau correlation test between these and other substances corroborated this finding (Figure S9). This suggested that these substances might behave differently in terms of their source, level/occurrence (availability) in runoff, and/or fate and transport behaviors. The difference observed for PFOS was likely influenced by its high availability, while for PFBS, PFDS, and MeFOSAA, it was influenced by their low availability. Transformation of precursors, such as MeFOSAA into PFOS, might also contribute to the observed variances for both substances. In the type I plot (Figure 4), most PFASs were typically closely paired with their corresponding PFAS(TOP) concentrations (if quantified), indicating an overall similarity between their concentrations across biofilters and sampling locations before and after TOP assay. However, PFHxA, PFOA, and PFNA demonstrated relatively greater distance, suggesting a significant accumulation of their known/unknown precursors and their potential oxidation as an additional source.

Com and percent OM, and silt more strongly affect the variation in the concentration matrix than the other parameters (Figure 4 type II RDA plot alongside the correlation heat map in Figure S9). These parameters (and to a lesser extent the site age and percent clay) were positively correlated with accumulated PFAS and precursor concentrations. Depth and Ind had an opposite effect (negative correlations), and ratio % and Fuel st. had weak or no effect on concentration variations. The stronger correlation of Com with MeFOSAA, PFDS, PFBS, and PFCA precursors may suggest that commercial land use was likely a source of these substances, but further research is needed to confirm their prevalence in commercial areas. Generally, the RDA confirms that PFAS concentrations were negatively associated with depth but not with distance from the inlet. Additionally, the correlation for LC substances was not significantly different from that for SC substances.

As expected, the percent OM, which was negatively associated with depth, was positively correlated with PFAS concentrations, particularly for longer-chain PFCAs and PFOS (Figure S9). This could be attributed to the greater capacity of the filter materials with higher OM to adsorb PFASs from their hydrophobic heads, especially at the biofilter surface where the mulch layer contributes to OM formation. While the OM content of soil is recognized as a significant parameter controlling the adsorption of LC-PFASs, it is not always the only indicator of PFAS sorption to soil/sediments.^39,43,70^ A weak or nonsignificant correlation of OM was observed, for instance, for MeFOSAA and PFCA precursors, which is supported by findings from previous studies.^89^ Other factors, such as low fine particle content and an increase of cation content (and ionic strength) in filter material, can diminish the adsorption capacity of a biofilter over time.^39,70^

While all studied land uses included potential sources of PFASs in stormwater, which added to the accumulation of PFAS in biofilters, land use did not demonstrate a clear relation with observed concentrations, again aligning with findings of a similar study on other OMPs by Furén et al.^26^ However, commercial areas had a moderate influence on the concentration variations, showing a significant positive correlation. Xiao et al.^9^ also found commercial areas to be more impactful contributors of PFAAs in stormwater runoff compared to residential areas. However, the limited number of sites across different land uses and different filter designs and catchment characteristics, even within one land use, makes it difficult to discern potential impacts.

Although SIR and estimated K_S_ were not significant in the RDA model, SIR showed significant moderate to weak correlations with some individual compounds (C6,8–14 PFAAs and PFDS; Figure S8), indicating that increased infiltration rates (i.e., decreased contact time) likely negatively impacted long-term PFAS retention due to their kinetically limited sorption in such fast-flow practices (Tables S1-1 and S1-2), as previously revealed by other studies.^29,30^

Unexpectedly, SSA was often negatively correlated with PFAS concentrations (Figure S9), likely due to the generally low PFAS concentrations and/or cocontamination effects (e.g., DOC and other OMPs competing for sorption sites)^30,45^ making SSA irrelevant for sorption sites on soil particles. The limited number of SSA measurements may have also influenced the correlations, as it was a nonsignificant parameter in the primary RDA model, as well.

While it was initially assumed that site age would be a significant factor for PFAS accumulations in biofilters, site age was often not statistically significant or only weakly correlated with PFAS concentrations. This could be attributed to the limited age variation among the studied sites (8–16 years; mean ± SD: 10.8 ± 2.2, i.e., lacking both newly built facilities and sites having been in operation for several decades). Including a larger age range of such sites may have provided a clearer understanding of the impact of age on PFAS accumulations.^26^

While we observed some trends in the RDA model, the limited number of sites compared to the numerous explanatory factors of urban stormwater quality and biofilter performance limits the identification of relevant factors and their trends in explaining PFAS accumulations. Studies with a higher number of sites and replicates of each biofilter type could enhance the model results.

## Environmental Implications

PFOS and LC-PFCAs were consistently dominant in both occurrence and concentration across all biofilters. On average among biofilters, the concentration of Σ_4_PFAS (sum of PFOA, PFNA, PFOS, and PFHxS) and Σ_11_PFAS (sum of PFBS, PFHxS, PFOS, 6:2 FTS, PFBA, PFPeA, PFHxA, PFHpA, PFOA, PFNA, and PFDA), the PFAS groups sorted/prioritized by European/American regulations,^5,98,99^ represented 58 ± 29% and 74 ± 25% of the total concentration of 35 PFASs, respectively. The remainder was attributed to known PFAS precursors, among which PFASAs (especially MeFOSAA) were dominant. Thus, while only analyzing Σ_4_PFAS is likely insufficient, targeting Σ_11_PFAS may in general provide a representative measure of the current contamination level in biofilter facilities. However, Σ_11_PFAS may not fully present the accumulated concentration in the most polluted locations (forebay and L1-D1 in filter material), accounting for only 55 ± 28% of Σ_35_PFASs, because of the higher probability of precursor accumulation in those locations. While incorporating certain PFAS precursors in the targeted PFAS analysis is advantageous, conducting a TOP assay may offer a more comprehensive view of the total PFAS contamination level, given that concentrations of unknown PFAA precursors were found to be up to ten times higher than PFAAs herein.

Analysis of total known/unknown oxidizable precursors suggested that the high occurrence and concentration of PFOS and LC-PFCAs, despite phasing them out from production, could be also a result of gradual biodegradation of their precursors (especially C6–8 PFCA precursors) in biofilters. Furthermore, precursor transformations into these more mobile terminal compounds, which are expected to occur in the surficial media layers at a faster rate, may enhance the leaching and downward transport of PFASs, thus potentially posing an additional risk to receiving waters.

Regarding biofilter operation and maintenance, prior research on micropollutants such as heavy metals, microplastics, PAHs, and PCBs, as well as on clogging, suggests that periodically removing only the top layer of biofilter media may suffice to prevent saturation and restore filter functionality.^26,36,37,97^ However, although the upper 5 cm often contained the highest concentrations of PFASs and unknown precursors, they migrated into deeper layers considerably. Additionally, considering precursor biotransformation and consequently increased mobility, concentrations in lower layers may increase over time. Thus, managing only the top media layer and trapped sediments on the surface may not suffice for more complex and mobile contaminants like PFASs. Still, prioritizing the maintenance of the superficial layer remains crucial for various accumulated pollutants. For the same reason, designing shallow-depth biofilter media will likely not be sufficient for effective removal of PFAS and associated precursors from stormwater, and amendments for increased adsorption capacity may be required.

As for reuse/disposal purposes, while observed PFAS concentrations did not reach the hazardous waste thresholds according to current primary soil/sediment regulations for PFASs in the USA, PFOS concentrations occasionally exceeded Swedish guideline values for soils in areas with sensitive land use and consistently surpassed the EQS for freshwater sediments. Nonetheless, the potential risk of higher PFAS concentrations in older biofilters should be evaluated under other climatic conditions, biofilter ages, and catchment land uses to further evaluate predictive factors further.

## Data Availability

All data are available at 10.5878/hm7t-xs34.

## Supporting Information Available

Additional details of site and PFAS characteristics, analytical methods, statistical/spatial analyses of occurrence, concentration, and distribution of PFASs before and after TOP assay, comparisons with other studies, and RDA modeling process and its final summary results (PDF)
