# Supplementary material for: Occurrence, Concentration, and Distribution of 35 PFASs and Their Precursors Retained in 20 Stormwater Biofilters
Source: Environ Sci Technol. 2024 Jul 30;58(32):14518–29. doi: 10.1021/acs.est.4c05170 (PMC11325539; doi:10.1021/acs.est.4c05170)
Supplement: Supplementary file 1 — es4c05170_si_001.pdf [file es4c05170_si_001.pdf]

Supporting Information

Occurrence, concentration, and distribution of 35 PFASs and their precursors retained in 20 stormwater biofilters

Ali Beryani [a,*], Robert Furén [a,b], Heléne Österlund [a], Andrew Tirpak [c], Joseph Smith [c], Jay Dorsey [c], Ryan J. Winston [c,d,e], Maria Viklander [a], Godecke-Tobias Blecken [a]

[a] Department of Civil, Environmental, and Natural Resources Engineering, Luleå University of Technology, 97187 Luleå, Sweden

[b] NCC Sverige AB, Department of Research, and Innovation, 170 80, Solna, Sweden

[c] Department of Food, Agricultural, and Biological Engineering, Ohio State University, Agricultural Engineering Building, 590 Woody Hayes Dr, Columbus, OH, 43210, USA

[d] Department of Civil, Environmental, and Geodetic Engineering, Ohio State University, Hitchcock Hall, 2070 Neil Avenue, Columbus, OH, 43210, USA

[e] Core Faculty, Sustainability Institute, Ohio State University, Smith Lab 174 W, 18th Avenue, Columbus, OH, 43210, USA

[*] Corresponding Author: ali.beryani@ltu.se

Summary: 48 pages, 5 subtopics, 10 figures, 11 tables

Contents

Methodology S4

Occurrence analysis of PFASs and precursors S14

Concentration and distribution analysis of PFASs and precursors S16

Comparison with other studies of urban surface soils and stormwater sediments S37

Redundancy analysis (RDA) S38

References S47

Table of Figures

Figure S1. Comparison of PFAS occurrence frequencies in the forebay (FB) and biofilter material (FM) S14

Figure S2. Comparison of PFAS occurrence frequencies in the forebay (FB) and biofilter material (FM) after the oxidation process by TOP assay S14

Figure S3. Occurrence frequency of PFASs in different parts of the biofilter media S15

Figure S4. Occurrence frequency of PFASs in different parts of the biofilter media after the oxidation process by TOP assay S15

Figure S5. Concentration distribution of other PFASs at forebays (FB) and biofilter materials at different depths (D1−D3) and distances from the inlet (L1 and L2). Cross and circle symbols represent censored and quantified data, respectively. Dashed lines represent LoQ of substances S17

Figure S6. PFAS, PFAS (TOP), and ΔPFAS (increase after TOP) concentrations and accumulation distribution for each site S22

Figure S7. Concentrations of frequently quantified PFASs (before and after TOP) versus filter media depth in 20 sites studied S33

Figure S8. The geometric mean of the ratio of PFAS concentrations at D1 and D3 among 20 sites for two distances from the inlet S33

Figure S9. Heat map of Kendell's Tau correlation scores for environmental parameters and most frequently quantified PFASs (before and after TOP assay) and calculated unknown precursors (PFASs (Pre)) (p-values in Table S9) S35

Figure S10. Data treatment process for RDA S38

Table of tables

Table S1-1. Site characteristics S4
Table S1-2. Site characteristics (continued): media compositions and hydraulic parameters S5

Table S2. Description of the analytical methods for PFASs and soil parameters S10

Table S3. Chemical characteristics of PFASs and their limits of quantification (LoQ) in the two analytical methods S11

Table S4. K_{OC} or Kd (L/kg) of PFASs estimated by different studies in soil and sediment environments S12

Table S5. Statistical summary of occurrence frequency (%) and concentration (µg/kg-DW) of 35 targeted PFASs S23

Table S6. Statistical summary of occurrence frequency (%) and concentration (µg/kg-DW) of 31 targeted PFASs after the oxidation process by TOP assay S24

Table S7. P-values of the significance of difference tests (acceptancy p-value: 0.05) between different sampling points for the concentrations of the most frequently found PFASs, PFAAs after TOP assay, and the estimated total precursors (Pre) (Peto&Peto modification of Wilcoxon test in "cendiff" function in the NADA package was used for the series including censored data, and "Wilcoxon test" otherwise) S25

Table S8. Percentage of Increase in PFAA concentrations after TOP assay relative to their targeted concentration (ΔPFAA/PFAA*100) S33

Table S9. P-values for Kendell's Tau correlations shown in Figure S8 ....S36

Table S10. Comparing the concentrations of most frequently found PFASs (minimum; median/mean; maximum in µg/kg-DW) in this study with those in various urban soil/sediment media reported by other studies S37

Table S11. Final RDA model's report summary S38

Methodology

Table S1-1. Site characteristics

									Estimated hydraulic parameters using a simplified Green-Ampt model based on soil type, moisture content, and surface intake rate (SIR) data, along with assumed typical values of effective porosity (ε_e) and wetting-front suction head for a certain soil type. Saturated hydraulic conductivity (K_S) and ε_e represent the average values of all points: 3 depths x 2 locations. The range of pore velocity (V_{pore}) and contact time were calculated assuming a ponding depth ranging from zero to 30 cm, the maximum value specified in Ohio State's biofilter design criteria,[1] under saturation conditions.							
									Using the lower limit of the typical range of ε_e				Using the Upper limit of the typical range of ε_e			
**Site #**	**Place**	**Catchment type**	**Age at sampling time (yr)**	**Catchment area; CA (m²)**	**Filter area; FA (m²)**	**FA/CA ratio (%)**	**Forebay**	**Filter media depth (cm)**	**ε_e - ave.**	**K_S - ave. (cm/hr) (Green&Ampt)**	**V_{pore} (cm/hr)**	**Empty bed contact time (min)**	**ε_e - ave.**	**K_S - ave. (cm/hr) (Green&Ampt)**	**$V_{pore.}$ (cm/hr)**	**Empty bed contact time (min)**
**1**	Akron, OH	Parking/Roads	13	6500	180	2.77	Yes	45	0.35	40	61 - 102	14.4 - 23.4	0.48	29	116 - 194	27 - 45
**2**	Columbus, OH	Parking/Roads	9	4500	580	12.89	Yes	50	0.34	138	183 - 295	4.8 - 7.8	0.48	89	408 - 657	10.2 - 16.8
**3**	Upper Arlington, OH	Residential	9	1253311	1200	0.10	Yes	50	0.33	147	216 - 347	4.8 - 7.2	0.49	106	442 - 712	9 - 14.4
**4**	Hamilton, OH	Industrial	12	4500	200	4.44	Yes	50	0.35	79	134 - 216	8.4 - 13.8	0.48	64	224 - 361	14.4 - 22.8
**5**	Hamilton, OH	Industrial	12	4500	200	4.44	Yes	50	0.35	125	202 - 326	5.4 - 9	0.48	97	355 - 571	9.6 - 15
**6**	Upper Arlington, OH	Residential	9	223791	900	0.40	Yes	50	0.33	368	460 - 740	1.8 - 3	0.49	226	1115 - 1794	4.2 - 6.6
**7**	Upper Arlington, OH	Residential	9	145687	1900	1.30	Yes	50	0.34	84	120 - 192	7.8 - 12.6	0.49	58	246 - 397	15.6 - 25.2
**8**	Westerville, OH	Parking/Roads	8	12000	600	5.00	Yes	32.5	0.31	61	80 - 155	5.4 - 10.2	0.50	40	193 - 374	13.2 - 24.6
**9**	Westerville, OH	Parking/Roads	8	2000	50	2.50	Yes	37.5	0.31	10	9 - 16	38.4 - 69.6	0.50	4	33 - 59	145.2 - 262.8
**10**	Fort Wright, KY	Commercial	16	3000	190	6.33	No	50	0.29	13	5 - 8	43.2 - 69.6	0.54	3	43 - 69	365.4 - 588
**11**	Ann Arbor, MI	Parking/Roads	14	2250	156	6.93	Yes	50	0.35	32	47 - 76	20.4 - 33	0.48	23	92 - 147	40.2 - 64.2
**12**	Toledo, OH	Residential	9	250	50	20.00	No	50	0.33	91	106 - 171	7.2 - 11.4	0.48	51	273 - 439	18 - 28.8
**13**	Seven Hills, OH	Commercial	11	1200	200	16.67	No	50	0.33	50	58 - 93	12.6 - 20.4	0.49	28	151 - 244	32.4 - 52.2
**14**	Kent, OH	Fueling station	11	800	70	8.75	Yes	50	0.31	11	10 - 16	50.4 - 81	0.53	5	37 - 60	193.8 - 311.4
**15**	North Canton, OH	Fueling station	12	1250	180	14.40	Yes	50	0.35	43	59 - 95	15.6 - 24.6	0.48	28	123 - 199	31.8 - 51
**16**	North Canton, OH	Fueling station	12	1000	100	10.00	Yes	50	0.35	23	36 - 58	29.4 - 46.8	0.48	17	65 - 104	52.2 - 83.4
**17**	Parma, OH	Fueling station	8	2500	200	8.00	Yes	50	0.31	-	-	-	0.51	-	-	-
**18**	Twinsburg, OH	Fueling station	13	2000	70	3.50	No	35	0.31	48	61 - 113	7.8 - 13.8	0.50	30	154 - 289	18.6 - 34.8
**19**	Orange Village, OH	Residential	10	250	20	8.00	No	50	0.33	23	26 - 42	27 - 43.2	0.50	13	70 - 112	72.6 - 116.4
**20**	Orange Village, OH	Residential	10	250	20	8.00	No	50	0.35	32	50 - 81	21 - 33.6	0.48	24	90 - 145	37.2 - 60

Table S1-2. Site characteristics (continued): media compositions and hydraulic parameters

Site #	Sampling Location	% Gravel*	%OM*	Mineral fraction % sand*	Mineral fraction % silt*	Mineral fraction % clay*	Total fraction % gravel*	Total fraction % OM*	Total fraction % sand*	Total fraction % silt*	Total fraction % clay*	Soil Texture	Water content (%mass)	Surface intake rate (cm/hr) - Infiltrometer Test 1	Surface intake rate (cm/hr) - Infiltrometer Test 2 (right after Test 1)	Typical effective porosity - ε_e	Typical residual moisture	Typical wetting front suction (cm)	Estimated K_S (cm/hr) using Green&Ampt model
1	Forebay	15.71	8.06	91.92	6.01	2.07	24.00	10.37	60.33	3.94	1.36	Sa	6.30						
1	L1-D1	5.37	7.88	85.14	11.34	3.52	8.13	11.30	68.60	9.14	2.84	LoSa	22.50			0.33 - 0.47	0.04	13.00	
1	L1-D2	10.99	3.92	87.98	7.84	4.18	17.33	5.50	67.89	6.05	3.23	Sa	14.30	41.87	23.50	0.35 - 0.48	0.02	10.00	20.6 - 31.2
1	L1-D3	13.32	1.32	94.37	3.48	2.15	19.43	1.67	74.46	2.75	1.70	Sa	14.10			0.35 - 0.48	0.02	10.00	
1	L2-D1	13.36	2.62	89.28	7.18	3.55	19.48	3.31	68.93	5.54	2.74	Sa	11.10			0.35 - 0.48	0.02	10.00	
1	L2-D2	12.01	2.11	90.93	5.16	3.91	17.69	2.74	72.35	4.11	3.11	Sa	10.70	78.42	76.71	0.35 - 0.48	0.02	10.00	37.3 - 48.6
1	L2-D3	12.47	1.59	91.90	4.76	3.34	21.00	2.35	70.44	3.65	2.56	Sa	16.50			0.35 - 0.48	0.02	10.00	
2	Forebay	6.24	5.75	74.19	19.94	5.87	11.99	10.35	57.61	15.49	4.56	SaLo	27.50						
2	L1-D1	4.85	5.19	72.23	20.47	7.30	8.90	9.05	59.27	16.80	5.99	SaLo	22.50			0.28 - 0.54	0.04	22.00	
2	L1-D2	11.58	2.43	87.86	9.28	2.86	19.24	3.57	67.82	7.16	2.21	Sa	15.50	109.73	68.15	0.35 - 0.48	0.02	10.00	46.3 - 88
2	L1-D3	10.12	2.59	80.99	13.72	5.30	16.10	3.71	64.94	11.00	4.25	LoSa	18.60			0.33 - 0.47	0.04	13.00	
2	L2-D1	6.33	4.01	78.90	15.03	6.07	11.56	6.87	64.36	12.26	4.95	LoSa	18.00			0.33 - 0.47	0.04	13.00	
2	L2-D2	11.60	2.12	89.97	7.33	2.70	18.27	2.96	70.87	5.77	2.13	Sa	12.20	268.94	79.51	0.35 - 0.48	0.02	10.00	131 - 187.6
2	L2-D3	9.17	2.29	88.09	8.81	3.10	15.13	3.44	71.74	7.18	2.52	Sa	19.40			0.35 - 0.48	0.02	10.00	
3	Forebay	38.31	2.93	93.53	4.04	2.43	55.90	2.64	38.78	1.67	1.01	Sa	6.80						
3	L1-D1	22.89	4.37	91.61	5.86	2.53	35.04	5.16	54.79	3.50	1.51	Sa	14.10			0.35 - 0.48	0.02	10.00	
3	L1-D2	9.99	6.99	87.18	9.38	3.44	16.72	10.53	63.42	6.82	2.50	Sa	18.90	291.21	140.25	0.35 - 0.48	0.02	10.00	147.2 - 201.2
3	L1-D3	22.99	3.26	87.85	7.88	4.26	37.35	4.08	51.46	4.62	2.50	Sa	15.80			0.35 - 0.48	0.02	10.00	
3	L2-D1	17.22	6.50	87.20	8.17	4.64	27.75	8.67	55.44	5.19	2.95	LoSa	12.70			0.33 - 0.47	0.04	13.00	
3	L2-D2	26.43	4.29	92.82	5.85	1.33	41.54	4.97	49.66	3.13	0.71	Sa	19.20	142.12	88.63	0.35 - 0.48	0.02	10.00	65 - 93.1
3	L2-D3	25.66	2.46	86.84	8.43	4.73	39.69	2.82	49.92	4.84	2.72	LoSa	14.80			0.33 - 0.47	0.04	13.00	
4	Forebay	7.77	7.63	52.72	37.79	9.49	12.07	10.94	40.59	29.10	7.30	SaLo	19.70						
4	L1-D1	8.10	3.33	86.61	10.56	2.83	12.61	4.77	71.55	8.72	2.34	Sa	16.20			0.35 - 0.48	0.02	10.00	
4	L1-D2	10.90	1.47	95.41	3.63	0.96	15.80	1.90	78.52	2.99	0.79	Sa	5.20	97.28	101.60	0.35 - 0.48	0.02	10.00	43.4 - 54.6
4	L1-D3	9.31	1.28	97.96	1.54	0.50	13.10	1.63	83.53	1.32	0.43	Sa	3.60			0.35 - 0.48	0.02	10.00	
4	L2-D1	12.52	2.24	93.57	4.83	1.59	18.52	2.90	73.53	3.80	1.25	Sa	10.40			0.35 - 0.48	0.02	10.00	
4	L2-D2	10.73	1.42	97.01	2.32	0.68	15.61	1.84	80.08	1.91	0.56	Sa	4.60	197.35	149.09	0.35 - 0.48	0.02	10.00	85.3 - 104.1
4	L2-D3	10.26	1.35	97.80	1.66	0.53	14.84	1.75	81.58	1.39	0.45	Sa	5.00			0.35 - 0.48	0.02	10.00	

5	Forebay	9.11	9.04	72.71	21.88	5.41	13.84	12.49	53.56	16.12	3.99	SaLo	15.00						
5	L1-D1	7.79	2.50	89.95	8.05	2.00	11.31	3.35	76.77	6.87	1.71	Sa	17.50			0.35 - 0.48	0.02	10.00	
5	L1-D2	7.82	1.84	93.66	4.93	1.41	11.38	2.48	80.68	4.25	1.22	Sa	4.10	182.88	142.88	0.35 - 0.48	0.02	10.00	82.1 - 104.1
5	L1-D3	8.21	1.02	98.25	1.30	0.46	11.66	1.33	85.49	1.13	0.40	Sa	4.10			0.35 - 0.48	0.02	10.00	
5	L2-D1	7.54	3.85	91.96	6.75	1.29	11.68	5.52	76.14	5.59	1.07	Sa	19.90			0.35 - 0.48	0.02	10.00	
5	L2-D2	7.29	1.99	94.58	4.38	1.04	10.96	2.78	81.58	3.78	0.90	Sa	4.20	244.93	146.30	0.35 - 0.48	0.02	10.00	111.9 - 145.2
5	L2-D3	8.20	1.23	97.92	1.62	0.46	11.75	1.62	84.83	1.41	0.40	Sa	3.90			0.35 - 0.48	0.02	10.00	
6	Forebay	20.87	2.40	94.40	4.33	1.27	30.52	2.77	62.97	2.89	0.85	Sa	9.60						
6	L1-D1	17.80	6.03	70.91	22.39	6.70	29.53	8.23	44.14	13.93	4.17	SaLo	44.00			0.28 - 0.54	0.04	22.00	154.9 - 241.7
6	L1-D2	12.87	8.20	87.60	9.30	3.10	22.39	12.43	57.10	6.06	2.02	Sa	30.20	273.77	95.25	0.35 - 0.48	0.02	10.00	
6	L1-D3	20.62	1.94	87.84	9.25	2.91	37.49	2.80	52.45	5.52	1.74	Sa	14.50			0.35 - 0.48	0.02	10.00	
6	L2-D1	1.62	13.11	54.19	36.03	9.77	3.38	26.84	37.81	25.14	6.82	SaLo	41.60			0.28 - 0.54	0.04	22.00	296.8 - 493.7
6	L2-D2	18.57	6.53	77.22	17.08	5.69	30.99	8.87	46.45	10.27	3.42	LoSa	11.50	672.35	392.45	0.33 - 0.47	0.04	13.00	
6	L2-D3	24.14	4.00	84.03	11.60	4.37	39.53	4.96	46.65	6.44	2.43	LoSa	15.80			0.33 - 0.47	0.04	13.00	
7	Forebay	25.51	2.74	91.19	6.64	2.16	39.28	3.14	52.50	3.83	1.24	Sa	8.00						
7	L1-D1	11.97	5.56	83.12	13.33	3.55	21.86	8.95	57.51	9.22	2.46	LoSa	18.40			0.33 - 0.47	0.04	13.00	110.5 - 154.6
7	L1-D2	19.23	3.32	89.80	7.88	2.32	31.61	4.41	57.45	5.04	1.48	Sa	16.10	234.46	141.11	0.35 - 0.48	0.02	10.00	
7	L1-D3	22.48	2.51	90.98	6.80	2.22	36.39	3.15	55.00	4.11	1.34	Sa	9.70			0.35 - 0.48	0.02	10.00	
7	L2-D1	5.70	5.97	71.42	22.32	6.26	12.04	11.88	54.33	16.98	4.76	SaLo	38.50	21.77	12.60	0.28 - 0.54	0.04	22.00	5.4 - 13.2
7	L2-D2	20.13	2.45	89.25	7.79	2.96	32.41	3.15	57.51	5.02	1.91	Sa	13.10			0.35 - 0.48	0.02	10.00	
7	L2-D3	19.24	2.30	88.57	8.75	2.68	30.43	2.94	59.01	5.83	1.79	Sa	15.00			0.35 - 0.48	0.02	10.00	
8	Forebay	15.14	7.31	93.69	4.75	1.57	23.41	9.60	62.77	3.18	1.05	Sa	8.50						
8	L1-D1	19.41	10.79	49.59	41.40	9.01	37.64	16.87	22.56	18.83	4.10	Lo	58.20			0.33 - 0.53	0.03	24.00	
8	L1-D2	30.55	4.25	84.53	5.71	9.76	49.35	4.76	38.79	2.62	4.48	LoSa	15.80	94.66	74.04	0.33 - 0.47	0.04	13.00	61.5 - 83.6
8	L1-D3	29.86	2.80	79.84	14.82	5.34	46.91	3.09	39.92	7.41	2.67	LoSa	23.50			0.33 - 0.47	0.04	13.00	
8	L2-D1	21.14	4.12	80.64	14.20	5.16	34.20	5.25	48.83	8.60	3.12	LoSa	19.70			0.33 - 0.47	0.04	13.00	
8	L2-D2	30.35	2.79	79.86	12.05	8.09	49.36	3.16	37.92	5.72	3.84	LoSa	17.40	45.72	38.10	0.33 - 0.47	0.04	13.00	18.7 - 37.7
8	L2-D3	25.99	2.57	63.70	19.48	16.82	41.38	3.03	35.41	10.83	9.35	SaLo	22.40			0.28 - 0.54	0.04	22.00	
9	Forebay	21.58	7.84	77.63	17.00	5.37	35.68	10.16	42.04	9.21	2.91	LoSa	21.70						
9	L1-D1	22.07	6.15	60.35	30.08	9.56	40.91	8.88	30.30	15.10	4.80	SaLo	33.10			0.28 - 0.54	0.04	22.00	
9	L1-D2	37.32	1.45	77.56	15.19	7.25	66.73	1.63	24.54	4.81	2.29	LoSa	14.80	15.24	22.86	0.33 - 0.47	0.04	13.00	4.2 - 10.2
9	L1-D3	34.91	1.69	84.35	11.22	4.44	54.64	1.72	36.81	4.89	1.94	LoSa	16.50			0.33 - 0.47	0.04	13.00	
9	L2-D1	22.73	3.72	69.89	19.29	10.82	38.47	4.86	39.60	10.93	6.13	SaLo	17.40			0.28 - 0.54	0.04	22.00	
9	L2-D2	33.51	1.67	81.40	11.72	6.87	54.06	1.80	35.94	5.17	3.03	LoSa	12.70	-	-	0.33 - 0.47	0.04	13.00	-
9	L2-D3	23.98	1.93	79.98	14.02	6.00	37.70	2.31	47.98	8.41	3.60	LoSa	22.40			0.33 - 0.47	0.04	13.00	
10	L1-D1	4.46	13.21	55.02	35.85	9.12	9.81	27.78	34.34	22.38	5.69	SaLo	49.30	11.43	10.16	0.28 - 0.54	0.04	22.00	2.8 - 11.4

10	L1-D2	1.36	3.01	70.32	23.99	5.69	2.29	5.02	65.19	22.24	5.27	SaLo	21.60			0.28 - 0.54	0.04	22.00	
10	L1-D3	2.17	2.39	68.43	23.99	7.58	3.80	4.09	63.03	22.10	6.98	SaLo	19.40			0.28 - 0.54	0.04	22.00	
10	L2-D1	4.83	5.79	50.01	40.16	9.83	9.27	10.58	40.08	32.19	7.88	Lo	31.80			0.33 - 0.53	0.03	24.00	
10	L2-D2	2.10	2.78	68.03	24.94	7.03	3.70	4.79	62.26	22.82	6.43	SaLo	20.50	13.97	5.08	0.28 - 0.54	0.04	22.00	2.7 - 14
10	L2-D3	1.45	2.59	68.04	21.66	10.30	2.51	4.41	63.34	20.16	9.59	SaLo	22.50			0.28 - 0.54	0.04	22.00	
11	Forebay	2.93	8.47	80.69	14.66	4.65	6.47	18.14	60.84	11.06	3.50	LoSa	57.80						
11	L1-D1	5.69	6.38	90.77	7.30	1.93	9.43	9.97	73.16	5.88	1.56	Sa	36.20			0.35 - 0.48	0.02	10.00	
11	L1-D2	5.81	5.53	86.19	10.28	3.53	9.88	8.87	70.03	8.35	2.87	LoSa	20.20	39.70	71.25	0.33 - 0.47	0.04	13.00	23.8 - 32.8
11	L1-D3	8.01	2.22	91.12	6.79	2.09	12.40	3.16	76.94	5.73	1.77	Sa	15.80			0.35 - 0.48	0.02	10.00	
11	L2-D1	6.46	5.95	88.71	8.38	2.91	12.04	10.37	68.83	6.50	2.26	Sa	25.40			0.35 - 0.48	0.02	10.00	
11	L2-D2	11.91	2.64	91.78	6.45	1.77	18.46	3.60	71.53	5.03	1.38	Sa	18.50	39.76	24.13	0.35 - 0.48	0.02	10.00	21.2 - 31
11	L2-D3	10.53	1.92	92.65	5.24	2.12	15.99	2.60	75.42	4.26	1.72	Sa	11.60			0.35 - 0.48	0.02	10.00	
12	L1-D1	0.87	7.45	74.13	19.84	6.03	1.54	12.99	63.36	16.95	5.16	SaLo	30.70			0.28 - 0.54	0.04	22.00	
12	L1-D2	4.30	5.04	80.93	15.68	3.39	7.01	7.86	68.90	13.35	2.88	LoSa	24.50	77.93	69.45	0.33 - 0.47	0.04	13.00	36.6 - 70.8
12	L1-D3	0.37	3.39	89.00	9.77	1.23	0.59	5.40	83.67	9.19	1.16	Sa	17.80			0.35 - 0.48	0.02	10.00	
12	L2-D1	0.78	5.34	79.74	17.13	3.12	1.51	10.31	70.31	15.11	2.76	LoSa	28.10			0.33 - 0.47	0.04	13.00	
12	L2-D2	1.30	4.86	81.25	15.23	3.51	2.23	8.21	72.77	13.64	3.15	LoSa	22.70	125.83	63.06	0.33 - 0.47	0.04	13.00	66.1 - 110.5
12	L2-D3	2.28	2.70	91.11	7.72	1.17	3.80	4.40	83.63	7.09	1.07	Sa	15.20			0.35 - 0.48	0.02	10.00	
13	L1-D1	9.42	7.92	70.72	21.84	7.44	16.54	12.59	50.12	15.48	5.27	SaLo	38.20			0.28 - 0.54	0.04	22.00	
13	L1-D2	9.11	3.89	91.45	6.54	2.01	14.84	5.75	72.62	5.20	1.60	Sa	26.30	74.58	24.83	0.35 - 0.48	0.02	10.00	38.7 - 66.4
13	L1-D3	13.82	3.36	90.85	7.00	2.15	22.26	4.67	66.39	5.12	1.57	Sa	15.60			0.35 - 0.48	0.02	10.00	
13	L2-D1	3.76	7.11	75.95	17.50	6.55	7.46	13.59	59.96	13.82	5.17	SaLo	34.50			0.28 - 0.54	0.04	22.00	
13	L2-D2	5.76	5.87	86.35	9.56	4.09	10.01	9.63	69.39	7.68	3.29	LoSa	23.20	38.91	17.78	0.33 - 0.47	0.04	13.00	18.1 - 34.4
13	L2-D3	7.71	3.49	92.88	5.28	1.84	12.59	5.26	76.30	4.34	1.51	Sa	14.50			0.35 - 0.48	0.02	10.00	
14	Forebay	20.89	6.98	86.03	8.54	5.44	32.99	8.72	50.15	4.98	3.17	LoSa	11.50						
14	L1-D1	5.08	15.17	38.51	37.38	24.11	11.19	31.73	21.98	21.33	13.76	Lo	48.60			0.33 - 0.53	0.03	24.00	
14	L1-D2	9.52	6.43	73.37	16.43	10.20	16.94	10.36	53.34	11.95	7.41	SaLo	32.90	4.47	2.54	0.28 - 0.54	0.04	22.00	1 - 3.6
14	L1-D3	14.33	2.96	80.14	12.63	7.23	23.35	4.12	58.12	9.16	5.24	LoSa	19.30			0.33 - 0.47	0.04	13.00	
14	L2-D1	4.80	18.68	31.47	41.19	27.35	10.97	40.64	15.23	19.93	13.23	Lo	69.40			0.33 - 0.53	0.03	24.00	
14	L2-D2	5.53	9.28	59.44	25.34	15.22	10.00	15.85	44.07	18.79	11.29	SaLo	27.70	19.05	21.59	0.28 - 0.54	0.04	22.00	9.3 - 19.1
14	L2-D3	8.77	3.30	76.08	15.13	8.79	13.89	4.77	61.88	12.31	7.15	SaLo	20.00			0.28 - 0.54	0.04	22.00	
15	Forebay	9.52	4.76	77.76	15.58	6.66	21.44	9.70	53.54	10.73	4.59	LoSa	49.90						
15	L1-D1	3.70	5.87	76.16	18.19	5.65	6.96	10.63	62.76	14.99	4.66	LoSa	35.50		0.00	0.33 - 0.47	0.04	13.00	
15	L1-D2	8.77	1.30	90.02	8.04	1.94	13.77	1.86	75.94	6.79	1.64	Sa	10.90	1.27		0.35 - 0.48	0.02	10.00	0.1 - 0.5
15	L1-D3	6.13	1.22	90.60	7.48	1.92	9.76	1.81	80.12	6.61	1.70	Sa	11.80			0.35 - 0.48	0.02	10.00	

15	L2-D1	2.60	4.76	82.63	14.73	2.64	4.56	8.13	72.14	12.86	2.30	LoSa	23.30			0.33 - 0.47	0.04	13.00	
15	L2-D2	6.16	1.52	91.26	7.11	1.63	9.95	2.30	80.08	6.24	1.43	Sa	9.40	119.28	73.86	0.35 - 0.48	0.02	10.00	56.3 - 84.8
15	L2-D3	7.67	1.20	91.44	6.88	1.68	12.20	1.77	78.67	5.92	1.45	Sa	10.00			0.35 - 0.48	0.02	10.00	
16	Forebay	25.80	4.09	82.80	12.55	4.65	40.10	4.71	45.70	6.93	2.57	LoSa	21.50						
16	L1-D1	5.47	6.26	76.88	17.26	5.86	9.70	10.50	61.35	13.77	4.68	LoSa	31.20			0.33 - 0.47	0.04	13.00	
16	L1-D2	5.80	1.60	91.99	5.93	2.08	8.94	2.33	81.62	5.26	1.85	Sa	10.40	2.54	3.81	0.35 - 0.48	0.02	10.00	0.3 - 1.1
16	L1-D3	25.62	1.21	92.96	4.88	2.16	39.07	1.37	55.36	2.91	1.29	Sa	12.10			0.35 - 0.48	0.02	10.00	
16	L2-D1	4.06	2.50	86.94	9.79	3.27	6.37	3.76	78.13	8.80	2.94	Sa	14.80			0.35 - 0.48	0.02	10.00	
16	L2-D2	6.79	1.42	93.20	5.14	1.66	10.40	2.03	81.61	4.50	1.45	Sa	11.30	72.19	38.10	0.35 - 0.48	0.02	10.00	34.2 - 44.3
16	L2-D3	9.24	1.32	91.84	6.29	1.87	14.38	1.86	76.93	5.27	1.57	Sa	11.70			0.35 - 0.48	0.02	10.00	
17	Forebay	1.52	8.35	54.44	35.20	10.36	3.35	18.18	42.72	27.62	8.13	SaLo	47.20						
17	L1-D1	3.08	11.14	57.25	29.30	13.44	7.37	25.79	38.27	19.59	8.99	SaLo	56.50			0.28 - 0.54	0.04	22.00	
17	L1-D2	1.90	8.31	60.31	27.10	12.59	4.20	18.04	46.89	21.07	9.79	SaLo	51.70	1.27	0.00	0.28 - 0.54	0.04	22.00	0.5 - 1.3
17	L1-D3	16.42	4.97	65.39	24.27	10.34	29.84	7.55	40.94	15.20	6.47	SaLo	29.20			0.28 - 0.54	0.04	22.00	
17	L2-D1	2.68	9.86	66.75	23.46	9.79	5.49	19.65	49.97	17.56	7.33	SaLo	49.80			0.28 - 0.54	0.04	22.00	
17	L2-D2	5.85	2.29	89.81	7.00	3.19	9.19	3.39	78.51	6.12	2.79	Sa	20.10	3.15	0.00	0.35 - 0.48	0.02	10.00	0.5 - 1.6
17	L2-D3	8.12	1.04	95.62	3.27	1.10	12.03	1.42	82.77	2.83	0.96	Sa	7.70			0.35 - 0.48	0.02	10.00	
18	L1-D1	5.27	9.73	70.76	22.59	6.65	10.42	18.23	50.48	16.11	4.74	SaLo	41.10			0.28 - 0.54	0.04	22.00	
18	L1-D2	19.72	2.41	85.40	11.04	3.56	33.85	3.32	53.65	6.94	2.24	LoSa	15.00	68.58	32.36	0.33 - 0.47	0.04	13.00	30.9 - 52.6
18	L1-D3	10.63	1.80	83.17	12.33	4.50	17.52	2.66	66.39	9.84	3.59	LoSa	16.70			0.33 - 0.47	0.04	13.00	
18	L2-D1	5.13	10.26	64.19	28.00	7.82	12.79	24.26	40.41	17.63	4.92	SaLo	55.20			0.28 - 0.54	0.04	22.00	
18	L2-D2	8.82	7.27	74.35	19.78	5.87	19.27	14.49	49.25	13.10	3.89	SaLo	40.10	49.87	34.29	0.28 - 0.54	0.04	22.00	30 - 42.6
18	L2-D3	21.98	2.73	82.74	12.15	5.11	36.61	3.55	49.51	7.27	3.06	LoSa	11.80			0.33 - 0.47	0.04	13.00	
19	L1-D1	0.56	6.03	75.37	18.17	6.46	1.29	13.94	63.89	15.40	5.48	SaLo	32.00			0.28 - 0.54	0.04	22.00	
19	L1-D2	12.99	2.86	95.64	3.27	1.09	24.13	4.63	68.14	2.33	0.77	Sa	22.60	27.30	22.86	0.35 - 0.48	0.02	10.00	12.9 - 23
19	L1-D3	12.76	1.87	96.27	2.94	0.80	22.29	2.85	72.07	2.20	0.60	Sa	13.10			0.35 - 0.48	0.02	10.00	
20	L1-D1	11.00	3.91	92.23	5.74	2.03	21.63	6.83	65.98	4.10	1.45	Sa	32.70			0.35 - 0.48	0.02	10.00	
20	L1-D2	11.64	2.16	90.91	6.99	2.09	21.08	3.45	68.61	5.28	1.58	Sa	12.80	43.54	46.33	0.35 - 0.48	0.02	10.00	24.2 - 31.9
20	L1-D3	11.04	1.82	92.00	6.33	1.67	19.25	2.82	71.70	4.93	1.30	Sa	10.60			0.35 - 0.48	0.02	10.00	

*

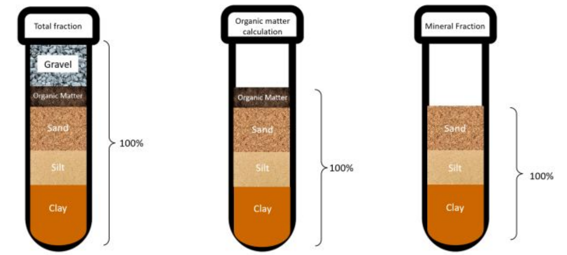
Total fraction
Gravel
Organic Matter
Sand
Silt
Clay
100%
Organic matter calculation
Organic Matter
Sand
Silt
Clay
100%
Mineral Fraction
Sand
Silt
Clay
100%

Table S2. Description of the analytical methods for PFASs and soil parameters

**Targeted 35 PFASs**	**Accredited laboratory:** Eurofins Water Testing Sweden AB<br>**Standard method/Reference:**<br>- DIN 38414-14 mod.[2]<br>- Anal. Chem.2005,77,6353−6358 mod.[3]<br>**Modifications to the reference methods:**<br>- Higher sample weight (20 g vs. 5 g).<br>- Minor differences in analysis execution up to the final extract.<br>- Modifications to instrument parameters, including column, mobile phase, flow, gradient, and detection parameters, due to technical advancements since the publication of the reference methods (2011 and 2005).<br>- Use of additional internal standards and, in some cases, extended/alternative product ions.<br>- Achieving lower LoQs and targeting a larger number of PFASs compared to the reference methods which had a LoQ of 10 µg/kg-DW and determined only 10 PFASs.<br>**Analysis:** Liquid chromatography-tandem mass spectrometry (UPLC-MS/MS)<br>**Measurement uncertainty:** ±23% with a coverage factor of 2 for all substances<br>**Method's LoQ** = 0.03−1 µg/Kg-DW = 10*S/N, where S/N is the signal-to-noise ratio.
**Total Oxidizable Precursors Assay (TOP)**	**Laboratory:** Eurofins Water Testing Sweden AB<br>**Method/Reference:**<br>- Environ. Sci. Technol. 2012,46,9342−9349.[4]<br>**Analysis:** Liquid chromatography-tandem mass spectrometry (UPLC-MS/MS)<br>**Modifications to the reference methods:**<br>- Higher sample weight (20 g vs. 5 g).<br>- Minor differences in analysis execution up to the final extract.<br>- Modifications to instrument parameters, including column, mobile phase, flow, gradient, and detection parameters, due to technical advancements since the publication of the reference methods (2011 and 2005).<br>- Use of additional internal standards and, in some cases, extended/alternative product ions.<br>- Achieving lower LoQs and targeting a larger number of PFASs compared to the reference methods. **Measurement uncertainty:** ±36% with a coverage factor of 2 for all substances<br>**Method's LoQ** = 0.1−2 µg/Kg-DW = 10*S/N, where S/N is the signal-to-noise ratio.
**Soil composition and loss on ignition (LOI)**	**Analysis:** Traditional sieving method, laser diffraction particle analyzer, and gravimetric analysis<br>**Standard methods/References**:<br>- CSN EN 12879[5]<br>- CSN 720103[6]<br>- CSN 465735[7]<br>- ASTM-D2974−20[8]<br>- Soil Sci. Soc. Am. J. 83:1244–1252[9]<br>- Can. J. Soil Sci. 89:413419[10]<br>**Methodology:**<br>50 mL of soil sample into glass vials → Drying in an oven at 105 ◦C overnight → Sieving using a #10 (2 mm) mesh sieve → weighing the soil retained on the sieve as the gravel percentage of the sample → Analyzing the remaining sample by burning organic matter (OM) in a muffle furnace at 550 ◦C for 24 hr → Recording the sample's loss on ignition (LOI) percentage → The outcome is mineral soil (sand + silt + clay) fraction → Dispersing the mineral fraction in 200 mL of 5% sodium hexametaphosphate for 24 hr → Suspending the mineral soil in the suspension using a VibraCell VCX750 ultrasonic meter (Sonics and Materials Inc., Newtown, CT) pulsing on for 10 sec and off for 5 sec over 5 min[10] → Wet sieving the sample through a #270 (53 µm) mesh → Drying the sand retained on the sieve in the oven at 105 ◦C overnight and weighing it as the mineral sand fraction percentage → Transferring the remaining mineral soil (silt and clay mixture) via micropipettes into a 50 mL centrifuge tube → Spinning this sample for 15-min at the maximum setting of 3200 RPM within a TJ-6 Model centrifuge (Beckman Coulter, Indianapolis, IN) to settle → Placing the vials into an automated preparation workstation with sonication for the LS 13 320 laser diffraction particle size analyzer (Beckman Coulter, Indianapolis IN) → Analyzing and dividing the samples into three replicates using the LS 13 320 laser diffraction particle size analyzer → Converting the resulting volumetric percentage of fines to a gravimetric measurement according to Faé et al., 2019.[9]
**Specific surface area (SSA)**	**Lab:** Lawson Scientific Ltd, UK<br>**Analysis**: Nitrogen gas adsorption in Brunauer–Emmett–Teller (BET); Quantachrome NovaWin for Data Acquisition and Reduction for NOVA Quantachrome instruments<br>**Standard method/Reference**: DIN ISO 9277:2010[11]

**Dry matter (DM)**	**Accredited laboratory:** Eurofins Water Testing Sweden AB<br>**Standard method/Reference:** SS-EN 12880:2000[12]<br>**Analysis:** weighing samples before and after drying in oven at 105 °C overnight<br>**Measurement uncertainty:** ±5% with a coverage factor of 2
**pH**	**Accredited laboratory:** ALS Scandinavia AB<br>**Standard method/Reference:** SS-EN ISO, 2012[13]<br>**Analysis:** pH measurement after suspension in water
**Surface intake rate (SIR)**	**Method/References**:<br>- Nestingen et al. (2018)[14]<br>- Ahmed et al. (2014)[15]<br>**Methodology:**<br>Single-ring falling head infiltrometer tests were conducted concurrently with and at the same locations as the soil sampling in all biofilters. These tests aimed to determine the surface intake rate (serving as a proxy for the infiltration rate), facilitating an understanding of the permeability at the biofilters' surface. A steel ring infiltrometer with a diameter of 28 cm was firmly inserted into the soil surface, ensuring level placement and a wire mesh was positioned atop the infiltrometer to aid in flow dissipation. Pre-wetting involving the application of 3.79 L (one gallon) of water was executed to saturate the soil, followed by the surface intake rate measurement of the time taken to infiltrate 7.57 L (two gallons) of water. The timer was stopped once the soil's surface stopped glistening with ponded water. If all the ponded water had not infiltrated after fifteen minutes, a ruler was used to measure the amount of water that had infiltrated, which was then divided by the time to calculate the surface intake rate.

Table S3. Chemical characteristics of PFASs and their limits of quantification (LoQ) in the two analytical methods

PFAS substance	Full name	Group	Method's LoQ in targeted 35 PFAS analysis (µg/Kg-DW)	Method's LoQ in TOP assay (µg/Kg-DW)	EQS Sediments (µg/kg-DW)	PNEC Sediments - QSAR (µg/kg-DW)
PFBA	Perfluorobutanoic acid (C4)	SC-PFCAs	0.1	2		8.3
PFPeA	Perfluoropentanoic acid (C5)	SC-PFCAs	0.03	2		24.8
PFHxA	Perfluorohexanoic acid (C6)	SC-PFCAs	0.03	0.1	7602	
PFHpA	Perfluoroheptanoic acid (C7)	SC-PFCAs	0.03	0.1		53.5
PFOA	Perfluorooctanoic acid (C8)	LC-PFCAs	0.03	0.1	15	
PFNA	Perfluorononanoic acid (C9)	LC-PFCAs	0.03	0.1	142	
PFDA	Perfluorodecanoic acid (C10)	LC-PFCAs	0.1	0.1		3.51
PFUdA	Perfluoroundecanoic acid (C11)	LC-PFCAs	0.1	0.2		103
PFDoA	Perfluorododecanoic acid (C12)	LC-PFCAs	0.1	0.2		485
PFTrDA	Perfluorotridecanoic acid (C13)	LC-PFCAs	0.1	1		940
PFTeDA	Perfluorotetradecanoic acid (C14)	LC-PFCAs	0.03	1		940
PFHxDA	Perfluorohexadecanoic acid (C16)	LC-PFCAs	0.03	1		583
HPFHpA	7H-perfluoroheptanoic acid	SC-PFCAs	0.1	NA		41.1
P37DMOA	perfluoro-3,7-dimethyloctanoic acid	LC-PFCAs	0.5	1		5.34
PFBS	Perfluorobutane sulfonic acid (C4)	SC-PFSAs	0.03	0.1	9627	37.08
PFPeS	Perfluoropentane sulfonic acid (C5)	SC-PFSAs	0.1	NA		108
PFHxS	Perfluorohexane sulfonic acid (C6)	LC-PFSAs	0.03	0.1		101
PFHpS	Perfluoroheptane sulfonic acid (C7)	LC-PFSAs	0.03	0.1		30
PFOS	Perfluorooctane sulfonic acid (C8)	LC-PFSAs	0.03	0.1	0.012	
PFNS	Perfluorononane sulfonic acid (C9)	LC-PFSAs	0.2	NA		5.95
PFDS	Perfluorodecane sulfonic acid (C10)	LC-PFSAs	0.03	0.2		154
PFUnDS	Perfluoroundecane sulfonic acid (C11)	LC-PFSAs	1	NA		
PFDoS	Perfluorododecane sulfonic acid (C12)	LC-PFSAs	1	NA		1333
PFTrDS	Perfluorotridecane sulfonic acid (C13)	LC-PFSAs	1	NA		

4:2 FTS	4:2 fluorotelomer sulfonic acid	FTSAs	0.03	0.1		
6:2 FTS	6:2 fluorotelomer sulfonic acid	FTSAs	0.03	0.1		30.1
8:2 FTS	8:2 fluorotelomer sulfonic acid	FTSAs	0.1	0.2		21.4
EtFOSA	N-ethyl perfluorooctane sulfonamide	PFASAs	0.2	1		3.46
MeFOSA	N-methyl perfluorooctane sulfonamide	PFASAs	0.03	1		1.89
EtFOSE	N-ethyl perfluorooctane sulfonamido ethanol	PFASAs	0.1	1		337
MeFOSE	N-methyl perfluorooctane sulfonamido ethanol	PFASAs	0.03	1		118
EtFOSAA	N-ethyl perfluorooctane sulfonamido acetic acid	PFASAs	0.1	1		1200
MeFOSAA	N-methyl perfluorooctane sulfonamido acetic acid	PFASAs	0.03	1		302
FOSAA	Perfluorooctane sulfonamido acetic acid	PFASAs	0.1	1		98
PFOSA	Perfluorooctane sulfonamide	FASAs	0.1	0.1		5.71

Table S4. K_{OC} or Kd (L/kg) of PFASs estimated by different studies in soil and sediment environments

parameter	Log K_{OC} (Higgins and Luthy, 2006)[16]	Log K_{OC} (Ahrens et al., 2011)[17]	Log K_{OC} (Ahrens et al., 2016)[18]	Log K_{OC} (Dalahmeh et al., 2016)[19]	Log K_{OC} (Aly et al., 2018)[20]	Log K_d (Barzen-Hanson et al. 2017)[21]	Log K_d (Ruyle et al., 2023)[22]	Field-derived Log K_d (Li et al., 2018)[23]
PFBA								
PFPeA			1.95					
PFHxA			1.91	2.4–2.7				
PFHpA			2.19	2.6	2			
PFOA	2.06	2.2–2.6	2.31	2.3–2.7	1.92		0.25–0.33	1.16 (median)
PFNA	2.39		2.33	2.8–3.3	1.72			1.41 (median)
PFDA	2.76		3.17					1.85 (median)
PFUdA	3.3		3.3	3.2–3.5				
PFDoA								
PFTrDA								
PFTeDA								
PFHxDA								
HPFHpA								
P37DMOA								
PFBS			2.5	2.2–2.5	1.8		0.02–0.05	
PFPeS								
PFHxS			2.7		2		0.04–0.11	
PFHpS								
PFOS	2.57	3–3.9	3.34	3.2–3.6	2.48		0.6–1.5	1.92 (median)
PFNS								
PFDS	3.53		3.53					
PFUnDS								
PFDoS								
PFTrDS								

4:2 FTS								
6:2 FTS						0.6–1.2		
8:2 FTS						1.4–2.1		
EtFOSA								
MeFOSA								
EtFOSE								
MeFOSE								
EtFOSAA	3.23		3.23					
MeFOSAA	3.11		3.11					
FOSAA								
PFOSA		3.7–5						
C4 PFSA precursors							0.06–0.2	
C6 PFSA precursors							0.63–1.7	
C8 PFSA precursors							5.2–14	

Occurrence analysis of PFASs and precursors

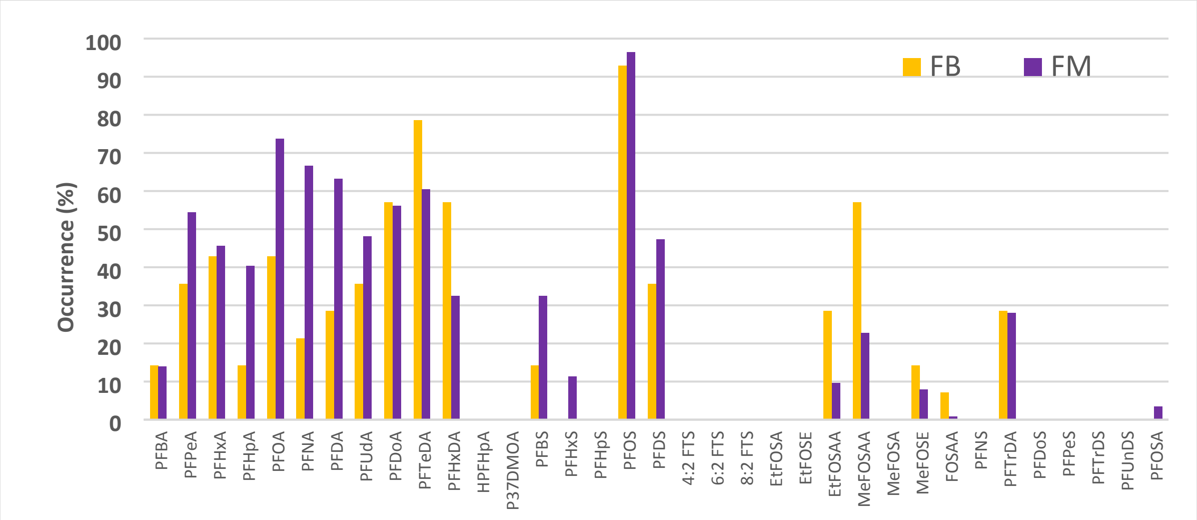

Figure S1. Comparison of PFAS occurrence frequencies in the forebay (FB) and biofilter material (FM)

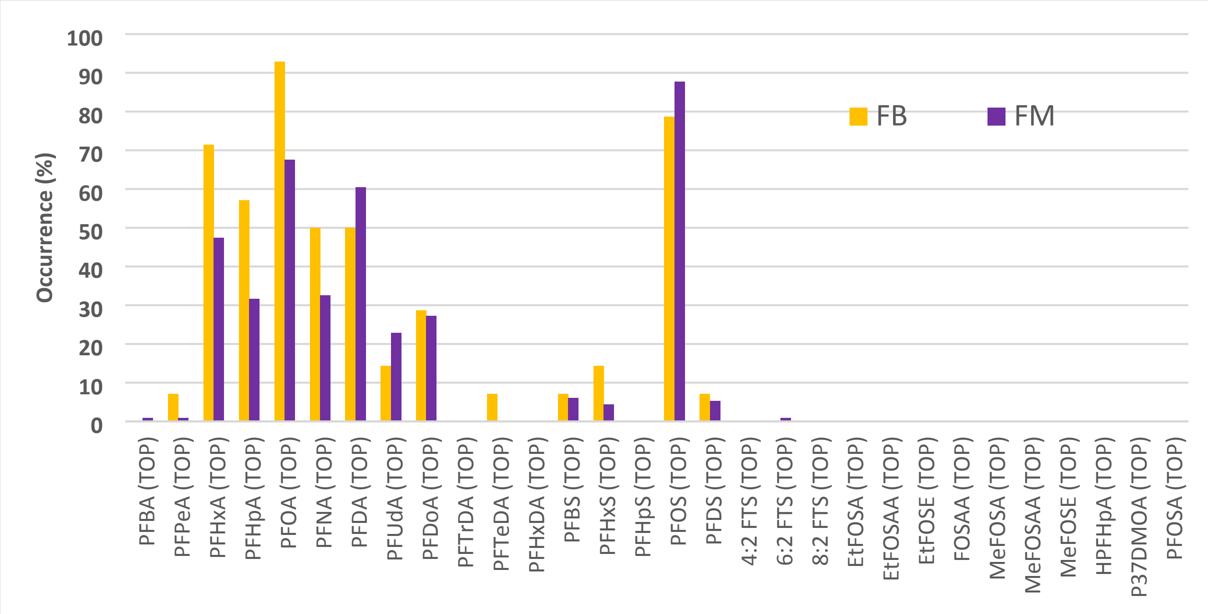

Figure S2. Comparison of PFAS occurrence frequencies in the forebay (FB) and biofilter material (FM) after the oxidation process by TOP assay

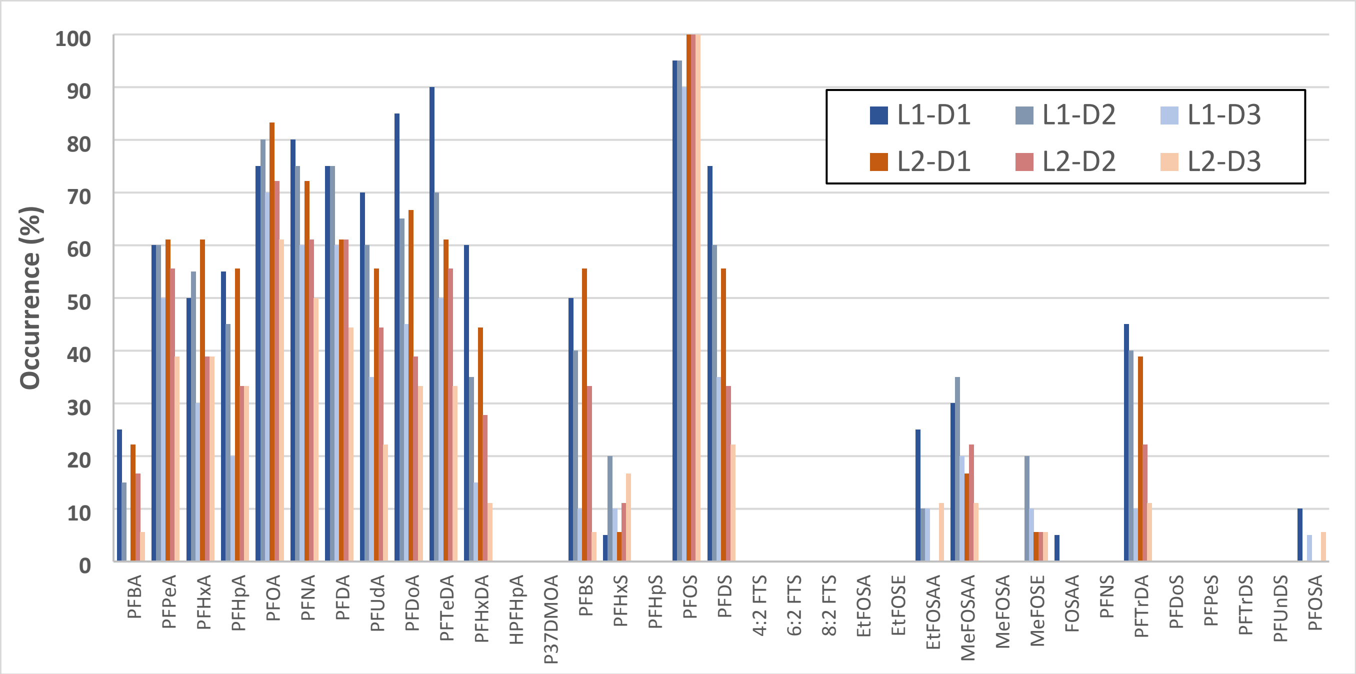

Figure S3. Occurrence frequency of PFASs in different parts of the biofilter media

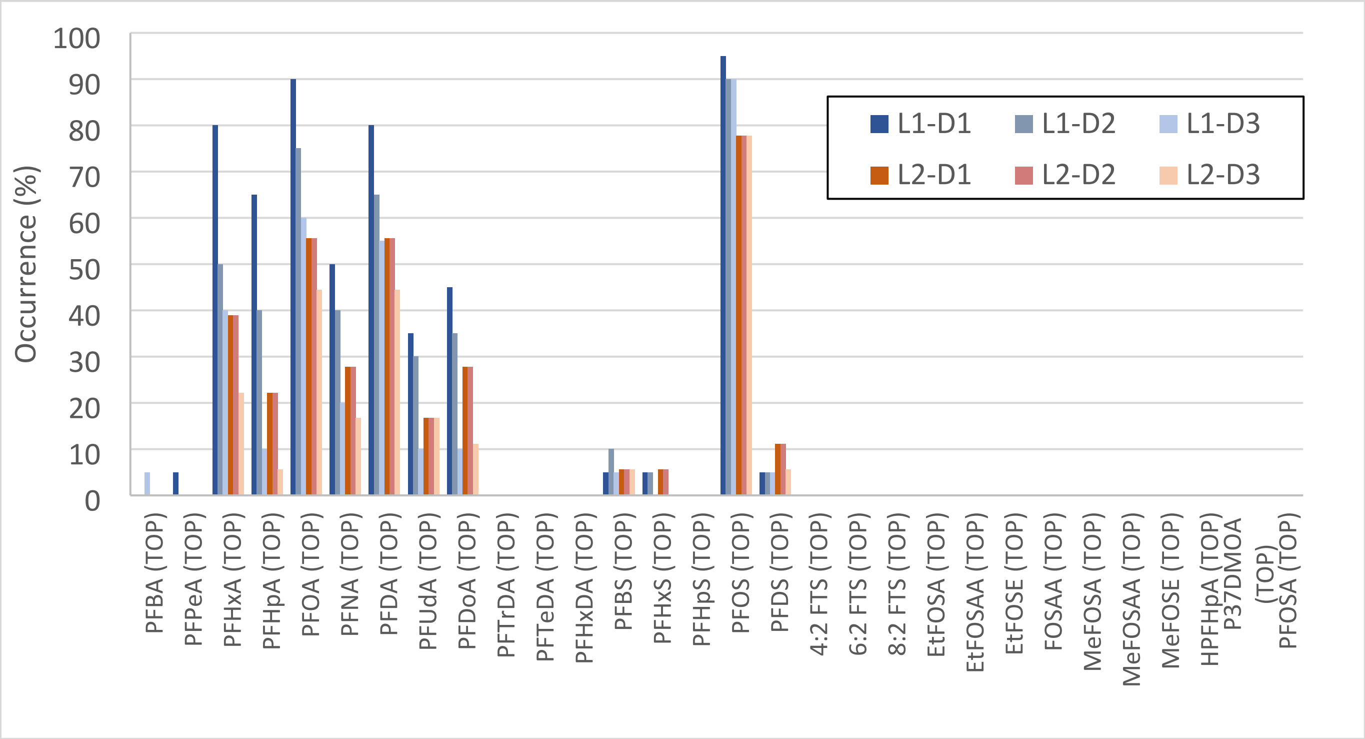

Figure S4. Occurrence frequency of PFASs in different parts of the biofilter media after the oxidation process by TOP assay

Concentration and distribution analysis of PFASs and precursors

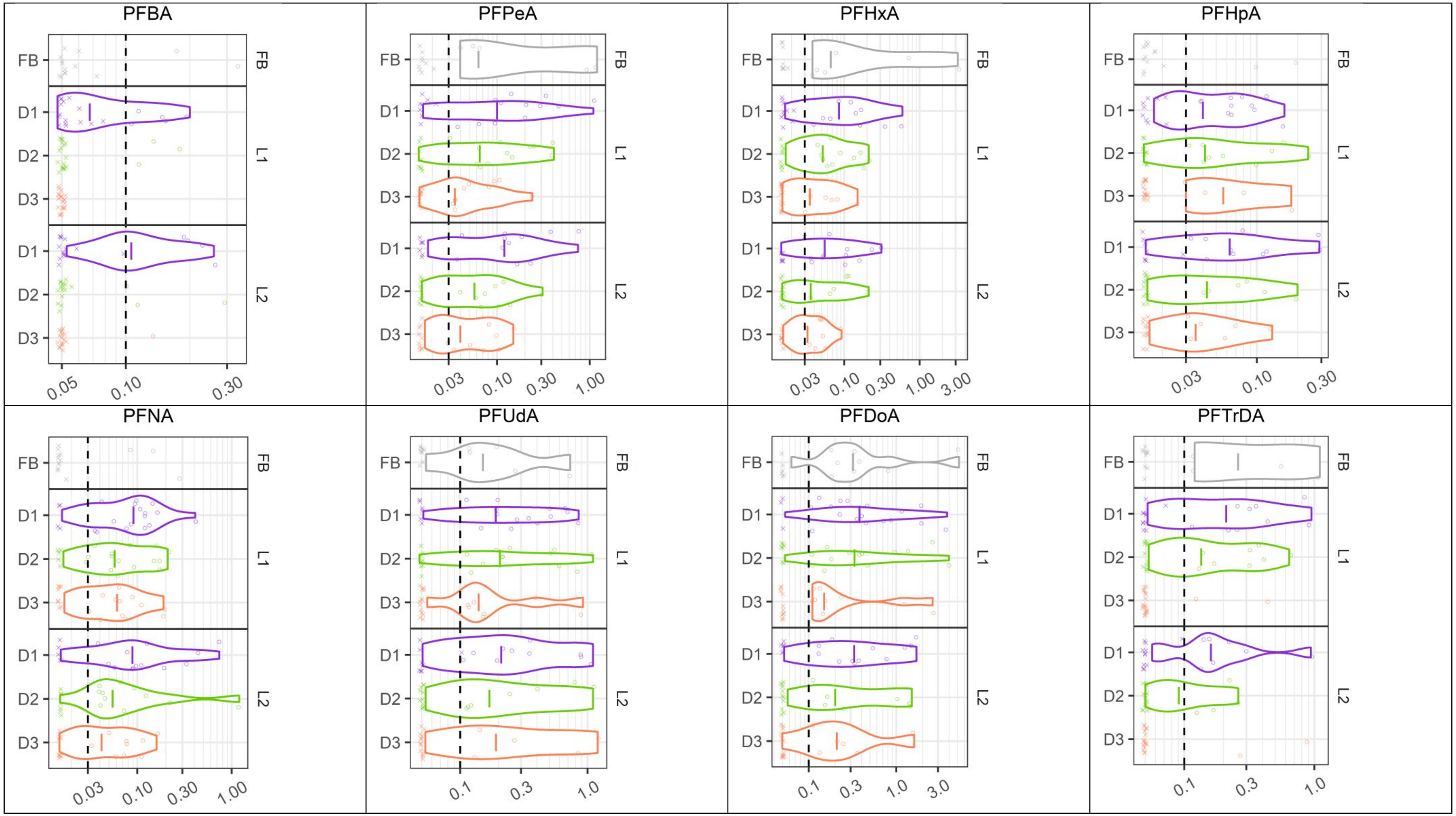

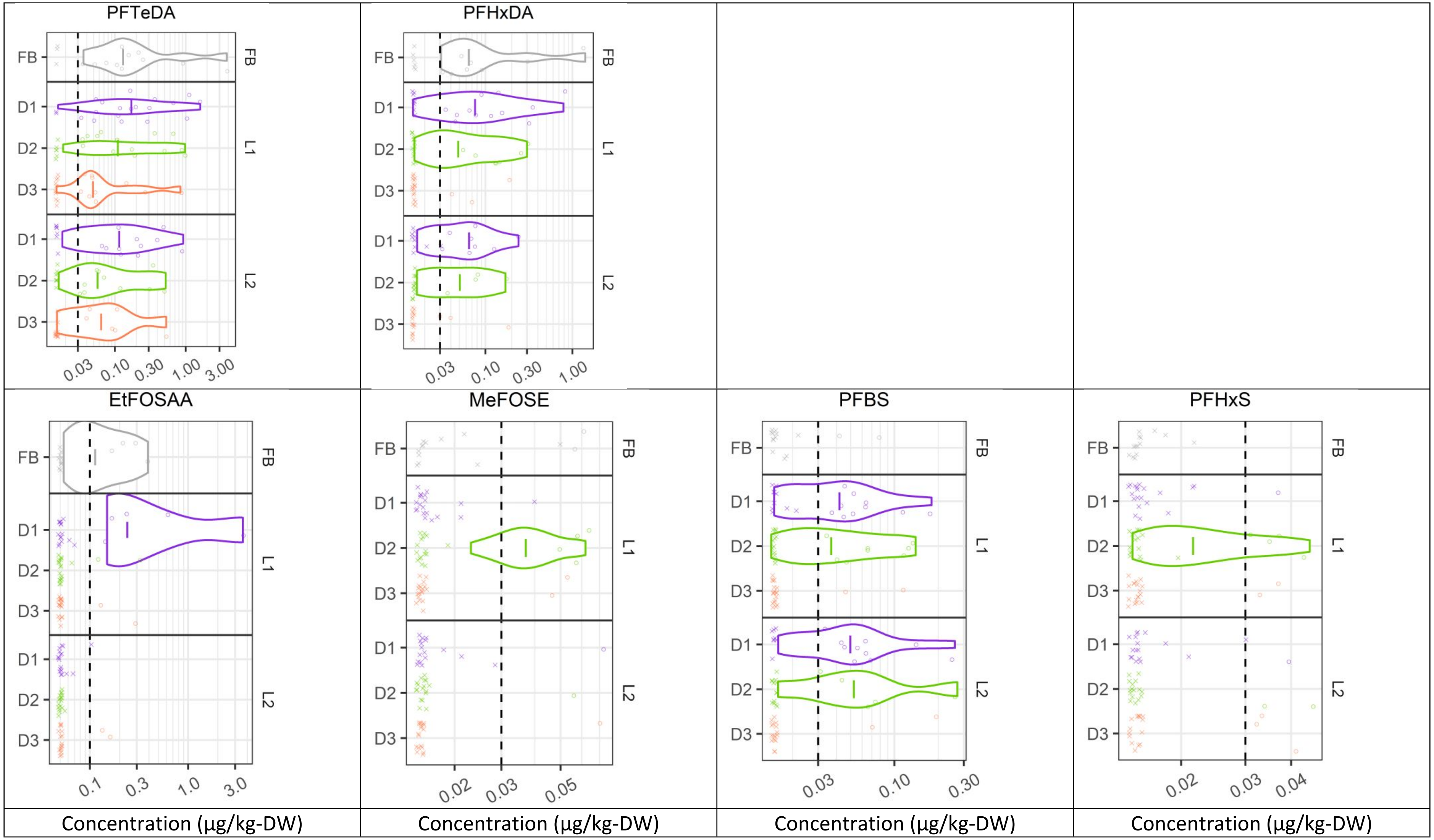

Figure S5. Concentration distribution of other PFASs at forebays (FB) and biofilter materials at different depths (D1–D3) and distances from the inlet (L1 and L2). Cross and circle symbols represent censored and quantified data, respectively. Dashed lines represent LoQ of substances.

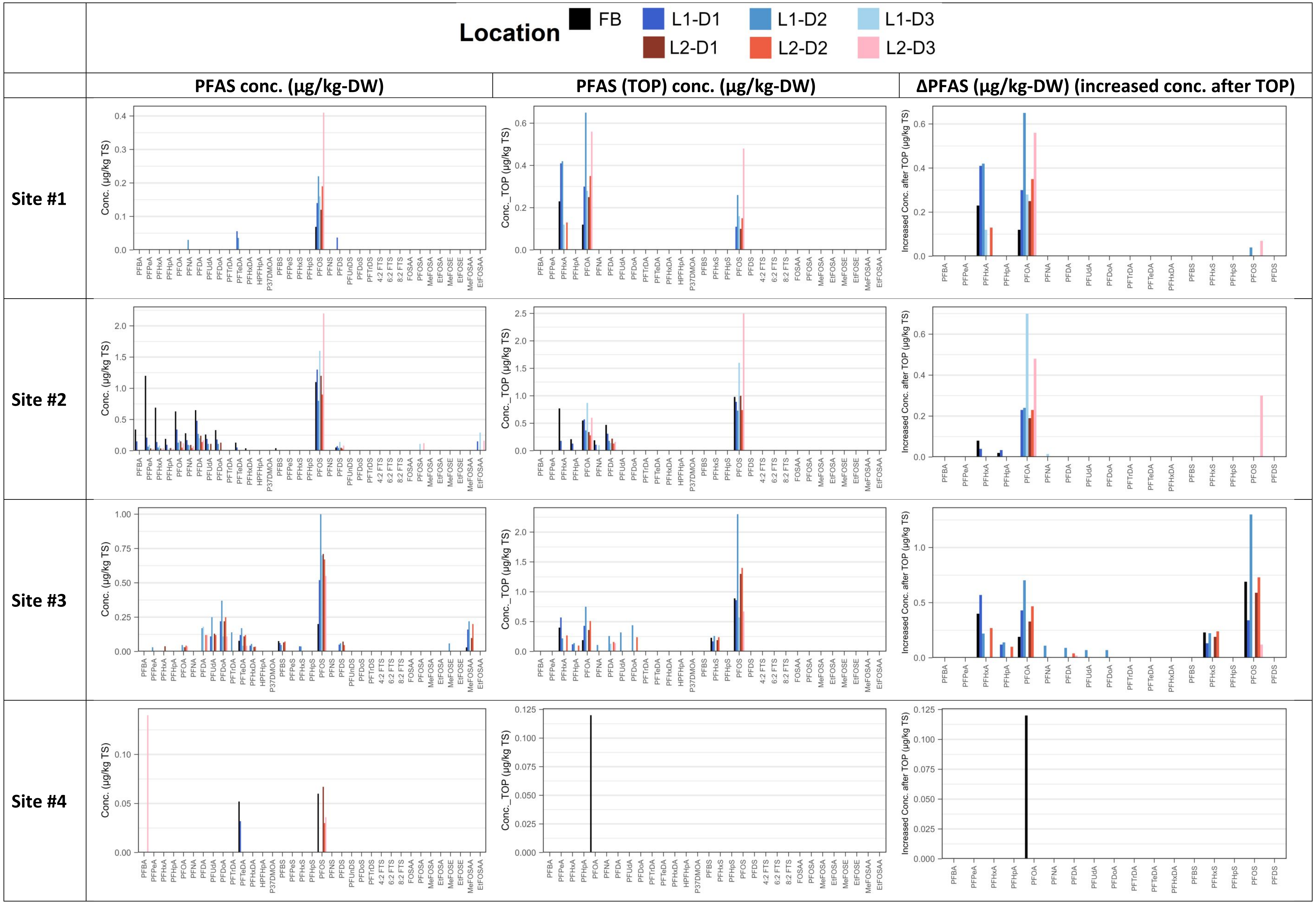
Location
FB
L1-D1
L1-D2
L1-D3
L2-D1
L2-D2
L2-D3
PFAS conc. (µg/kg-DW)
PFAS (TOP) conc. (µg/kg-DW)
ΔPFAS (µg/kg-DW) (increased conc. after TOP)
Site #1
Site #2
Site #3
Site #4
Conc. (µg/kg TS)
Conc._TOP (µg/kg TS)
Increased Conc. after TOP (µg/kg TS)
PFBA
PFPeA
PFHxA
PFHpA
PFOA
PFNA
PFDA
PFUdA
PFDoA
PFTrDA
PFTeDA
PFHxDA
HPFHpA
P37DMOA
PFBS
PFPeS
PFHxS
PFHpS
PFOS
PFNS
PFDS
PFUnDS
PFDoS
PFTrDS
4:2 FTS
6:2 FTS
8:2 FTS
FOSAA
PFOSA
MeFOSA
EtFOSA
MeFOSE
EtFOSE
MeFOSAA
EtFOSAA

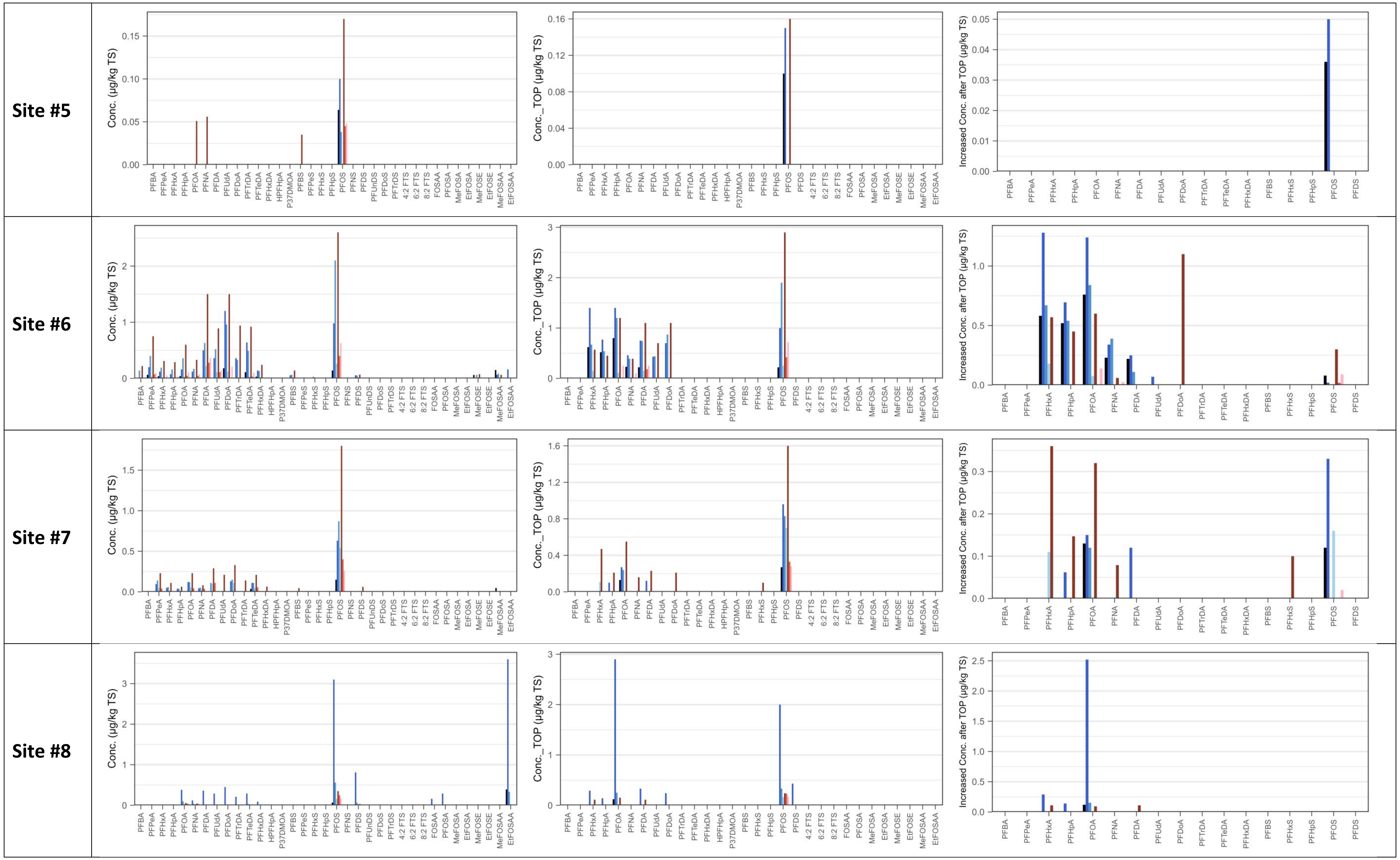
Site #5
Site #6
Site #7
Site #8
Conc. (µg/kg TS)
Conc._TOP (µg/kg TS)
Increased Conc. after TOP (µg/kg TS)
PFBA
PFPeA
PFHxA
PFHpA
PFOA
PFNA
PFDA
PFUdA
PFDoA
PFTrDA
PFTeDA
PFHxDA
HPFHpA
P37DMOA
PFBS
PFPeS
PFHxS
PFHpS
PFOS
PFNS
PFDS
PFUnDS
PFDoS
PFTrDS
4:2 FTS
6:2 FTS
8:2 FTS
FOSAA
PFOSA
MeFOSA
EtFOSA
MeFOSE
EtFOSE
MeFOSAA
EtFOSAA

Site #9

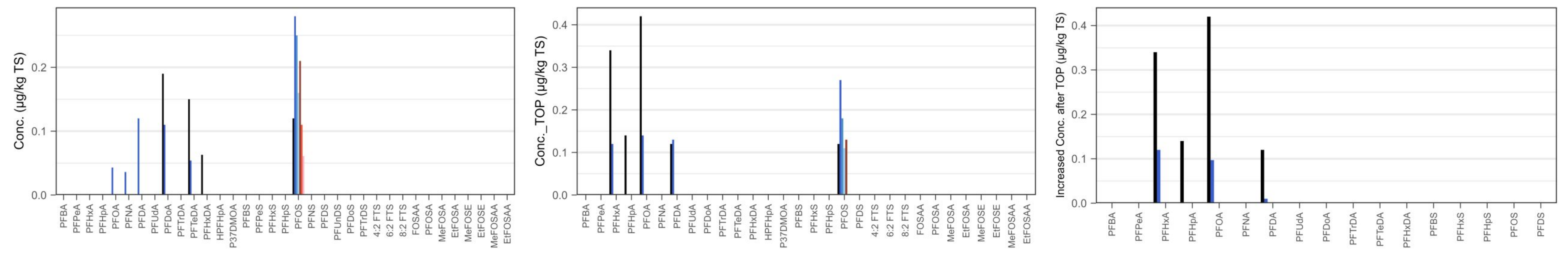

Site #10

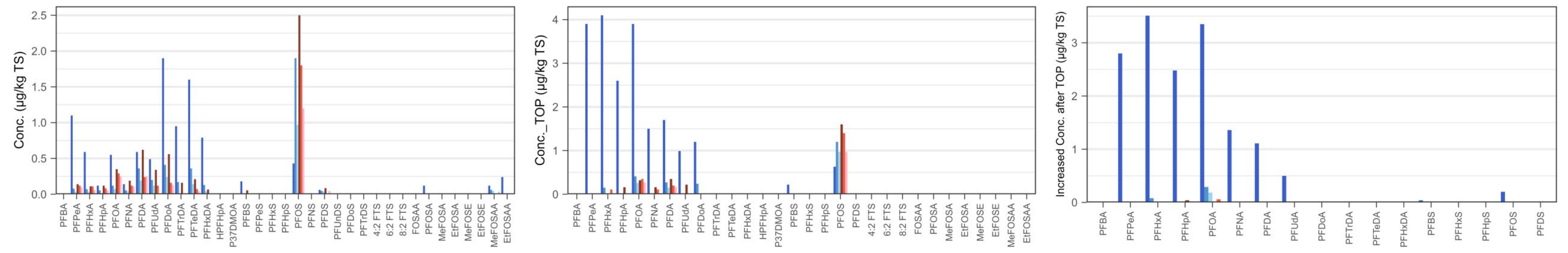

Site #11

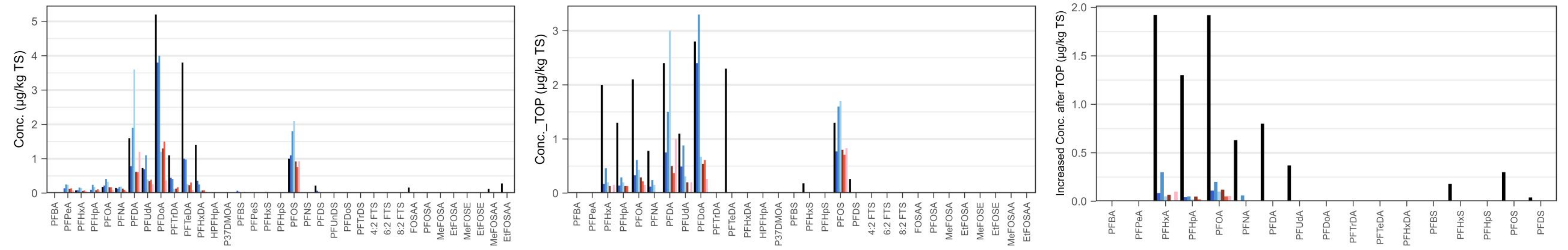

Site #12

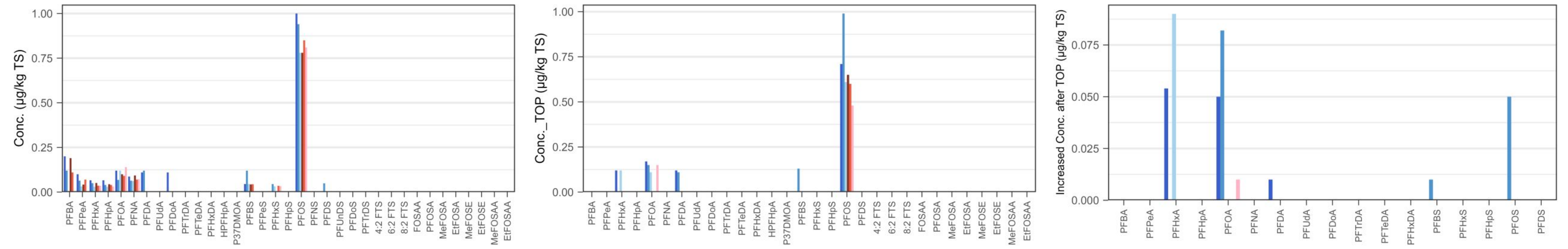

Site #13

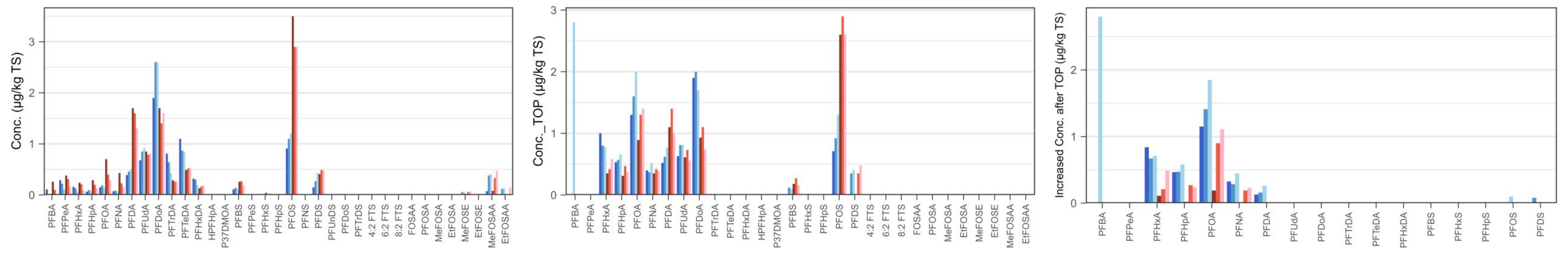

Site #14

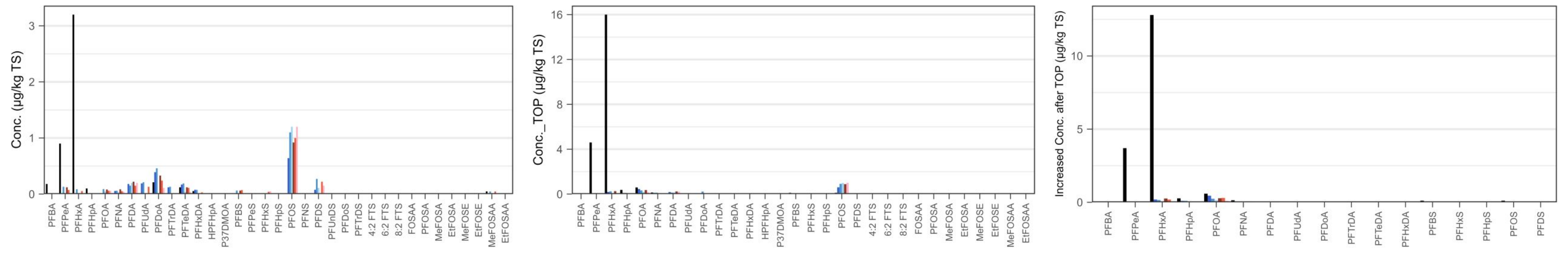

Site #15

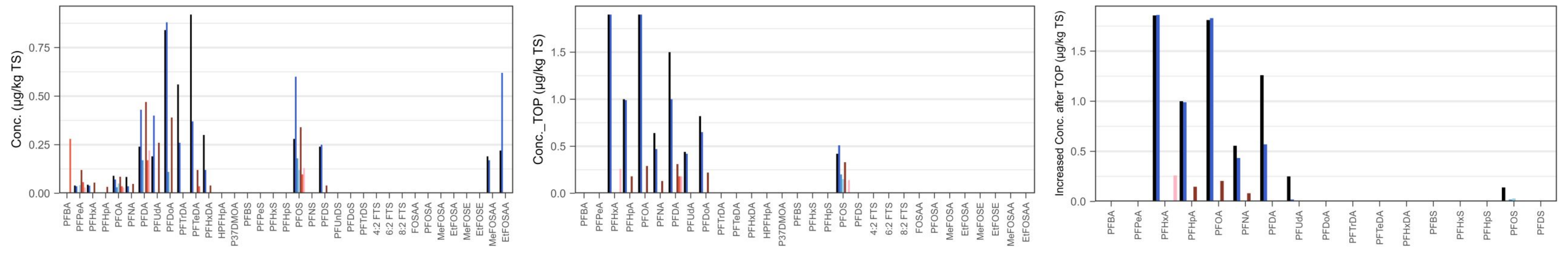

Site #16

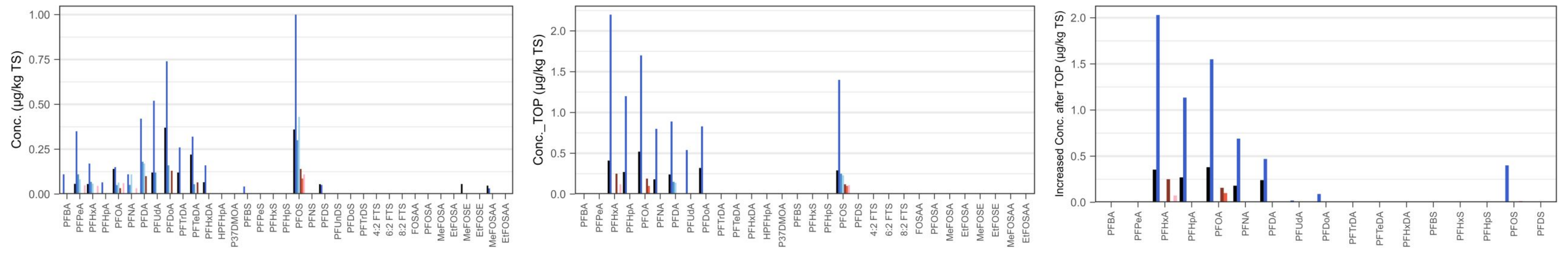

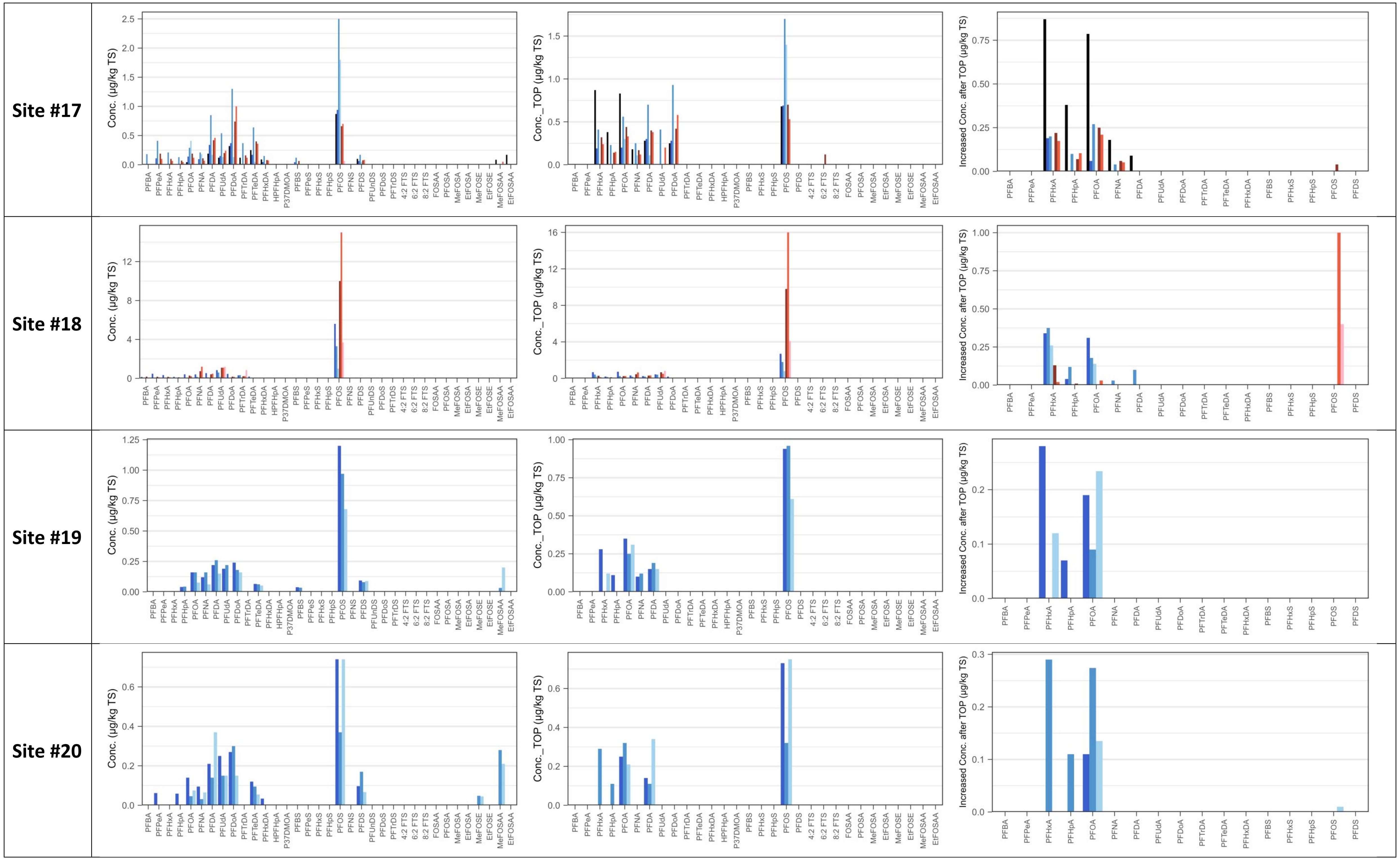

Figure S6. PFAS, PFAS (TOP), and ΔPFAS (increase after TOP) concentrations and accumulation distribution for each site

Table S5. Statistical summary of occurrence frequency (%) and concentration (µg/kg-DW) of 35 targeted PFASs

Parameter	Group	Forebay (FB)					Filter material (FM)					FM at L1-D1					FM at L1-D2					FM at L1-D3					FM at L2-D1					FM at L2-D2					FM at L2-D3				
		%quantified	min	mean	median	max	%quantified	min	mean	median	max	%quantified	min	mean	median	max	%quantified	min	mean	median	max	%quantified	min	mean	median	max	%quantified	min	mean	median	max	%quantified	min	mean	median	max	%quantified	min	mean	median	max
PFBA	SC PFCAs	14.3	<0.1		<0.1	0.34	14	<0.1	0.108	<0.1	0.28	25	<0.1	0.112	<0.105	0.2	15	<0.1		<0.1	0.18	0	<0.1		<0.1	0.1	22.2	<0.1	0.12	<0.1	0.26	17	<0.1	0.111	<0.1	0.28	6	<0.1		<0.1	0.14
PFPeA	SC PFCAs	35.7	<0.03	0.181	0.032	1.2	54	<0.03	0.1	0.035	1.1	60	<0.03	0.171	0.079	1.1	60	<0.03	0.11	0.062	0.41	50	<0.03	0.053	<0.031	0.24	61.1	<0.03	0.139	0.081	0.75	56	<0.03	0.074	0.049	0.31	39	<0.03	0.048	<0.03	0.15
PFHxA	SC PFCAs	42.9	<0.03	0.31	0.032	3.2	46	<0.03	0.062	<0.03	0.59	50	<0.03	0.103	0.043	0.59	55	<0.03	0.063	0.047	0.21	30	<0.03	0.042	<0.03	0.15	61.1	<0.03	0.069	0.053	<0.31	39	<0.03	0.055	<0.03	0.21	39	<0.03	0.039	<0.03	0.092
PFHpA	SC PFCAs	14.3	<0.03		<0.03	0.19	40	<0.03	0.051	<0.03	0.29	55	<0.03	0.058	0.043	0.16	45	<0.03	0.052	<0.03	0.24	20	<0.03	0.04	<0.03	0.18	55.6	<0.03	0.064	0.044	0.29	33	<0.03	0.051	<0.03	0.2	33	<0.03	0.04	<0.03	0.13
PFOA	LC PFCAs	42.9	<0.03	0.097	<0.03	0.63	74	<0.03	0.123	0.066	0.7	75	<0.03	0.166	0.14	0.55	80	<0.03	0.12	0.08	0.41	70	<0.03	0.093	0.053	0.41	83.3	<0.03	0.178	0.093	0.7	72	<0.03	0.098	0.045	0.4	61	<0.03	0.08	0.039	0.29
PFNA	LC PFCAs	21.4	<0.03	0.06	<0.03	0.28	67	<0.03	0.092	0.051	1.2	80	<0.03	0.098	0.091	0.41	75	<0.03	0.073	0.053	0.21	60	<0.03	0.063	0.042	0.19	72.2	<0.03	0.143	0.083	0.74	61	<0.03	0.123	0.042	1.2	50	<0.03	0.056	0.032	0.16
PFDA	LC PFCAs	28.6	<0.1	0.263	<0.1	1.6	63	<0.1	0.321	0.16	3.6	75	<0.1	0.309	0.28	0.78	75	<0.1	0.319	0.16	1.9	60	<0.1	0.343	0.16	3.6	61.1	<0.1	0.406	0.23	1.7	61	<0.1	0.281	0.13	1.6	44	<0.1	0.268	<0.1	1.3
PFUdA	LC PFCAs	35.7	<0.1	0.166	<0.1	0.73	48	<0.1	0.245	<0.1	1.2	70	<0.1	0.298	0.19	0.85	60	<0.1	0.284	0.135	1.1	35	<0.1	0.165	<0.1	0.92	55.6	<0.1	0.291	0.165	1.1	44	<0.1	0.222	<0.1	1.1	22	<0.1	0.212	<0.1	1.2
PFDoA	LC PFCAs	57.1	<0.1	0.589	0.185	5.2	56	<0.1	0.419	0.11	4	85	<0.1	0.683	0.32	3.8	65	<0.1	0.591	0.155	4	45	<0.1	0.296	<0.1	2.6	66.7	<0.1	0.384	0.2	1.7	39	<0.1	0.322	<0.1	1.5	33	<0.1	0.207	<0.1	1.6
PFTrDA	LC PFCAs	28.6	<0.1	0.207	<0.1	1.1	28	<0.1	0.158	<0.1	0.95	45	<0.1	0.242	0.11	0.95	40	<0.1	0.175	<0.1	0.64	10	<0.1	0.118	<0.1	0.43	38.9	<0.1	0.13	<0.1	<0.94	22	<0.1	0.121	<0.1	0.26	11	<0.1	0.152	<0.1	0.86
PFTeDA	LC PFCAs	78.6	<0.03	0.425	0.115	3.8	61	<0.03	0.166	0.046	1.6	90	<0.03	0.326	0.145	1.6	70	<0.03	0.216	0.058	0.98	50	<0.03	0.093	0.032	0.84	61.1	<0.03	0.176	0.093	0.92	56	<0.03	0.106	0.034	0.52	33	<0.03	0.067	<0.03	0.53
PFHxDA	LC PFCAs	57.1	<0.03	0.159	0.035	1.4	33	<0.03	0.061	<0.03	0.79	60	<0.03	0.124	0.044	0.79	35	<0.03	0.069	<0.03	0.3	15	<0.03	0.04	<0.03	0.19	44.4	<0.03	0.047	0.037	<0.24	28	<0.03	0.043	<0.03	0.17	11	<0.03	0.039	<0.03	0.18
HPFHpA	SC PFCAs	0	<0.1		<0.1	<0.15	0	<0.1		<0.1	<0.2	0	<0.1		<0.1	<0.15	0	<0.1		<0.1	<0.13	0	<0.1		<0.1	<0.1	0	<0.1		<0.1	<0.2	0	<0.1		<0.1	<0.11	0	<0.1		<0.1	<0.1
P37DMOA	LC PFCAs	0	<0.5		<0.5	<0.72	0	<0.5		<0.5	<0.99	0	<0.5		<0.5	<0.72	0	<0.5		<0.5	<0.63	0	<0.5		<0.5	<0.5	0	<0.5		<0.5	<0.99	0	<0.5		<0.5	<0.51	0	<0.5		<0.5	<0.5
PFBS	SC PFSAs	14.3	<0.03		<0.03	0.076	33	<0.03	0.046	<0.03	0.27	50	<0.03	0.05	0.04	0.18	40	<0.03	0.048	<0.03	0.14	10	<0.03	0.035	<0.03	0.12	55.6	<0.03	0.054	0.039	0.26	33	<0.03	0.051	<0.03	0.27	6	<0.03		<0.03	0.19
PFPeS	SC PFSAs	0	<0.1		<0.1	<0.15	0	<0.1		<0.1	<0.2	0	<0.1		<0.1	<0.15	0	<0.1		<0.1	<0.13	0	<0.1		<0.1	<0.1	0	<0.1		<0.1	<0.2	0	<0.1		<0.1	<0.11	0	<0.1		<0.1	<0.1
PFHxS	LC PFSAs	0	<0.03		<0.03	<0.043	11	<0.03	0.031	<0.03	<0.059	5	<0.03		<0.03	<0.044	20	<0.03	0.031	<0.03	0.045	10	<0.03		<0.03	0.036	5.6	<0.03		<0.03	<0.059	11	<0.03		<0.03	0.04	17	<0.03	0.031	<0.03	0.048
PFHpS	LC PFSAs	0	<0.03		<0.03	<0.043	0	<0.03		<0.03	<0.059	0	<0.03		<0.03	<0.044	0	<0.03		<0.03	<0.038	0	<0.03		<0.03	<0.03	0	<0.03		<0.03	<0.059	0	<0.03		<0.03	<0.031	0	<0.03		<0.03	<0.03
PFOS	LC PFSAs	92.9	<0.03	0.322	0.145	1.1	97	<0.03	1.095	0.7	15	95	<0.03	1.062	0.825	5.6	95	<0.03	1.016	0.905	3.3	90	<0.03	0.733	0.69	2.1	100	0.067	1.499	0.745	10	100	0.03	1.455	0.535	15	100	0.036	0.857	0.48	3.7
PFNS	LC PFSAs	0	<0.2		<0.2	<0.29	0	<0.2		<0.2	<0.4	0	<0.2		<0.2	<0.29	0	<0.2		<0.2	<0.25	0	<0.2		<0.2	<0.2	0	<0.2		<0.2	<0.4	0	<0.2		<0.2	<0.21	0	<0.2		<0.2	<0.2
PFDS	LC PFSAs	35.7	<0.03	0.067	<0.03	0.24	47	<0.03	0.076	<0.03	0.81	75	<0.03	0.107	0.058	0.81	60	<0.03	0.077	0.046	0.27	35	<0.03	0.066	<0.03	0.43	55.6	<0.03	0.066	0.044	0.41	33	<0.03	0.071	<0.03	0.49	22	<0.03	0.065	<0.03	0.48
PFUnDS	LC PFSAs	0	<1		<1	<1.5	0	<1		<1	<2	0	<1		<1	<1.5	0	<1		<1	<1.3	0	<1		<1	<1	0	<1		<1	<2	0	<1		<1	<1.1	0	<1		<1	<1
PFDoS	LC PFSAs	0	<1		<1	<1.5	0	<1		<1	<2	0	<1		<1	<1.5	0	<1		<1	<1.3	0	<1		<1	<1	0	<1		<1	<2	0	<1		<1	<1.1	0	<1		<1	<1
PFTrDS	LC PFSAs	0	<1		<1	<1.5	0	<1		<1	<2	0	<1		<1	<1.5	0	<1		<1	<1.3	0	<1		<1	<1	0	<1		<1	<2	0	<1		<1	<1.1	0	<1		<1	<1
4:2 FTS	FTSAs	0	<0.03		<0.03	<0.043	0	<0.03		<0.03	<0.059	0	<0.03		<0.03	<0.044	0	<0.03		<0.03	<0.038	0	<0.03		<0.03	<0.03	0	<0.03		<0.03	<0.059	0	<0.03		<0.03	<0.031	0	<0.03		<0.03	<0.03
6:2 FTS	FTSAs	0	<0.03		<0.03	<0.043	0	<0.03		<0.03	<0.059	0	<0.03		<0.03	<0.044	0	<0.03		<0.03	<0.038	0	<0.03		<0.03	<0.03	0	<0.03		<0.03	<0.059	0	<0.03		<0.03	<0.031	0	<0.03		<0.03	<0.03
8:2 FTS	FTSAs	0	<0.1		<0.1	<0.15	0	<0.1		<0.1	<0.2	0	<0.1		<0.1	<0.15	0	<0.1		<0.1	<0.13	0	<0.1		<0.1	<0.1	0	<0.1		<0.1	<0.2	0	<0.1		<0.1	<0.11	0	<0.1		<0.1	<0.1
EtFOSA	PFASAs	0	<0.2		<0.2	<0.29	0	<0.2		<0.2	<0.4	0	<0.2		<0.2	<0.29	0	<0.2		<0.2	<0.25	0	<0.2		<0.2	<0.2	0	<0.2		<0.2	<0.4	0	<0.2		<0.2	<0.21	0	<0.2		<0.2	<0.2
EtFOSE	PFASAs	0	<0.1		<0.1	<0.15	0	<0.1		<0.1	<0.2	0	<0.1		<0.1	<0.15	0	<0.1		<0.1	<0.13	0	<0.1		<0.1	<0.1	0	<0.1		<0.1	<0.2	0	<0.1		<0.1	<0.11	0	<0.1		<0.1	<0.1
EtFOSAA	PFASAs	28.6	<0.1	0.147	<0.1	0.39	10	<0.1	0.142	<0.1	3.6	25	<0.1	0.311	<0.1	3.6	10	<0.1		<0.1	0.33	10	<0.1		<0.1	0.29	0	<0.1		<0.1	<0.2	0	<0.1		<0.1	<0.11	11	<0.1	0.106	<0.1	0.16
MeFOSAA	PFASAs	57.1	<0.03	0.064	0.038	0.19	23	<0.03	0.057	<0.03	0.48	30	<0.03	0.052	0.031	0.17	35	<0.03	0.072	<0.03	0.38	20	<0.03	0.067	<0.03	0.41	16.7	<0.03		<0.03	0.098	22	<0.03	0.058	<0.03	0.33	11	<0.03	0.055	<0.03	0.48
MeFOSA	PFASAs	0	<0.03		<0.03	<0.043	0	<0.03		<0.03	<0.059	0	<0.03		<0.03	<0.044	0	<0.03		<0.03	<0.038	0	<0.03		<0.03	<0.03	0	<0.03		<0.03	<0.059	0	<0.03		<0.03	<0.031	0	<0.03		<0.03	<0.03
MeFOSE	PFASAs	14.3	<0.03		<0.03	<0.1	8	<0.03	0.032	<0.03	<0.08	0	<0.03		<0.03	<0.08	20	<0.03	0.034	<0.03	<0.062	10	<0.03	0.032	<0.03	0.052	5.6	<0.03		<0.03	<0.075	6	<0.03		<0.03	0.057	6	<0.03		<0.03	0.068
FOSAA	PFASAs	7.1	<0.1		<0.1	0.16	1	<0.1		<0.1	<0.2	5	<0.1		<0.1	0.16	0	<0.1		<0.1	<0.13	0	<0.1		<0.1	<0.1	0	<0.1		<0.1	<0.2	0	<0.1		<0.1	<0.11	0	<0.1		<0.1	<0.1
PFOSA	FASAs	0	<0.1		<0.1	<0.15	4	<0.1	0.102	<0.1	0.29	10	<0.1		<0.1	0.29	0	<0.1		<0.1	<0.13	5	<0.1		<0.1	0.11	0	<0.1		<0.1	<0.2	0	<0.1		<0.1	<0.11	6	<0.1		<0.1	0.12
35 PFAS incl. ½ LOQ	Total	100	2.8	5.407	3.3	19	97	<2.8	5.474	4.15	21	100	2.8	6.615	5.45	14	95	<2.8	5.655	4.2	14	90	<2.8	4.455	3.75	11	100	2.8	6.367	4.5	17	100	2.8	5.372	3.7	21	100	2.8	4.344	3.35	12
35 PFAS excl. LOQ	Total	100	0.064	2.696	0.665	16	97	0.03		1.6	19	100	0.032	3.905	2.45	10	95	0.038		1.7	12	90	0.16		1.1	8.9	100	0.067	3.586	1.95	14	100	0.03	2.758	1.095	19	100	0.048	1.767	0.635	9.8

Table S6. Statistical summary of occurrence frequency (%) and concentration (µg/kg-DW) of 31 targeted PFASs after the oxidation process by TOP assay

Parameter	Group	Forebay (FB)					Filter material (FM)					FM at L1-D1					FM at L1-D2				
		%quantified	min	mean	median	max	%quantified	min	mean	median	max	%quantified	min	mean	median	max	%quantified	min	mean	median	max
4:2 FTS (TOP)	FTSAs	0	<0.1		<0.1	<0.12	0	<0.1		<0.1	<0.2	0	<0.1		<0.1	<0.12	0	<0.1		<0.1	<0.2
6:2 FTS (TOP)	FTSAs	0	<0.1		<0.1	<0.12	0.9	<0.1		<0.1	<0.2	0	<0.1		<0.1	<0.12	0	<0.1		<0.1	<0.2
8:2 FTS (TOP)	FTSAs	0	<0.2		<0.2	<0.24	0	<0.2		<0.2	<0.4	0	<0.2		<0.2	<0.24	0	<0.2		<0.2	<0.4
EtFOSA (TOP)	PFASAs	0	<1		<1	<1.2	0	<1		<1	<2	0	<1		<1	<1.2	0	<1		<1	<2
EtFOSAA (TOP)	PFASAs	0	<1		<1	<1.2	0	<1		<1	<2	0	<1		<1	<1.2	0	<1		<1	<2
EtFOSE (TOP)	PFASAs	0	<1		<1	<1.2	0	<1		<1	<2	0	<1		<1	<1.2	0	<1		<1	<2
FOSAA (TOP)	PFASAs	0	<1		<1	<1.2	0	<1		<1	<2	0	<1		<1	<1.2	0	<1		<1	<2
HPFHpA (TOP)	SC PFCAs	0	<0.1		<0.1	<0.12	0	<0.1		<0.1	<0.2	0	<0.1		<0.1	<0.12	0	<0.1		<0.1	<0.2
MeFOSA (TOP)	PFASAs	0	<1		<1	<1.2	0	<1		<1	<2	0	<1		<1	<1.2	0	<1		<1	<2
MeFOSAA (TOP)	PFASAs	0	<1		<1	<1.2	0	<1		<1	<2	0	<1		<1	<1.2	0	<1		<1	<2
MeFOSE (TOP)	PFASAs	0	<1		<1	<1.2	0	<1		<1	<2	0	<1		<1	<1.2	0	<1		<1	<2
P37DMOA (TOP)	LC PFCAs	0	<1		<1	<1.2	0	<1		<1	<2	0	<1		<1	<1.2	0	<1		<1	<2
PFBA (TOP)	SC PFCAs	0	<2		<2	<6	0.9	<2		<2	<4	0	<2		<2	<2.4	0	<2		<2	<4
PFBS (TOP)	SC PFSAs	7.1	<0.1		<0.1	<0.12	6.1	<0.1	0.104	<0.1	0.27	5	<0.1		<0.1	0.22	10	<0.1		<0.1	<0.2
PFDA (TOP)	LC PFCAs	50	<0.1	0.424	0.15	2.4	60.5	<0.1	0.299	0.01	3	80	<0.1	0.402	0.215	1.7	65	<0.1	0.295	0.14	1.5
PFDoA (TOP)	LC PFCAs	28.6	<0.2	0.442	<0.2	2.8	27.2	<0.2	0.373	<0.2	3.3	45	<0.2	0.53	<0.2	2.4	35	<0.2	0.53	<0.2	3.3
PFDS (TOP)	LC PFSAs	7.1	<0.2		<0.2	0.26	5.3	<0.2	0.21	<0.2	0.48	5	<0.2		<0.2	0.43	5	<0.2		<0.2	<0.4
PFHpA (TOP)	SC PFCAs	57.1	<0.1	0.342	<0.1	1.3	31.6	<0.1	0.183	0.175	2.6	65	<0.1	0.392	0.115	2.6	40	<0.1	0.167	<0.1	0.57
PFHpS (TOP)	LC PFSAs	0	<0.1		<0.1	<0.12	0	<0.1		<0.1	<0.2	0	<0.1		<0.1	<0.12	0	<0.1		<0.1	<0.2
PFHxA (TOP)	SC PFCAs	71.4	<0.1	1.71	0.11	16	47.4	<0.1	0.273	0.405	4.1	80	<0.1	0.71	0.235	4.1	50	<0.1	0.254	0.025	0.8
PFHxDA (TOP)	LC PFCAs	0	<1		<1	<1.2	0	<1		<1	<2	0	<1		<1	<1.2	0	<1		<1	<2
PFHxS (TOP)	LC PFSAs	14.3	<0.1		<0.1	0.23	4.4	<0.1	0.104	<0.1	0.26	5	<0.1		<0.1	0.17	5	<0.1		<0.1	0.26
PFNA (TOP)	LC PFCAs	50	<0.1	0.217	<0.1	0.78	32.5	<0.1	0.163	0.02	1.5	50	<0.1	0.27	<0.1	1.5	40	<0.1	0.15	<0.1	0.39
PFOA (TOP)	LC PFCAs	92.9	<0.1	0.606	0.25	2.1	67.5	<0.1	0.418	0.47	3.9	90	<0.1	0.875	0.39	3.9	75	<0.1	0.423	0.285	1.6
PFOS (TOP)	LC PFSAs	78.6	<0.1	0.406	0.695	1.3	87.7	<0.1	1.008	0.245	16	95	<0.1	0.837	0.72	2.7	90	<0.1	0.879	0.87	2.3
PFOSA (TOP)	FASAs	0	<0.1		<0.1	<0.12	0	<0.1		<0.1	<0.2	0	<0.1		<0.1	<0.12	0	<0.1		<0.1	<0.2
PFPeA (TOP)	LC PFCAs	7.1	<2		<2	4.6	0.9	<2		<2	<4	5	<2		<2	3.9	0	<2		<2	<4
PFTeDA (TOP)	LC PFCAs	7.1	<1		<1	2.3	0	<1		<1	<2	0	<1		<1	<1.2	0	<1		<1	<2
PFTrDA (TOP)	LC PFCAs	0	<1		<1	<1.2	0	<1		<1	<2	0	<1		<1	<1.2	0	<1		<1	<2
PFUdA (TOP)	LC PFCAs	14.3	<0.2		<0.2	1.1	22.8	<0.2	0.275	<0.2	0.99	35	<0.2	0.327	<0.2	0.99	30	<0.2	0.304	<0.2	0.88
31 PFAS (TOP) excl. LOQ	Total	100	0.1	4.463	1.38	21.92	88.6	0.11		1.97	20.74	95	0.15		2.15	20.74	90	0.18		1.45	8.88
		100	0.064	2.696	0.665	16	97	0.03		1.6	19	100	0.032	3.905	2.45	10	95	0.038		1.7	12

Parameter	Group	FM at L1-D3					FM at L2-D1					FM at L2-D2					FM at L2-D3				
		%quantified	min	mean	median	max	%quantified	min	mean	median	max	%quantified	min	mean	median	max	%quantified	min	mean	median	max
4:2 FTS (TOP)	FTSAs	0	<0.1		<0.1	<0.2	0	<0.1		<0.1	<0.2	0	<0.1		<0.1	<0.1	0	<0.1		<0.1	<0.2
6:2 FTS (TOP)	FTSAs	0	<0.1		<0.1	<0.2	5.6	<0.1		<0.1	<0.2	0	<0.1		<0.1	<0.1	0	<0.1		<0.1	<0.2
8:2 FTS (TOP)	FTSAs	0	<0.2		<0.2	<0.4	0	<0.2		<0.2	<0.4	0	<0.2		<0.2	<0.2	0	<0.2		<0.2	<0.4
EtFOSA (TOP)	PFASAs	0	<1		<1	<2	0	<1		<1	<2	0	<1		<1	<1	0	<1		<1	<2
EtFOSAA (TOP)	PFASAs	0	<1		<1	<2	0	<1		<1	<2	0	<1		<1	<1	0	<1		<1	<2
EtFOSE (TOP)	PFASAs	0	<1		<1	<2	0	<1		<1	<2	0	<1		<1	<1	0	<1		<1	<2
FOSAA (TOP)	PFASAs	0	<1		<1	<2	0	<1		<1	<2	0	<1		<1	<1	0	<1		<1	<2
HPFHpA (TOP)	SC PFCAs	0	<0.1		<0.1	<0.2	0	<0.1		<0.1	<0.2	0	<0.1		<0.1	<0.1	0	<0.1		<0.1	<0.2
MeFOSA (TOP)	PFASAs	0	<1		<1	<2	0	<1		<1	<2	0	<1		<1	<1	0	<1		<1	<2
MeFOSAA (TOP)	PFASAs	0	<1		<1	<2	0	<1		<1	<2	0	<1		<1	<1	0	<1		<1	<2
MeFOSE (TOP)	PFASAs	0	<1		<1	<2	0	<1		<1	<2	0	<1		<1	<1	0	<1		<1	<2
P37DMOA (TOP)	LC PFCAs	0	<1		<1	<2	0	<1		<1	<2	0	<1		<1	<1	0	<1		<1	<2
PFBA (TOP)	SC PFCAs	5	<2		<2	<4	0	<2		<2	<4	0	<2		<2	<2	0	<2		<2	<4
PFBS (TOP)	SC PFSAs	5	<0.1		<0.1	<0.2	5.6	<0.1		<0.1	<0.2	5.6	<0.1		<0.1	0.27	5.6	<0.1		<0.1	<0.2
PFDA (TOP)	LC PFCAs	55	<0.1	0.308	0.12	3	61.1	<0.1	0.309	0.225	1.1	55.6	<0.1	0.239	0.145	1.4	44.4	<0.1	0.229	<0.1	1
PFDoA (TOP)	LC PFCAs	10	<0.2		<0.2	1.7	33.3	<0.2	0.324	<0.2	1.1	27.8	<0.2	0.297	<0.2	1.1	11.1	<0.2		<0.2	0.74
PFDS (TOP)	LC PFSAs	5	<0.2		<0.2	0.41	0	<0.2		<0.2	<0.4	11.1	<0.2		<0.2	0.35	5.6	<0.2		<0.2	0.48
PFHpA (TOP)	SC PFCAs	10	<0.1		<0.1	0.66	44.4	<0.1	0.152	<0.1	0.45	22.2	<0.1	0.125	<0.1	0.47	5.6	<0.1		<0.1	0.37
PFHpS (TOP)	LC PFSAs	0	<0.1		<0.1	<0.2	0	<0.1		<0.1	<0.2	0	<0.1		<0.1	<0.1	0	<0.1		<0.1	<0.2
PFHxA (TOP)	SC PFCAs	40	<0.1	0.153	<0.1	0.78	50	<0.1	0.203	0.01	0.57	38.9	<0.1	0.147	<0.1	0.42	22.2	<0.1	0.14	<0.1	0.58
PFHxDA (TOP)	LC PFCAs	0	<1		<1	<2	0	<1		<1	<2	0	<1		<1	<1	0	<1		<1	<2
PFHxS (TOP)	LC PFSAs	0	<0.1		<0.1	<0.2	11.1	<0.1		<0.1	<0.2	5.6	<0.1		<0.1	0.24	0	<0.1		<0.1	<0.2
PFNA (TOP)	LC PFCAs	20	<0.1	0.124	<0.1	0.52	38.9	<0.1	0.163	<0.1	0.45	27.8	<0.1	0.15	<0.1	0.65	16.7	<0.1	0.117	<0.1	0.39
PFOA (TOP)	LC PFCAs	60	<0.1	0.30	0.125	2	77.8	<0.1	0.349	0.29	1.2	55.6	<0.1	0.269	0.16	1.3	44.4	<0.1	0.252	<0.1	1.4
PFOS (TOP)	LC PFSAs	90	<0.1	0.66	0.61	1.7	94.4	<0.1	1.389	0.75	9.8	77.8	<0.1	1.492	0.475	16	77.8	<0.1	0.858	0.48	4.1
PFOSA (TOP)	FASAs	0	<0.1		<0.1	<0.2	0	<0.1		<0.1	<0.2	0	<0.1		<0.1	<0.1	0	<0.1		<0.1	<0.2
PFPeA (TOP)	LC PFCAs	0	<2		<2	<4	0	<2		<2	<4	0	<2		<2	<2	0	<2		<2	<4
PFTeDA (TOP)	LC PFCAs	0	<1		<1	<2	0	<1		<1	<2	0	<1		<1	<1	0	<1		<1	<2
PFTrDA (TOP)	LC PFCAs	0	<1		<1	<2	0	<1		<1	<2	0	<1		<1	<1	0	<1		<1	<2
PFUdA (TOP)	LC PFCAs	10	<0.2		<0.2	0.81	27.8	<0.2	0.278	<0.2	0.7	16.7	<0.2	0.246	<0.2	0.73	16.7	<0.2	0.254	<0.2	0.82
31 PFAS (TOP) excl. LOQ	Total	90	0.11		1.015	11.85	94.4	0.13		1.73	11.89	83.3	0.18		1.15	17.85	77.8	0.17		1.18	8.28
		90	0.16		1.1	8.9	100	0.067	3.586	1.95	14	100	0.03	2.758	1.095	19	100	0.048	1.767	0.635	9.8

Table S7. P-values of the significance of difference tests (acceptancy p-value: 0.05) between different sampling points for the concentrations of the most frequently found PFASs, PFAAs after TOP assay, and the estimated total precursors (Pre) (Peto&Peto modification of Wilcoxon test in "cendiff" function in the NADA package was used for the series including censored data, and "Wilcoxon test" otherwise)

parameter	L1D1 vs L1D2	L1D1 vs L1D3	L1D2 vs L1D3	L2D1 vs L2D2	L2D1 vs L2D3	L2D2 vs L2D3	D1 vs D2	D1 vs D3	D2 vs D3	L1 vs L2
PFPeA	0.678	0.061	0.158	0.277	0.038	0.217	0.319	0.005	0.055	0.557
PFHxA	0.761	0.095	0.158	0.322	0.069	0.48	0.373	0.015	0.122	0.808
PFHpA	0.264	0.026	0.214	0.366	0.12	0.594	0.147	0.006	0.213	0.93
PFOA	0.337	0.089	0.307	0.165	0.067	0.541	0.111	0.009	0.263	0.407
PFNA	0.276	0.073	0.649	0.19	0.059	0.62	0.084	0.009	0.48	0.464
PFDA	0.421	0.075	0.484	0.515	0.164	0.463	0.318	0.027	0.308	0.391
PFUdA	0.572	0.008	0.058	0.337	0.063	0.292	0.251	0.002	0.045	0.208
PFDoA	0.219	0.002	0.087	0.244	0.029	0.408	0.083	0	0.067	0.064
PFTrDA	0.48	0.022	0.1	0.416	0.229	0.749	0.283	0.012	0.144	0.256
PFTeDA	0.138	0	0.069	0.316	0.026	0.158	0.073	0	0.022	0.049
PFHxDA	0.111	0.005	0.31	0.383	0.084	0.42	0.073	0.001	0.197	0.176
PFBS	0.456	0.015	0.106	0.39	0.009	0.107	0.24	0	0.024	0.889
PFOS	0.914	0.517	0.304	0.327	0.275	0.987	0.504	0.194	0.541	0.367
PFDS	0.242	0.04	0.304	0.272	0.176	0.699	0.101	0.015	0.324	0.04
MeFOSAA	0.924	0.78	0.632	0.409	0.765	0.606	0.581	0.914	0.497	0.134
Sum 35 PFAS inkl ½ LOQ	0.317	0.015	0.146	0.223	0.096	0.668	0.122	0.003	0.21	0.325
Sum 35 PFAS exkl. LOQ	0.509	0.05	0.157	0.275	0.103	0.704	0.235	0.014	0.249	0.115
PFHxA (TOP)	0.095	0.001	0.157	0.257	0.077	0.357	0.048	0	0.112	0.017
PFHpA (TOP)	0.173	0.001	0.041	0.093	0.01	0.184	0.034	0	0.016	0.088
PFOA (TOP)	0.163	0.003	0.085	0.197	0.038	0.467	0.076	0	0.084	0.041
PFNA (TOP)	0.426	0.031	0.112	0.313	0.08	0.402	0.217	0.005	0.087	0.278
PFDA (TOP)	0.252	0.056	0.261	0.374	0.149	0.535	0.173	0.021	0.2	0.437
PFUdA (TOP)	0.603	0.06	0.1	0.391	0.409	0.941	0.406	0.056	0.244	0.512
PFDoA (TOP)	0.659	0.02	0.065	0.698	0.101	0.248	0.5	0.005	0.03	0.336
PFOS (TOP)	0.766	0.4	0.246	0.304	0.312	1	0.533	0.176	0.541	0.35
31 PFAS (TOP) inkl. ½ LOQ	0.627	0.017	0.087	0.152	0.183	0.975	0.186	0.009	0.25	0.162

**31 PFAS (TOP) exkl. LOQ**	0.281	0.012	0.107	0.597	0.404	1	0.266	0.014	0.232	0.421
**ΔPFBA**	0.61	0.13	0.33	0.70	0.50	1.00	0.84	0.10	0.25	0.14
**ΔPFPeA**	0.93	0.04	0.05	0.10	0.03	0.41	0.26	0.00	0.03	0.87
**ΔPFHxA**	0.23	0.03	0.30	0.56	0.39	1.00	0.19	0.04	0.37	0.04
**ΔPFHpA**	0.94	0.63	0.96	1.00	0.87	1.00	0.95	0.69	0.73	0.07
**ΔPFOA**	0.29	0.04	0.13	0.37	0.19	0.98	0.14	0.01	0.27	0.03
**ΔPFNA**	0.45	0.53	0.56	1.00	0.79	0.83	0.65	0.71	0.47	0.27
**ΔPFDA**	0.60	0.26	0.59	1.00	1.00	0.54	0.58	0.22	0.17	0.08
**ΔPFUdA**	0.64	0.25	1.00	0.59	1.00	0.28	0.27	0.35	0.51	0.34
**ΔPFDoA**	1.00	0.87	0.62	0.86	0.58	0.66	0.93	0.53	0.37	0.53
**ΔPFTrDA**	0.67	0.23	0.38	0.75	0.70	0.63	0.82	0.48	0.69	0.13
**ΔPFTeDA**	1.00	0.06	0.16	0.14	0.06	0.66	0.35	0.01	0.13	0.86
**ΔPFHxDA**	0.75	0.26	0.17	1.00	0.83	1.00	0.81	0.25	0.24	0.06
**ΔPFBS**	1.00	0.24	0.38	0.33	0.17	0.29	0.47	0.04	0.06	0.40
**ΔPFHxS**	0.80	0.33	1.00	1.00	0.40	1.00	0.79	0.09	1.00	0.79
**ΔPFOS**	0.58	0.02	0.12	0.57	0.47	0.73	0.35	0.02	0.51	0.70
**ΔPFDS**	0.93	0.91	0.73	1.00	0.52	0.69	0.87	0.71	0.90	0.59

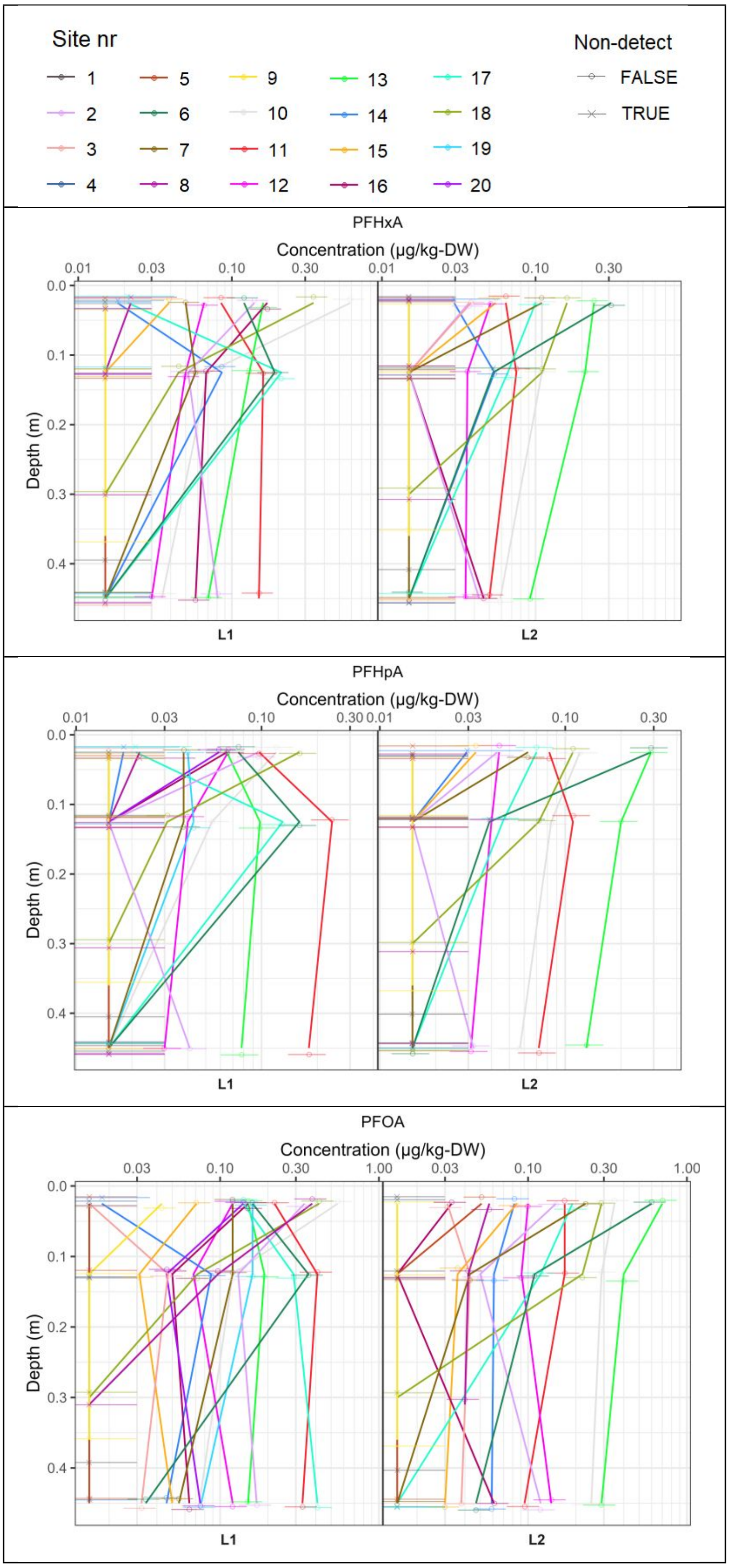

Site nr
1
2
3
4
5
6
7
8
9
10
11
12
13
14
15
16
17
18
19
20
Non-detect
FALSE
TRUE
PFHxA
Concentration (µg/kg-DW)
Depth (m)
L1
L2
PFHpA
Concentration (µg/kg-DW)
Depth (m)
L1
L2
PFOA
Concentration (µg/kg-DW)
Depth (m)
L1
L2

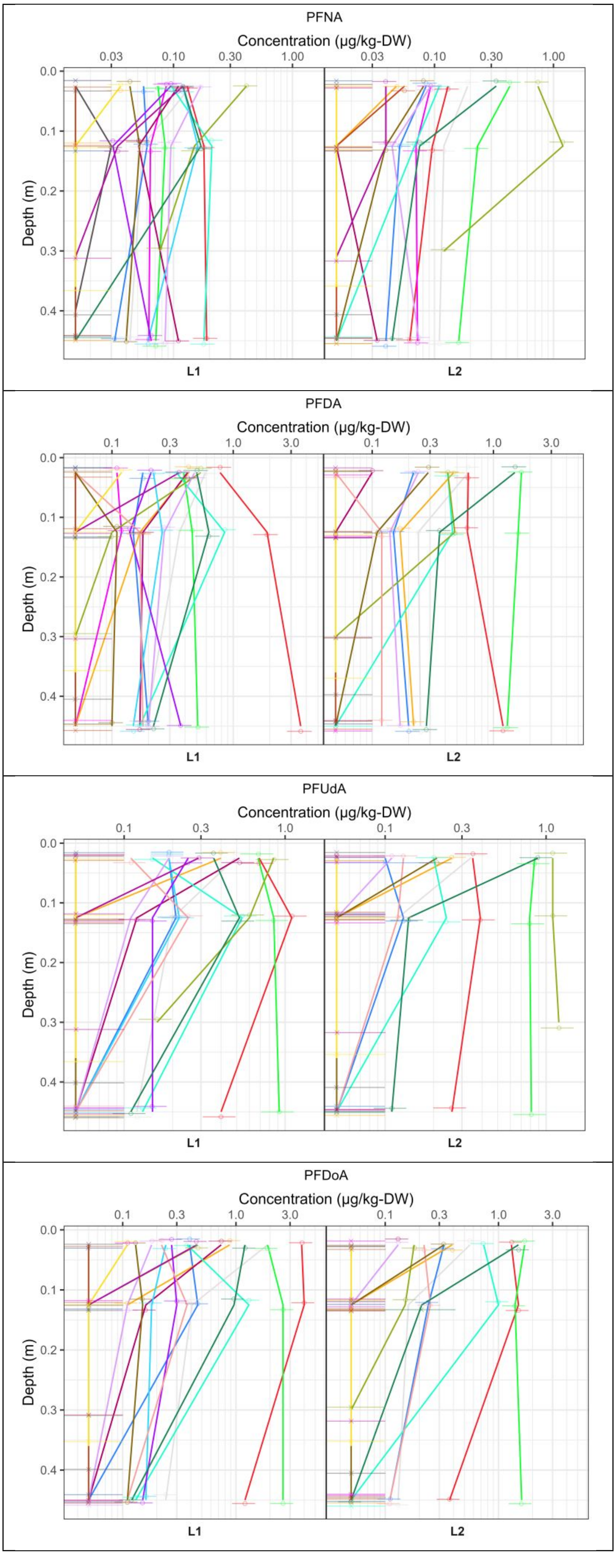

PFNA
Concentration (µg/kg-DW)
0.03 0.10 0.30 1.00
Depth (m)
0.0 0.1 0.2 0.3 0.4
L1
L2
PFDA
Concentration (µg/kg-DW)
0.1 0.3 1.0 3.0
Depth (m)
L1
L2
PFUdA
Concentration (µg/kg-DW)
0.1 0.3 1.0
Depth (m)
L1
L2
PFDoA
Concentration (µg/kg-DW)
0.1 0.3 1.0 3.0
Depth (m)
L1
L2

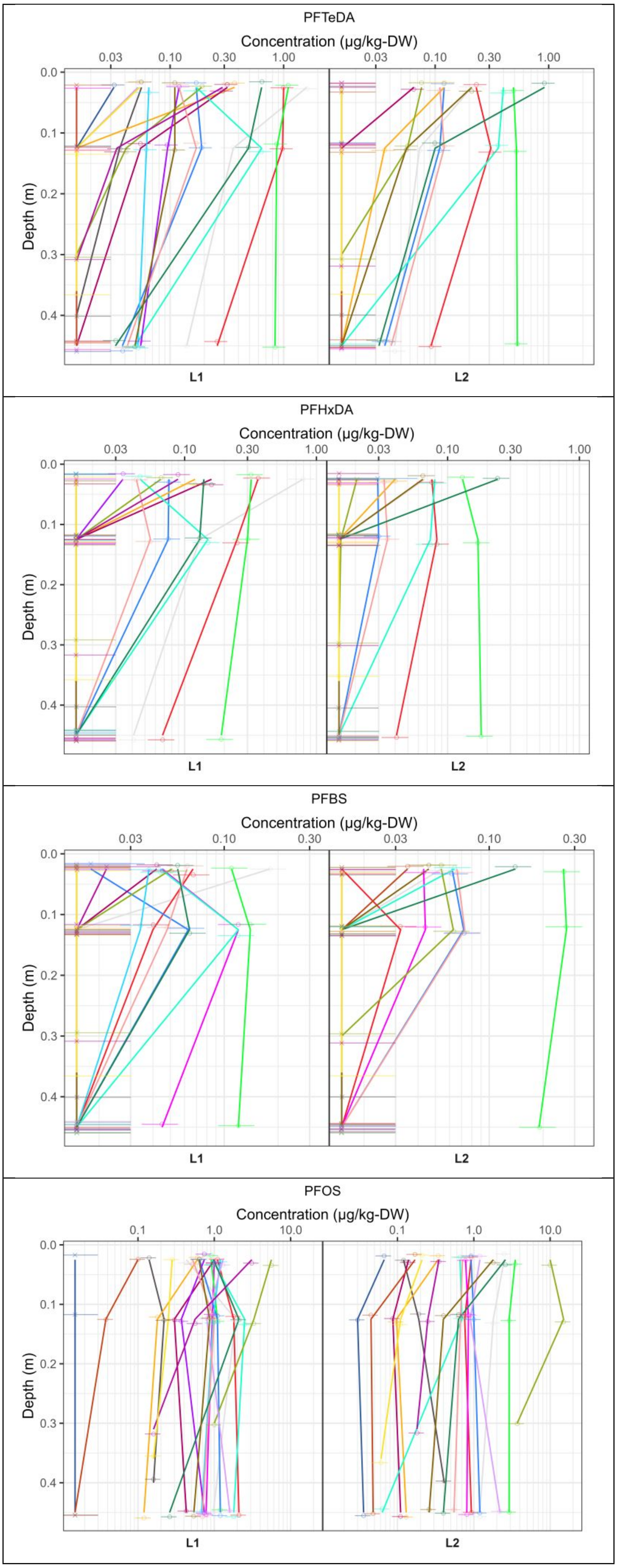
PFTeDA
Concentration (µg/kg-DW)
0.03
0.10
0.30
1.00
Depth (m)
0.0
0.1
0.2
0.3
0.4
L1
L2
PFHxDA
Concentration (µg/kg-DW)
L1
L2
PFBS
Concentration (µg/kg-DW)
L1
L2
PFOS
Concentration (µg/kg-DW)
0.1
1.0
10.0
L1
L2

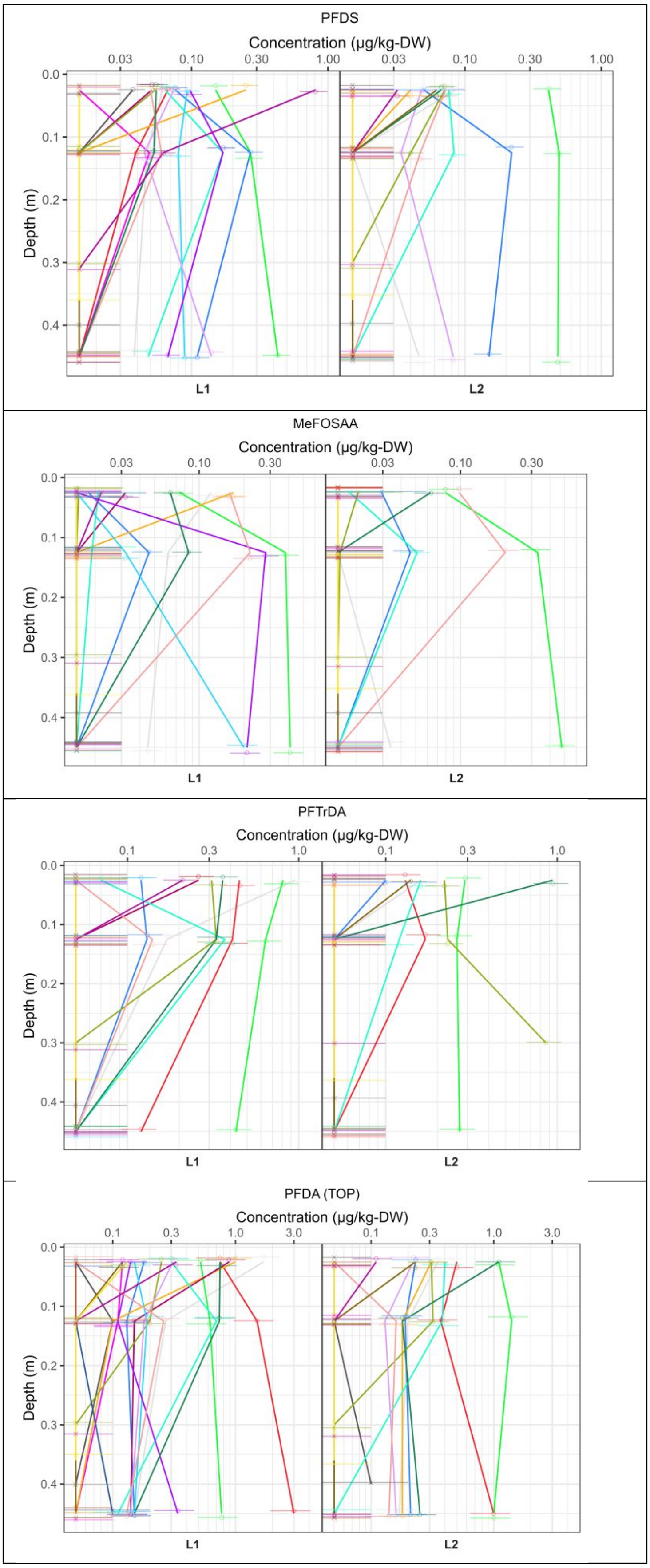
PFDS
Concentration (µg/kg-DW)
0.03
0.10
0.30
1.00
Depth (m)
0.0
0.1
0.2
0.3
0.4
L1
L2
MeFOSAA
Concentration (µg/kg-DW)
0.03
0.10
0.30
Depth (m)
L1
L2
PFTrDA
Concentration (µg/kg-DW)
0.1
0.3
1.0
Depth (m)
L1
L2
PFDA (TOP)
Concentration (µg/kg-DW)
0.1
0.3
1.0
3.0
Depth (m)
L1
L2

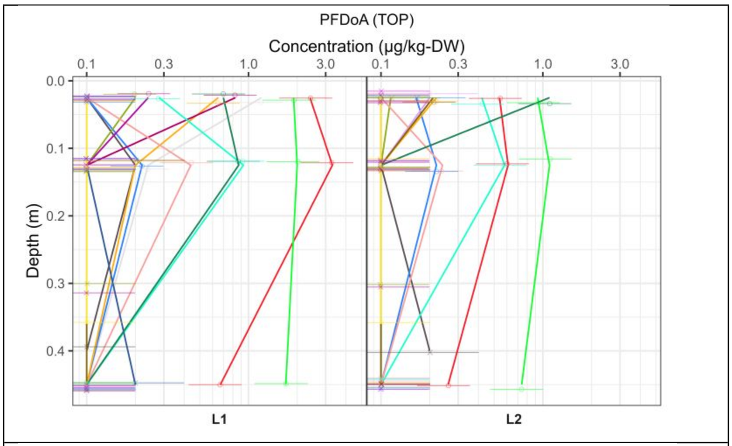
PFDoA (TOP)
Concentration (µg/kg-DW)
0.1
0.3
1.0
3.0
Depth (m)
0.0
0.1
0.2
0.3
0.4
L1
L2

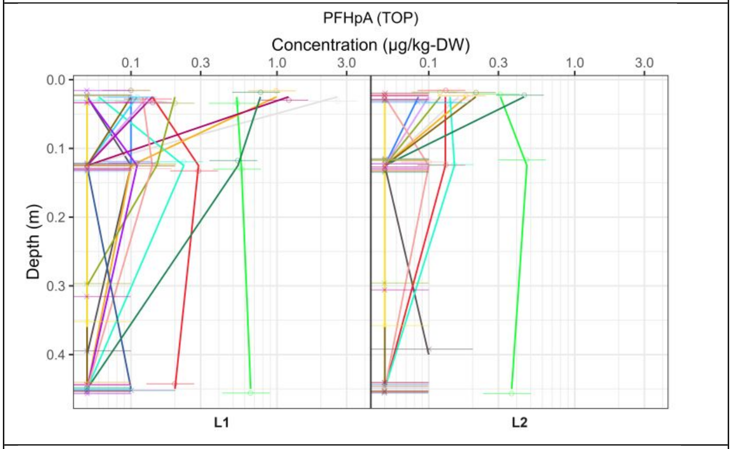
PFHpA (TOP)
Concentration (µg/kg-DW)
0.1
0.3
1.0
3.0
Depth (m)
0.0
0.1
0.2
0.3
0.4
L1
L2

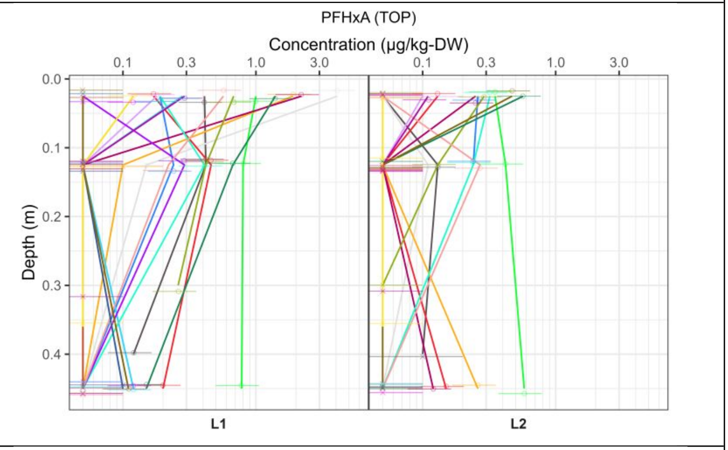
PFHxA (TOP)
Concentration (µg/kg-DW)
0.1
0.3
1.0
3.0
Depth (m)
0.0
0.1
0.2
0.3
0.4
L1
L2

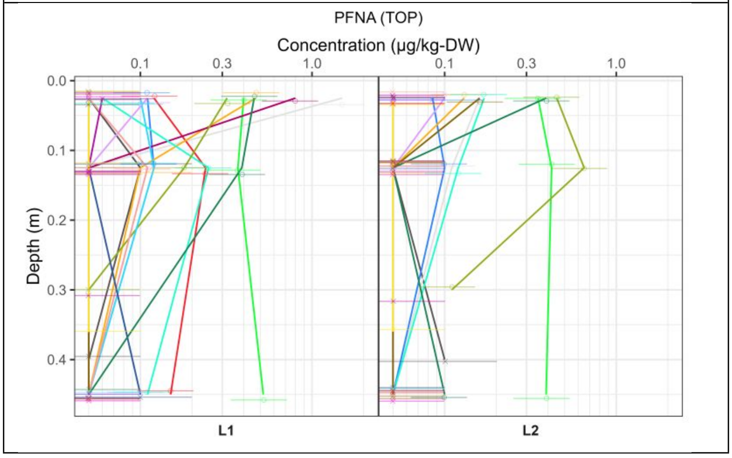
PFNA (TOP)
Concentration (µg/kg-DW)
0.1
0.3
1.0
Depth (m)
0.0
0.1
0.2
0.3
0.4
L1
L2

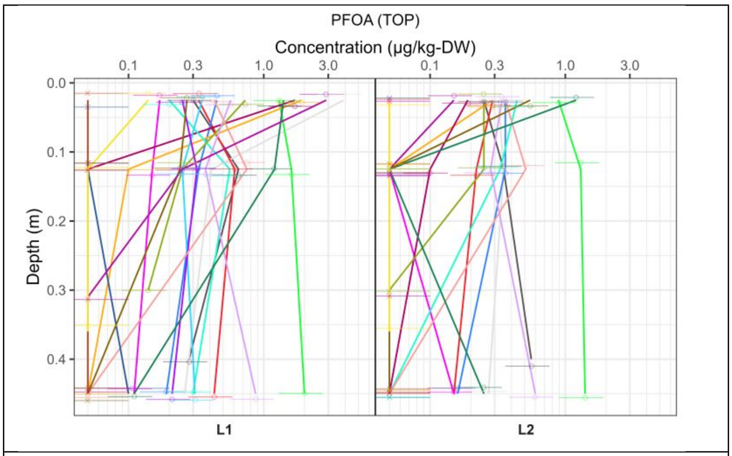
PFOA (TOP)
Concentration (µg/kg-DW)
0.1 0.3 1.0 3.0
Depth (m)
0.0 0.1 0.2 0.3 0.4
L1
L2

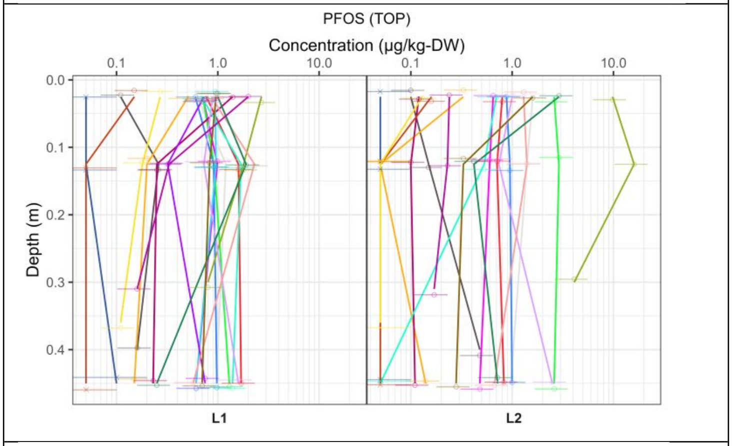
PFOS (TOP)
Concentration (µg/kg-DW)
0.1 1.0 10.0
Depth (m)
0.0 0.1 0.2 0.3 0.4
L1
L2

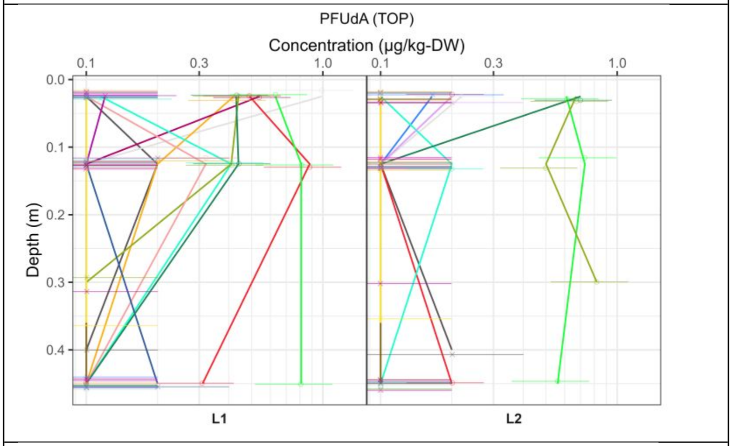
PFUdA (TOP)
Concentration (µg/kg-DW)
0.1 0.3 1.0
Depth (m)
0.0 0.1 0.2 0.3 0.4
L1
L2

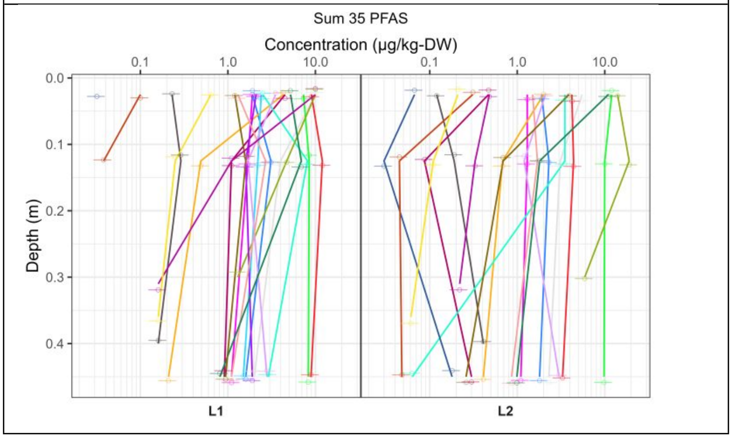
Sum 35 PFAS
Concentration (µg/kg-DW)
0.1 1.0 10.0
Depth (m)
0.0 0.1 0.2 0.3 0.4
L1
L2

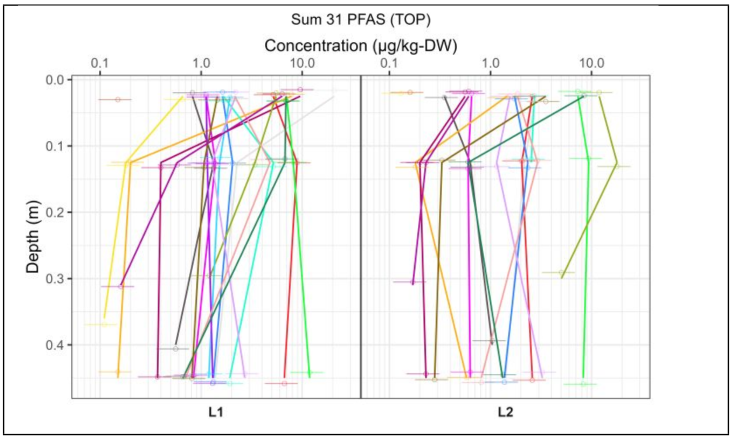

Figure S7. Concentrations of frequently quantified PFASs (before and after TOP) versus filter media depth in 20 sites studied

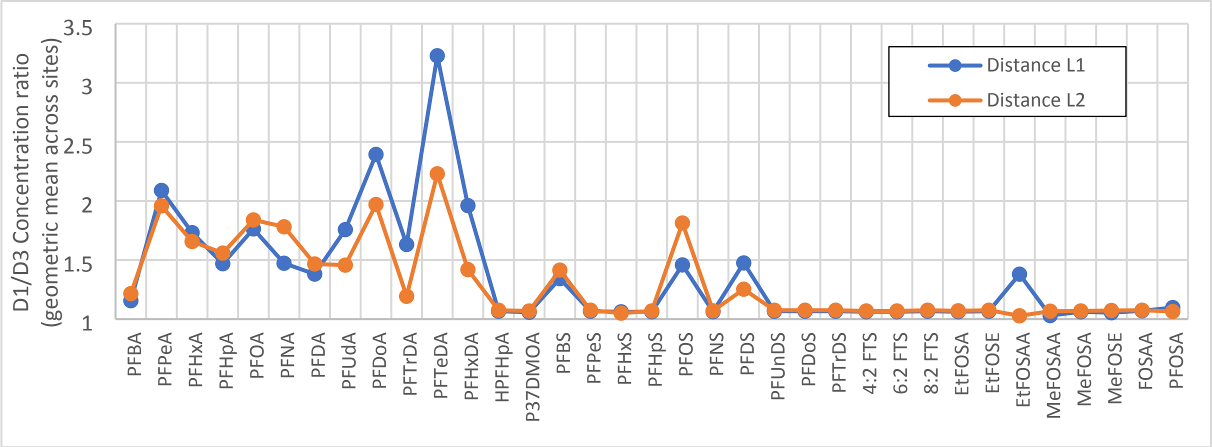

Figure S8. The geometric mean of the ratio of PFAS concentrations at D1 and D3 among 20 sites for two distances from the inlet

*Table S8. Percentage of Increase in PFAA concentrations after TOP assay relative to their targeted concentration (ΔPFAA/PFAA*100)*

Site #no.	point	PFBA	PFPeA	PFHxA	PFHpA	PFOA	PFNA	PFDA	PFUdA	PFDoA	PFTrDA	PFTeDA	PFHxDA	PFBS	PFHxS	PFHpS	PFOS	PFDS
1	FB			1433		700												
1	L1-D1			2633		1900												
1	L1-D2			2700		4233											18	
1	L1-D3			700		1767											0	
1	L2-D1					1567												
1	L2-D2			767		2233												
1	L2-D3					3633											17	
2	FB			12	11													
2	L1-D1			29	35	68												
2	L1-D2					185												
2	L1-D3					412	18										0	
2	L2-D1					127												
2	L2-D2					460												
2	L2-D3					400											14	
3	FB			2567		1167									1433		345	
3	L1-D1			3700	700	2767									347		65	
3	L1-D2			1367	833	1496	633	53	28	19					603		130	
3	L1-D3																	
3	L2-D1					1061									1167		83	
3	L2-D2			1700	567	1086		33							1500		109	
3	L2-D3							17									22	
4	FB					700												
4	L1-D1																	
4	L1-D2																	
4	L1-D3																	
4	L2-D1																	
4	L2-D2																	
4	L2-D3																	

5	FB																56	
5	L1-D1																50	
5	L1-D2																	
5	L1-D3																	
5	L2-D1																	
5	L2-D2																	
5	L2-D3																	
6	FB			1532	3367	1900	1433	340									57	
6	L2-D3					127	33										14	
6	L1-D1			1067	927	775	283	50	19								2	
6	L1-D2			605	575	233	359	17										
6	L1-D3			-600		224												
6	L2-D1			268	210	100	18			47							12	
6	L2-D2																5	
7	FB					767											80	
7	L1-D1				163	125		140									52	
7	L1-D2					100												
7	L1-D3			633													30	
7	L2-D1			327	233	139	98								567			
7	L2-D2																	
7	L2-D3																8	
8	FB					700												
8	L1-D1			1218	536	663												
8	L1-D2					158												
8	L1-D3																0	
8	L2-D1			633		163		120										
8	L2-D2																	
8	L2-D3																	
9	FB			2167	833	2700		140									0	
9	L1-D1			700		226		8										
9	L1-D2																	
9	L1-D3																	
9	L2-D1																	
9	L2-D2																	
9	L2-D3																	
10	L1-D1		255	595	2067	609	971	188	102					22			47	
10	L1-D2			114		242												
10	L1-D3					251											0	
10	L2-D1				33													
10	L2-D2			0		21												
10	L2-D3					8												
11	FB			2464	5947	1067	420	50	51						737		30	18
11	L1-D1			100	44	50												
11	L1-D2			188	21	49	33											
11	L1-D3			33	11	30												
11	L2-D1			103	59	71												
11	L2-D2				18	29												
11	L2-D3			200		58												
12	L1-D1			82		42		9										
12	L1-D2					121								8			5	
12	L1-D3			300														
12	L2-D1																	
12	L2-D2																	
12	L2-D3					7												
13	L1-D1			525	715	767	441	33		0								
13	L1-D2			515	482	742	335	35										30
13	L1-D3	5500		1014	746	1233	632	51									8	
13	L2-D1			46	7	27												
13	L2-D2			100	135	225	83							0			0	
13	L2-D3			530	185	383	144											0
14	FB		411	400	270	3833	833							633			633	
14	L1-D1			956	456	2400	96	0										
14	L1-D2			179	567	275	100											
14	L1-D3					313												
14	L2-D1			781		339		5										
14	L2-D3					171		5										
14	L2-D2			344		490	96	27										27
15	FB			4218	5456	2011	662	525	132								50	
15	L1-D1			4772	6500	2576	1206	133	5									
15	L1-D2																11	
15	L1-D3																25	
15	L2-D1				445	241	171											
15	L2-D2							6										
15	L2-D3			1633													8	
16	FB			632	1700	271	1100	380										
16	L1-D1			1194	1746	1033	627	112	4	12							40	
16	L1-D2																	
16	L1-D3																	
16	L2-D1			1567		476												
16	L2-D2					567											15	
16	L2-D3			161													0	
17	FB			4871	2071	1786	929	47										
17	L1-D1			805		43												
17	L1-D2			95	77	93	19											
17	L1-D3																	
17	L2-D1			220	100	132	55										6	
17	L2-D2			264	226	175	76											
17	L2-D3																	
18	L1-D1			100	25	74												
18	L1-D2			833	384	247	20	100										
18	L1-D3			1633		833												
18	L2-D1			81	9													
18	L2-D2			18		14											7	
18	L2-D3																11	
19	L1-D1			1767	175	119												
19	L1-D2					56												
19	L1-D3			700		308		0										
20	L1-D1					79												
20	L1-D2			1833	633	596												
20	L1-D3					180											1	

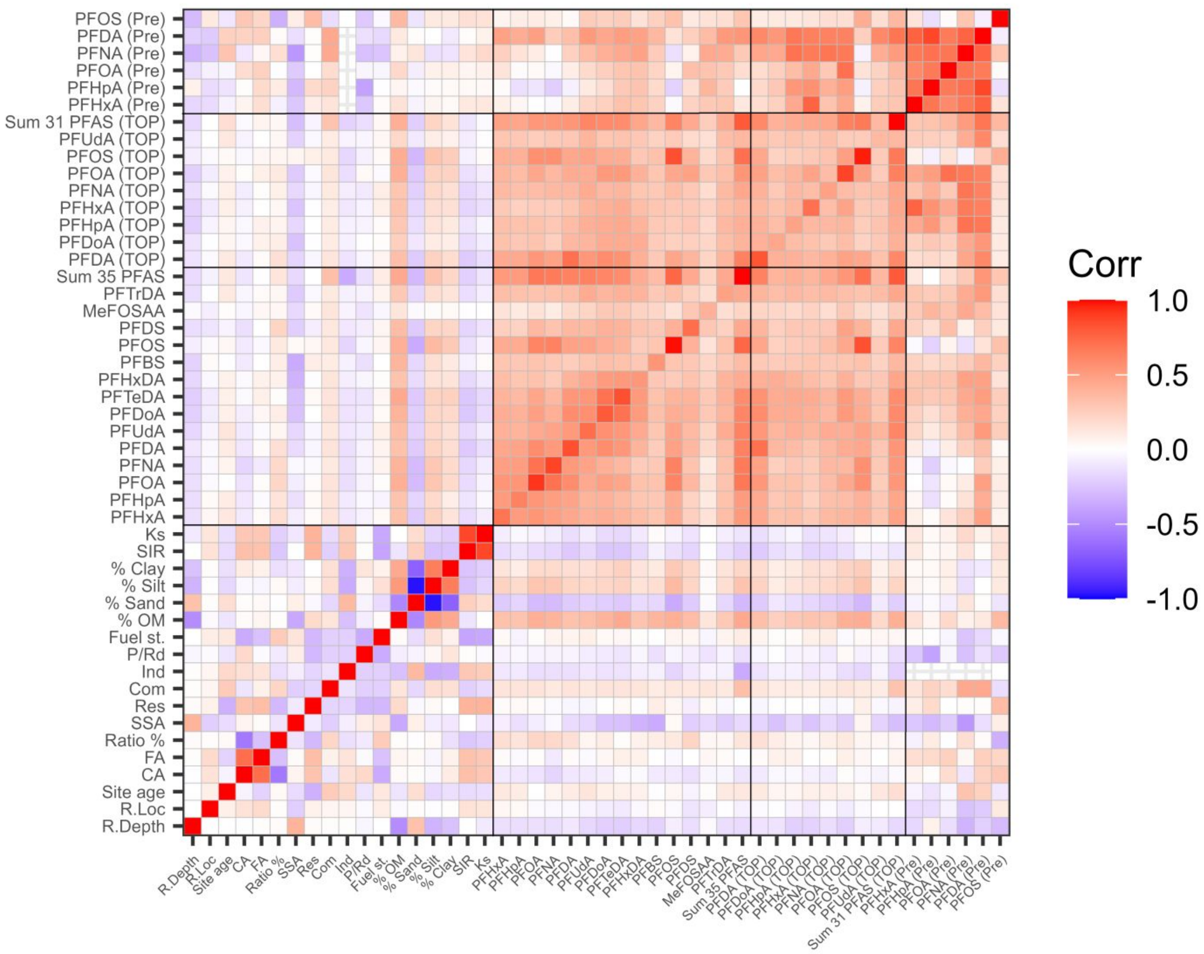

Figure S9. Heat map of Kendell's Tau correlation scores for environmental parameters and most frequently quantified PFASs (before and after TOP assay) and calculated unknown precursors (PFASs (Pre)) (p-values in Table S9)

Table S9. P-values for Kendell's Tau correlations shown in Figure S8

	R.Depth	R.Loc	Site age	CA	FA	Ratio %	SSA	Res	Com	Ind	P/Rd	Fuel st.	% OM	% Sand	% Silt	% Clay	SIR	Ks	PFHxA	PFHpA	PFOA	PFNA	PFDA	PFUdA	PFDoA	PFTeDA	PFHxDA	PFBS	PFOS	PFDS	MeFOSAA	PFTrDA	Sum 35 PFAS	PFDA (TOP)	PFDoA (TOP)	PFHpA (TOP)	PFHxA (TOP)	PFNA (TOP)	PFOA (TOP)	PFOS (TOP)	PFUdA (TOP)	Sum 31 PFAS (TOP)	PFHxA (Pre)	PFHpA (Pre)	PFOA (Pre)	PFNA (Pre)	PFDA (Pre)	PFOS (Pre)
**R.Depth**	0	0.973	0.95	0.909	0.86	0.757	0.004	0.815	0.877	0.877	0.646	0.978	0	0	0	0	1	0.352	0.02	0.008	0.031	0.017	0.095	0.002	0.001	0	0.001	0	0.235	0.009	0.61	0.002	0.034	0.04	0.014	0	0.001	0.009	0.001	0.246	0.066	0.03	0.102	0.594	0.124	0.058	0.252	0.03
**R.Loc**	0.973	0	0.321	0.056	0.012	0.922	0.131	0.407	0.582	0.582	0.798	0.338	0.758	0.931	0.892	0.879	0.108	0.179	0.474	0.609	0.719	0.64	0.629	0.374	0.154	0.12	0.523	0.679	0.727	0.036	0.107	0.721	0.386	0.756	0.822	0.293	0.088	0.626	0.162	0.757	0.971	0.951	0.174	0.283	0.521	0.143	0.229	0.433
**Site age**	0.95	0.321	0	0.471	0.007	0.653	0.231	0	0.001	0.018	0.195	0.157	0.11	0.014	0.014	0.049	0.083	0.098	0.038	0.077	0.978	0.321	0.082	0.022	0.189	0.165	0.166	0.819	0.538	0.418	0.892	0.026	0.081	0.061	0.31	0.316	0.16	0.144	0.241	0.649	0.011	0.017	0.596	0.318	0.635	0.053	0.151	0.47
**CA**	0.909	0.056	0.471	0	0	0	0.608	0	0.182	0.045	0.015	0	0.806	0.817	0.626	0.13	0	0	0.123	0.075	0.06	0.003	0.119	0.109	0.223	0.781	0.677	0.085	0.141	0.022	0.403	0.369	0.109	0.134	0.631	0.64	0.325	0.156	0.912	0.684	0.289	0.742	0.549	0.913	0.027	0.53	0.147	0.022
**FA**	0.86	0.012	0.007	0	0	0	0.843	0	0.403	0.094	0.816	0.001	0.415	0.697	0.56	0.312	0	0	0.716	0.75	0.85	0.13	0.782	0.733	0.5	0.188	0.171	1	0.615	0.929	0.38	0.709	0.851	0.768	0.181	0.218	0.968	0.836	0.204	0.506	0.96	0.365	0.077	0.122	0.003	0.818	0.117	0.093
**Ratio %**	0.757	0.922	0.653	0	0	0	0.312	0	0.008	0.096	0.246	0.001	0.895	0.848	0.659	0.043	0.003	0.012	0.004	0.013	0.001	0.002	0.012	0.373	0.158	0.819	0.168	0.097	0.073	0	0.402	0.458	0.056	0.01	0.103	0.293	0.443	0.143	0.08	0.401	0.429	0.531	0.554	0.434	0.894	0.258	0.887	0.007
**SSA**	0.004	0.131	0.231	0.608	0.843	0.312	0	0.531	0.129	0.617	0.516	0.322	0.001	0.496	0.464	0.388	0.99	0.395	0.558	0.242	0.502	0.782	0.191	0.128	0.022	0.019	0.002	0.001	0.824	0.066	0.132	0.037	0.164	0.116	0.012	0.116	0.028	0.136	0.061	0.666	0.062	0.021	0.093	0.06	0.077	0.032	0.501	0.753
**Res**	0.815	0.407	0	0	0	0	0.531	0	0.06	0.06	0.001	0.001	0.013	0.744	0.744	1	0	0	0.547	0.442	0.622	0.583	0.413	0.29	0.957	0.557	0.782	0.002	0.361	0.075	0.22	0.203	0.951	0.282	0.581	0.996	0.833	0.273	0.607	0.062	0.2	0.531	0.427	0.171	0.989	0.973	0.944	0.017
**Com**	0.877	0.582	0.001	0.182	0.403	0.008	0.129	0.06	0	0.211	0.029	0.029	0.102	0.026	0.025	0.042	0.037	0.13	0	0	0	0	0	0	0	0	#NUM!	#NUM!	0	0	#NUM!	#NUM!	0	0	#NUM!	0	0.001	0	0	0.001	#NUM!	0	0.223	0.098	0.07	0.011	0.017	0.334
**Ind**	0.877	0.582	0.018	0.045	0.094	0.096	0.617	0.06	0.211	0	0.029	0.029	0	0	0	0	0.003	0.008	0	0	0	0	0	0	0	0	#NUM!	#NUM!	0	0	#NUM!	#NUM!	0	0	#NUM!	0	0	0	0	0	#NUM!	0.023						1
**P/Rd**	0.646	0.798	0.195	0.015	0.816	0.246	0.516	0.001	0.029	0.029	0	0	0.368	0.554	0.639	0.098	0.88	0.821	0.205	0.506	0.333	0.315	0.271	0.065	0.046	0.017	0.197	0	0.412	0.097	0	0.093	0.107	0.389	0.656	0.178	0.212	0.017	0.524	0.181	0.181	0.089	0.035	0.005	0.705	0.129	0.494	0.123
**Fuel st.**	0.978	0.338	0.157	0	0.001	0.001	0.322	0.001	0.029	0.029	0	0	0.772	0.051	0.041	0.354	0	0	0.444	0.189	0.904	0.202	0.181	0.061	0.509	0.415	0.721	0.944	0.296	0.249	0.178	0.054	0.191	0.179	0.656	0.523	0.073	0.014	0.738	0.538	0.22	0.655	0.854	0.959	0.701	0.159	0.375	0.845
**% OM**	0	0.758	0.11	0.806	0.415	0.895	0.001	0.013	0.102	0	0.368	0.772	0	0	0	0	0.164	0.952	0	0	0	0	0	0	0	0	0	0	0	0	0.011	0	0	0	0	0	0	0	0	0	0	0	0.117	0.673	0.012	0.654	0.481	0.003
**% Sand**	0	0.931	0.014	0.817	0.697	0.848	0.496	0.744	0.026	0	0.554	0.051	0	0	0	0	0.001	0.024	0	0.001	0	0	0	0.001	0	0	0.006	0.002	0	0	0.728	0.001	0	0	0.046	0.01	0.003	0	0	0	0.068	0.002	0.591	0.859	0.346	0.371	0.933	0.364
**% Silt**	0	0.892	0.014	0.626	0.56	0.659	0.464	0.744	0.025	0	0.639	0.041	0	0	0	0	0.001	0.021	0	0.001	0	0	0	0.001	0.001	0	0.006	0.001	0	0	0.714	0.001	0	0	0.046	0.009	0.003	0	0	0	0.063	0.001	0.591	0.859	0.366	0.371	0.978	0.38
**% Clay**	0	0.879	0.049	0.13	0.312	0.043	0.388	1	0.042	0	0.098	0.354	0	0	0	0	0.002	0.008	0.013	0.108	0.002	0.002	0.006	0.007	0.001	0	0.009	0.036	0	0	0.545	0.007	0.001	0.004	0.091	0.044	0.007	0.005	0	0	0.144	0.029	0.743	0.817	0.304	0.398	0.554	0.38
**SIR**	1	0.108	0.083	0	0	0.003	0.99	0	0.037	0.003	0.88	0	0.164	0.001	0.001	0.002	0	0	0.052	0.153	0.06	0.029	0.004	0.036	0.002	0.002	0.023	0.443	0.104	0.019	0.935	0.045	0.014	0.004	0.043	0.132	0.015	0.017	0.091	0.48	0.266	0.177	0.318	0.792	0.571	0.414	0.939	0.361
**Ks**	0.352	0.179	0.098	0	0	0.012	0.395	0	0.13	0.008	0.821	0	0.952	0.024	0.021	0.008	0	0	0.348	0.721	0.527	0.284	0.064	0.282	0.05	0.038	0.208	0.811	0.55	0.119	0.974	0.337	0.179	0.079	0.264	0.557	0.093	0.136	0.397	0.844	0.881	0.808	0.274	0.804	0.459	0.228	0.676	0.382
**PFHxA**	0.02	0.474	0.038	0.123	0.716	0.004	0.558	0.547	0	0	0.205	0.444	0	0	0	0.013	0.052	0.348	0	0	0	0	0	0	0	0	0	0	0	0	0.001	0	0	0	0	0	0	0	0	0	0	0	0.38	0.88	0.081	0.091	0.001	0.585
**PFHpA**	0.008	0.609	0.077	0.075	0.75	0.013	0.242	0.442	0	0	0.506	0.189	0	0.001	0.001	0.108	0.153	0.721	0	0	0	0	0	0	0	0	0	0	0	0	0.015	0	0	0	0	0	0	0	0	0	0	0	0.699	0.267	0.782	0.305	0.003	0.377
**PFOA**	0.031	0.719	0.978	0.06	0.85	0.001	0.502	0.622	0	0	0.333	0.904	0	0	0	0.002	0.06	0.527	0	0	0	0	0	0	0	0	0	0	0	0	0.004	0	0	0	0	0	0	0	0	0	0	0	0.976	0.333	0.717	0.485	0.001	0.413
**PFNA**	0.017	0.64	0.321	0.003	0.13	0.002	0.782	0.583	0	0	0.315	0.202	0	0	0	0.002	0.029	0.284	0	0	0	0	0	0	0	0	0	0	0	0	0.053	0	0	0	0	0	0	0	0	0	0	0	0.811	0.076	0.709	0.94	0.093	0.47
**PFDA**	0.095	0.629	0.082	0.119	0.782	0.012	0.191	0.413	0	0	0.271	0.181	0	0	0	0.006	0.004	0.064	0	0	0	0	0	0	0	0	0	0	0	0	0	0	0	0	0	0	0	0	0	0	0	0	0.368	0.604	0.265	0.106	0.031	0.815
**PFUdA**	0.002	0.374	0.022	0.109	0.733	0.373	0.128	0.29	0	0	0.065	0.061	0	0.001	0.001	0.007	0.036	0.282	0	0	0	0	0	0	0	0	0	0	0	0	0	0	0	0	0	0	0	0	0	0	0	0	0.021	0.368	0.014	0.018	0	0.021
**PFDoA**	0.001	0.154	0.189	0.223	0.5	0.158	0.022	0.957	0	0	0.046	0.509	0	0	0.001	0.001	0.002	0.05	0	0	0	0	0	0	0	0	0	0	0	0	0	0	0	0	0	0	0	0	0	0	0	0	0.011	0.152	0.002	0.005	0.004	0.032
**PFTeDA**	0	0.12	0.165	0.781	0.188	0.819	0.019	0.557	0	0	0.017	0.415	0	0	0	0	0.002	0.038	0	0	0	0	0	0	0	0	0	0	0	0	0	0	0	0	0	0	0	0	0	0	0	0	0.001	0.017	0	0.003	0.001	0.021
**PFHxDA**	0.001	0.523	0.166	0.677	0.171	0.168	0.002	0.782	#NUM!	#NUM!	0.197	0.721	0	0.006	0.006	0.009	0.023	0.208	0	0	0	0	0	0	0	0	0	0	0	0	0	0	0	0	0	0	0	0	0	0	0	0	0.001	0.008	0	0.001	0.001	0.137
**PFBS**	0	0.679	0.819	0.085	1	0.097	0.001	0.002	#NUM!	#NUM!	0	0.944	0	0.002	0.001	0.036	0.443	0.811	0	0	0	0	0	0	0	0	0	0	0	0	0	0	0	0	0	0	0	0	0	0	0	0	0.009	0.097	0.002	0.065	0.011	0.019
**PFOS**	0.235	0.727	0.538	0.141	0.615	0.073	0.824	0.361	0	0	0.412	0.296	0	0	0	0	0.104	0.55	0	0	0	0	0	0	0	0	0	0	0	0	0.003	0	0	0	0	0	0	0	0	0	0	0	0.852	0.102	0.493	0.37	0.336	0.01
**PFDS**	0.009	0.036	0.418	0.022	0.929	0	0.066	0.075	0	0	0.097	0.249	0	0	0	0	0.019	0.119	0	0	0	0	0	0	0	0	0	0	0	0	0	0	0	0	0	0	0	0	0	0	0	0	0.011	0.151	0	0.654	0.105	0.337
**MeFOSAA**	0.61	0.107	0.892	0.403	0.38	0.402	0.132	0.22	#NUM!	#NUM!	0	0.178	0.011	0.728	0.714	0.545	0.935	0.974	0.001	0.015	0.004	0.053	0	0	0	0	0	0	0.003	0	0	0	0	0	0	0	0	0	0	0	0	0	0.001	0.001	0	0.002	0.019	0.155
**PFTrDA**	0.002	0.721	0.026	0.369	0.709	0.458	0.037	0.203	#NUM!	#NUM!	0.093	0.054	0	0.001	0.001	0.007	0.045	0.337	0	0	0	0	0	0	0	0	0	0	0	0	0	0	0	0	0	0	0	0	0	0	0	0	0.001	0.022	0	0.003	0	0.066
**Sum 35 PFAS**	0.034	0.386	0.081	0.109	0.851	0.056	0.164	0.951	0	0	0.107	0.191	0	0	0	0.001	0.014	0.179	0	0	0	0	0	0	0	0	0	0	0	0	0	0	0	0	0	0	0	0	0	0	0	0	0.186	0.989	0.022	0.059	0.001	0.015
**PFDA (TOP)**	0.04	0.756	0.061	0.134	0.768	0.01	0.116	0.282	0	0	0.389	0.179	0	0	0	0.004	0.004	0.079	0	0	0	0	0	0	0	0	0	0	0	0	0	0	0	0	0	0	0	0	0	0	0	0	0.051	0.091	0.023	0.002	0	0.371
**PFDoA (TOP)**	0.014	0.822	0.31	0.631	0.181	0.103	0.012	0.581	#NUM!	#NUM!	0.656	0.656	0	0.046	0.046	0.091	0.043	0.264	0	0	0	0	0	0	0	0	0	0	0	0	0	0	0	0	0	0	0	0	0	0	0	0	0.007	0.013	0.001	0.003	0	0.246
**PFHpA (TOP)**	0	0.293	0.316	0.64	0.218	0.293	0.116	0.996	0	0	0.178	0.523	0	0.01	0.009	0.044	0.132	0.557	0	0	0	0	0	0	0	0	0	0	0	0	0	0	0	0	0	0	0	0	0	0	0	0	0	0	0	0	0	0.301
**PFHxA (TOP)**	0.001	0.088	0.16	0.325	0.968	0.443	0.028	0.833	0.001	0	0.212	0.073	0	0.003	0.003	0.007	0.015	0.093	0	0	0	0	0	0	0	0	0	0	0	0	0	0	0	0	0	0	0	0	0	0	0	0	0	0	0	0	0	0.233
**PFNA (TOP)**	0.009	0.626	0.144	0.156	0.836	0.143	0.136	0.273	0	0	0.017	0.014	0	0	0	0.005	0.017	0.136	0	0	0	0	0	0	0	0	0	0	0	0	0	0	0	0	0	0	0	0	0	0	0	0	0.001	0	0	0	0	0.104
**PFOA (TOP)**	0.001	0.162	0.241	0.912	0.204	0.08	0.061	0.607	0	0	0.524	0.738	0	0	0	0	0.091	0.397	0	0	0	0	0	0	0	0	0	0	0	0	0	0	0	0	0	0	0	0	0	0	0	0	0	0	0	0	0	0.143
**PFOS (TOP)**	0.246	0.757	0.649	0.684	0.506	0.401	0.666	0.062	0.001	0	0.181	0.538	0	0	0	0	0.48	0.844	0	0	0	0	0	0	0	0	0	0	0	0	0	0	0	0	0	0	0	0	0	0	0	0	0.483	0.595	0.055	0.727	0.106	0.001
**PFUdA (TOP)**	0.066	0.971	0.011	0.289	0.96	0.429	0.062	0.2	#NUM!	#NUM!	0.181	0.22	0	0.068	0.063	0.144	0.266	0.881	0	0	0	0	0	0	0	0	0	0	0	0	0	0	0	0	0	0	0	0	0	0	0	0	0.003	0.031	0.001	0.003	0	0.091
**1 PFAS (TOP)**	0.03	0.951	0.017	0.742	0.365	0.531	0.021	0.531	0	0.023	0.089	0.655	0	0.002	0.001	0.029	0.177	0.808	0	0	0	0	0	0	0	0	0	0	0	0	0	0	0	0	0	0	0	0	0	0	0	0	0.002	0.009	0	0.001	0	0.003
**PFHxA (Pre)**	0.102	0.174	0.596	0.549	0.077	0.554	0.093	0.427	0.223		0.035	0.854	0.117	0.591	0.591	0.743	0.318	0.274	0.38	0.699	0.976	0.811	0.368	0.021	0.011	0.001	0.001	0.009	0.852	0.011	0.001	0.001	0.186	0.051	0.007	0	0	0.001	0	0.483	0.003	0.002	0	0	0	0	0	0.442
**PFHpA (Pre)**	0.594	0.283	0.318	0.913	0.122	0.434	0.06	0.171	0.098		0.005	0.959	0.673	0.859	0.859	0.817	0.792	0.804	0.88	0.267	0.333	0.076	0.604	0.368	0.152	0.017	0.008	0.097	0.102	0.151	0.001	0.022	0.989	0.091	0.013	0	0	0	0	0.595	0.031	0.009	0	0	0	0	0	0.586
**PFOA (Pre)**	0.124	0.521	0.635	0.027	0.003	0.894	0.077	0.989	0.07		0.705	0.701	0.012	0.346	0.366	0.304	0.571	0.459	0.081	0.782	0.717	0.709	0.265	0.014	0.002	0	0	0.002	0.493	0	0	0	0.022	0.023	0.001	0	0	0	0	0.055	0.001	0	0	0	0	0	0	0.874
**PFNA (Pre)**	0.058	0.143	0.053	0.53	0.818	0.258	0.032	0.973	0.011		0.129	0.159	0.654	0.371	0.371	0.398	0.414	0.228	0.091	0.305	0.485	0.94	0.106	0.018	0.005	0.003	0.001	0.065	0.37	0.654	0.002	0.003	0.059	0.002	0.003	0	0	0	0	0.727	0.003	0.001	0	0	0	0	0.001	0.241
**PFDA (Pre)**	0.252	0.229	0.151	0.147	0.117	0.887	0.501	0.944	0.017		0.494	0.375	0.481	0.933	0.978	0.554	0.939	0.676	0.001	0.003	0.001	0.093	0.031	0	0.004	0.001	0.001	0.011	0.336	0.105	0.019	0	0.001	0	0	0	0	0	0	0.106	0	0	0	0	0	0.001	0	0.805
**PFOS (Pre)**	0.03	0.433	0.47	0.022	0.093	0.007	0.753	0.017	0.334	1	0.123	0.845	0.003	0.364	0.38	0.38	0.361	0.382	0.585	0.377	0.413	0.47	0.815	0.021	0.032	0.021	0.137	0.019	0.01	0.337	0.155	0.066	0.015	0.371	0.246	0.301	0.233	0.104	0.143	0.001	0.091	0.003	0.442	0.586	0.874	0.241	0.805	0

Comparison with other studies of urban surface soils and stormwater sediments

Table S10. Comparing the concentrations of most frequently found PFASs (minimum; median/mean; maximum in µg/kg-DW) in this study with those in various urban soil/sediment media reported by other studies

Reference	Media	Land use	Place	PFBA	PFPeA	PFHxA	PFHpA	PFOA	PFNA	PFDA	PFUdA	PFDoA	PFTeDA	PFHxDA	PFBS	PFHxS	PFOS	PFDS	6:2 FTS	EtFOSAA	MeFOSAA	EtFOSA	MeFOSA	EtFOSE	MeFOSE	Sum of all PFAS analyzed
This study	Stormwater forebay sediments	Mixed urban area	Ohio, Michigan, and Kentucky (USA)	<0.1; <0.1; 0.34	<0.03; 0.0325; 1.2	<0.03; 0.0325; 3.2	<0.03; <0.03; 0.19	<0.03; <0.03; 0.63	<0.03; <0.03; 0.28	<0.1; <0.1; 1.6	<0.1; <0.1; 0.73	<0.1; 0.185; 5.2	<0.03; 0.115; 3.8	<0.03; 0.035; 1.4	<0.03; <0.03; 0.076	<0.03; <0.03; <0.043	<0.03; 0.145; 1.1	<0.03; <0.03; 0.24	<0.043	<0.1; <0.1; 0.39	<0.03; 0.0385; 0.19	<0.2; <0.2; <0.29	<0.03; <0.03; <0.043	<0.1; <0.1; <0.15	<0.03; <0.03; 0.1	0.064; 0.665; 16
	Stormwater biofilter media			<0.1; <0.1; 0.28	<0.03; 0.0355; 1.1	<0.03; <0.03; 0.59	<0.03; <0.03; 0.29	<0.03; 0.066; 0.7	<0.03; 0.0515; 1.2	<0.1; 0.16; 3.6	<0.1; <0.1; 1.2	<0.1; 0.11; 4	<0.03; 0.0465; 1.6	<0.03; <0.03; 0.79	<0.03; <0.03; 0.27	<0.03; <0.03; 0.059	<0.03; 0.7; 15	<0.03; <0.03; 0.81	<0.03; <0.03; <0.059	<0.1; <0.1; 3.6	<0.03; <0.03; 0.48	<0.2; <0.2; 0.4	<0.03; <0.03; 0.059	<0.1; <0.1; 0.2	<0.03; <0.03; 0.08	<0.03; 1.6; 19
(Flanagan et al., 2021)[24]	Stormwater pond sediments	Mixed urban area	Sweden	<0.5; <0.5; 1.42	<0.5; <0.5; 0.895	<0.5; <0.5; 0.635					<0.5; <0.5; 0.872	<0.5; <0.5; 1.46	<0.5; <0.5; 1.35				<0.5; <0.5; 3.18	<0.5; <0.5; 0.557				<0.5; <0.5; 0.735	<0.5; <0.5; 0.584	<0.5; <0.5; 1.19	<0.5; <0.5; 0.825	
(Crane et al., 2019)[25]	Stormwater pond sediments	Mixed urban + industrial area	Twin Cities, MN	<0.083; 0.16; 0.43	<0.093; 0.11; 0.185	<0.142	<0.142	<0.095; 0.18; 0.75	<0.095; 0.17; 0.592	<0.095; 0.17; 0.83	<0.095; 0.24; 1.09	<0.095; 0.37; 1.41			<0.284	<0.195; 0.29; 0.537	0.3; 0.84; 2.25									
(Wei et al., 2023)[26]	Stormwater gully pot sediments	Mixed urban area	Stockholm, Sweden	ND	ND	ND	ND	ND	ND	ND	ND	ND	ND	ND	ND	ND	0.64; NA; 2.17	ND	0.00116	ND	ND	ND	ND	ND	ND	
(Xiao et al., 2012)[27]	Runoff sediment/debris	Near an industrial source of PFAS	Minneapolis, MN (USA)				ND	ND	ND	ND	ND						19.8; 120.5; 590									
(Gao et al., 2019)[28]	Road dust	Around a PFAS-related industrial area	Wuhan, China	0.27; 3.37; 313	<0.05; 0.52; 61.2	0.09; 1.43; 129	<0.01; 0.51; 39.5	0.32; 0.55; 21.9	<0.01; 0.04; 0.25	<0.01; 0.06; 0.26					0.05; 0.18; 18.1	0.19; 2.33; 46.1	0.06; 0.71; 458									1.3; 8.36; 914
(Wang et al., 2010)[29]	Road dust	Within 1 km distance from a PFAS-related industry	Wuhan, China					ND; 0.67; 1.91								0.07; 0.5; 2.24	3.27; NA; 283									
(Ahmadiresketi et al., 2022)[30]	Road sweepings	Mixed urban area (mostly residential)	Gainesville, FL (USA)	ND; 0.45; 2.64	ND; 0.42; 1.49	ND; 0.33; 0.66	ND; 0.19; 0.7	0.02; 0.1; 1.7	ND; 0.08; 0.53	ND; 0.1; 0.26	ND; 0.05; 0.21	ND; 0.1; 0.47	ND; 0.07; 0.26	ND; 0.03; 0.06	ND; 0.21; 0.36	ND; 0.1; 0.12	ND; 0.13; 0.51	ND; 0.16; 0.23	ND; 0.06; 0.9	ND; 0.17; 18.16	ND; 0.02; 0.73					0.02; NA; 47.2
(Codling et al., 2014)[31]	Lake sediments	-	L. Michigan (USA)	ND; 0.57; 0.97	ND; 0.17; 1.30			ND; 0.21; 0.42	ND; 0.08; 0.42	ND; 0.03; 0.09		ND; 0.07; 0.18			ND; 0.19; 0.56	ND; 0.53; 1.89	ND; 0.45; 1.15									
(Codling et al., 2018a)[32]	Lake sediments	-	Northern L. Michigan; L. Superior; L. Huron (USA)		ND; 1.6; 5.2	0.1; 0.78; 4.2	ND; 0.89; 8.5	ND; 0.35; 3.0	0.0; 0.7; 0.8	ND	ND; 1.18; 14.7	0.2; 0.8; 1.3			0.2; 0.76; 9.0	ND; 0.9; 1.8	0.1; 0.65; 2.5									
(Codling et al., 2018b)[33]	Lake sediments	-	L. Ontario; L. St. Clair; L. Erie (USA)	0.4; 21.3; 56.3	0.1; 1.73; 5.2	0.1; 0.54; 4.2	0.0; 0.34; 6.8	0.0; 0.49; 1.7	0.1; 0.28; 1.0	0.1; 1.12; 10.4	0.0; 2.0; 21.2	0.0; 1.6; 11.8			0.3; 11.0; 42.2	0.1; 0.9; 5.2	0.1; 1.56; 13.2									
(Rankin et al., 2016)[34]	Surface soil		Across north America			0.032; 0.229; 1.986	0.017; 0.167; 1.295	0.022; 0.363; 1.838	0.015; 0.101; 1.061	0.005; 0.051; 0.962	ND; 0.063; 1.508	ND; 0.020; 0.793	ND; ND; 0.177			ND; 0.006; 0.036	0.018; 0.226; 1.956	ND; ND; 0.127								0.184; 1.712; 6.437
(Gasperi et al., 2018)[35]	Surface soil	Mixed Urban + industrial area	Across the Greater Paris (France)		ND	ND	ND	0.07; 0.15; 0.5	0.024; 0.035; 0.07	0.056; 0.063; 0.09	0.032; 0.038;0.05	0.018; 0.022; 0.04	ND		0.007; 0.017; 0.05	0.012; 0.04; 0.13	0.3; 0.63; 2.1	0.004; 0.007; 0.015	0.02	0.013; 0.027; 0.07	0.033	ND	0.004			0.33; 0.7; 2.3
(Zhu and Kannan, 2019)[36]	Surface soil	Hocking well field around a PFAS-related industrial area	Ohio, USA				0.876; 1.48; 6.58	46.8; 93.2; 466	1.62; 2.49; 6.27	2.1; 4.49; 5.27	2.58; 7.34; 13.9	2.21; 4.46; 11.4														71.5; 114; 492
(Washington et al., 2008)[37]	Surface soil	Picnic areas	Northeastern Georgia (USA)			ND; 0.014; 1.584	ND; ND; 0.592	ND; 0.495; 1.174	ND; 0.192; 0.411	ND; 0.2285; 0.407																
	Surface soil	Commercial area				ND; 0.099; 0.54	ND; 0.0745; 0.544	0.031; 0.499; 0.811	ND; 0.038; 0.36	ND; 0.018; 0.169																
(Li et al., 2010)[38]	Surface soil	Dense residential-Industrial area	Shanghai, China	0.15; 0.5005; 1.48	ND; 0.5855; 1.73	ND; 0.273; 2.01	0.147; 0.381; 1.29	3.28; 42.55; 47.5	ND; 0.1985; 1.21	0.179; 0.35; 1.07	ND; 0.0785; 0.515	ND; ND; 0.536	ND; ND; 0.112		ND; 0.0675; 0.254	ND; ND; 0.276	8.58; 9.48; 10.4									
(Naile et al., 2013)[39]	Surface soil	Mixed urban + industrial areas	West coast of Korea		ND	ND	ND	<0.2; 2.2; 3.4	ND	ND	ND	ND			ND	ND	<0.2; 0.82; 1.7	ND								0.28; 2.1; 3.9

Redundancy analysis (RDA)

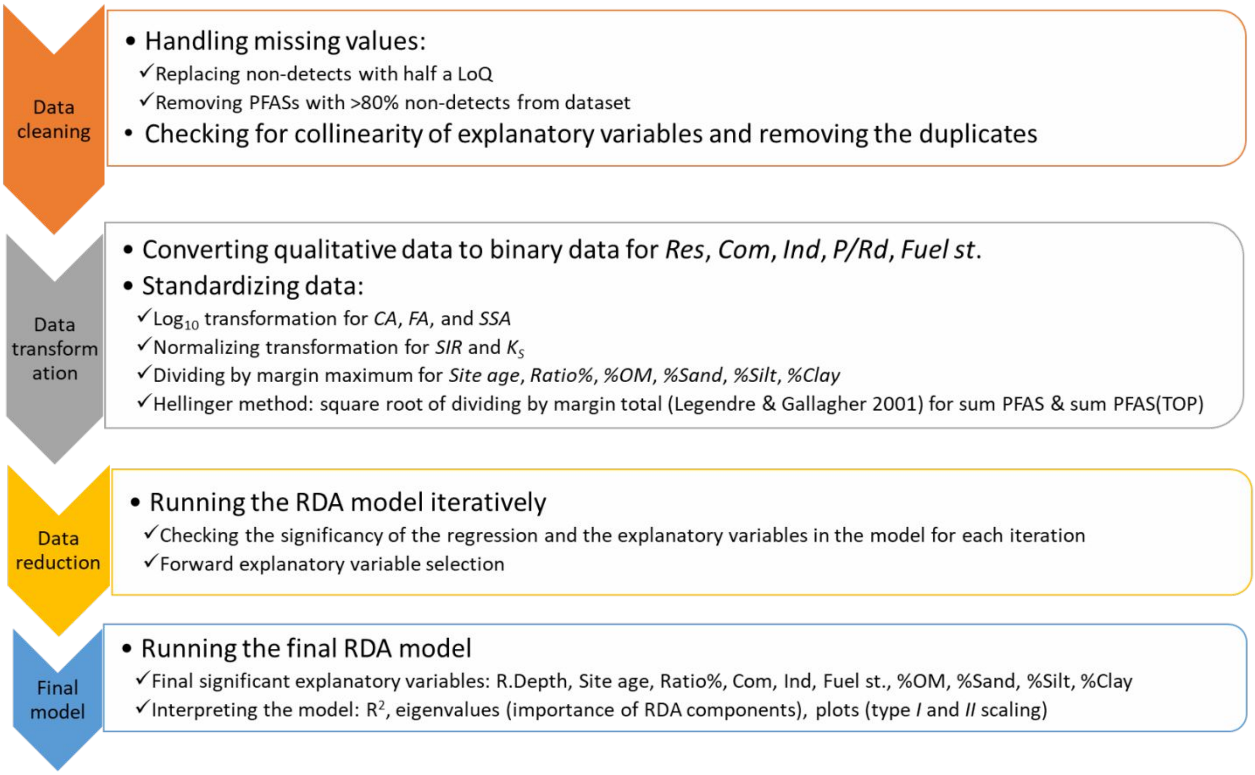

Figure S10. Data treatment process for RDA

Table S11. Final RDA model's report summary

> RsquareAdj(rda.model)
r.squared=0.4506185
adj.r.squared= 0.3972805

> anova.cca(rda.model)
Permutation test for rda under reduced model
Permutation: free
Number of permutations: 999

Model: rda(formula = response_data ~ R.Depth + `Site age` + `Ratio %` + Com + Ind + `Fuel st.` + `% OM` + `% Sand` + `% Silt` + `% Clay`, data = explanatory_data, scale = TRUE, na.action = na.exclude)

	Df	Variance	F	Pr(>F)
Model	10	10.815	8.4484	0.001 ***
Residual	103	13.185		

Signif. codes: 0 '***' 0.001 '**' 0.01 '*' 0.05 '.' 0.1 ' ' 1

> anova.cca(rda.model, by = "terms")
Permutation test for rda under reduced model
Terms added sequentially (first to last)
Permutation: free

```
Number of permutations: 999

Model: rda(formula = response_data ~ R.Depth + `Site age` + `Ratio %` + Com + Ind + `Fuel st.` + `% OM` + `% Sand` + `% Silt` + `% Clay`, data = explanatory_data, scale = TRUE, na.action = na.exclude)
             Df    Variance    F         Pr(>F)
R.Depth      1     0.8592    6.7123    0.001 ***
`Site age`   1     0.9129    7.1312    0.002 **
`Ratio %`    1     0.6155    4.8085    0.005 **
Com          1     2.7762    21.6870   0.001 ***
Ind          1     0.8074    6.3074    0.004 **
`Fuel st.`   1     0.3011    2.3520    0.065 .
`% OM`       1     1.9518    15.2471   0.001 ***
`% Sand`     1     1.3717    10.7156   0.001 ***
`% Silt`     1     0.8596    6.7151    0.005 **
`% Clay`     1     0.3594    2.8073    0.088 .
Residual   103     13.1852
---
Signif. codes:  0 '***' 0.001 '**' 0.01 '*' 0.05 '.' 0.1 ' ' 1
----------------------------------------------------------------------------------------
```

> anova.cca(rda.model, by = "terms")

```
Permutation test for rda under reduced model
Terms added sequentially (first to last)
Permutation: free
Number of permutations: 999

Model: rda(formula = response_data ~ R.Depth + `Site age` + `Ratio %` + Com + Ind + `Fuel st.` + `% OM` + `% Sand` + `% Silt` + `% Clay`, data = explanatory_data, scale = TRUE, na.action = na.exclude)
             Df   Variance     F         Pr(>F)
R.Depth      1    0.8592     6.7123    0.001 ***
`Site age`   1    0.9129     7.1312    0.001 ***
`Ratio %`    1    0.6155     4.8085    0.008 **
Com          1    2.7762     21.6870   0.001 ***
Ind          1    0.8074     6.3074    0.004 **
`Fuel st.`   1    0.3011     2.3520    0.069 .
`% OM`       1    1.9518     15.2471   0.001 ***
`% Sand`     1    1.3717     10.7156   0.001 ***
`% Silt`     1    0.8596     6.7151    0.001 ***
`% Clay`     1    0.3594     2.8073    0.092 .
Residual   103    13.1852
---
Signif. codes:  0 '***' 0.001 '**' 0.01 '*' 0.05 '.' 0.1 ' ' 1
-------------------------------------------------------------------
```

> RDA model summary:

```
Partitioning of correlations:
                  Inertia    Proportion
Total             24.00      1.0000
Constrained       10.81      0.4506
Unconstrained     13.19      0.5494

Eigenvalues, and their contribution to the correlations
```

Importance of components:

	RDA1	RDA2	RDA3	RDA4	RDA5	RDA6	RDA7	RDA8
Eigenvalue	8.7691	1.04626	0.37658	0.25416	0.18907	0.08137	0.051406	0.028495
Proportion Explained	0.3654	0.04359	0.01569	0.01059	0.007878	0.00339	0.002142	0.001187
Cumulative Proportion	0.3654	0.40897	0.42467	0.43526	0.443133	0.44652	0.448666	0.449853
	RDA9	RDA10	PC1	PC2	PC3	PC4	PC5	PC6
Eigenvalue	0.009554	0.008813	4.9217	3.1063	1.5623	0.98233	0.76153	0.42059
Proportion Explained	0.000398	0.000367	0.2051	0.1294	0.0651	0.04093	0.03173	0.01752
Cumulative Proportion	0.450251	0.450619	0.6557	0.7851	0.8502	0.89115	0.92288	0.9404
	PC7	PC8	PC9	PC10	PC11	PC12	PC13	
Eigenvalue	0.35817	0.25043	0.20128	0.179588	0.14249	0.068287	0.049084	
Proportion Explained	0.01492	0.01043	0.008387	0.007483	0.005937	0.002845	0.002045	
Cumulative Proportion	0.95532	0.96576	0.974146	0.981629	0.987566	0.990411	0.992457	
	PC14	PC15	PC16	PC17	PC18	PC19	PC20	
Eigenvalue	0.041332	0.03451	0.027631	0.020127	0.01373	0.013025	0.010284	
Proportion Explained	0.001722	0.001438	0.001151	0.000839	0.000572	0.000543	0.000429	
Cumulative Proportion	0.994179	0.995617	0.996768	0.997607	0.998179	0.998721	0.99915	
	PC21	PC22	PC23	PC24				
Eigenvalue	0.008008	0.005542	0.004424	0.002433				
Proportion Explained	0.000334	0.000231	0.000184	0.000101				
Cumulative Proportion	0.999483	0.999714	0.999899	1				

Accumulated constrained eigenvalues
Importance of components:

	RDA1	RDA2	RDA3	RDA4	RDA5	RDA6	RDA7	RDA8
Eigenvalue	8.7691	1.04626	0.37658	0.2542	0.18907	0.081372	0.051406	0.028495
Proportion Explained	0.8108	0.09674	0.03482	0.0235	0.01748	0.007524	0.004753	0.002635
Cumulative Proportion	0.8108	0.90759	0.94241	0.9659	0.98339	0.990914	0.995667	0.998302
	RDA9	RDA10						
Eigenvalue	0.009554	0.008813						
Proportion Explained	0.000883	0.000815						
Cumulative Proportion	0.999185	1						

Scaling 2 for species and site scores
* Species are scaled proportional to eigenvalues
* Sites are unscaled: weighted dispersion equal on all dimensions
* General scaling constant of scores: 7.21643

Species scores

	RDA1	RDA2	RDA3	RDA4	RDA5	RDA6
PFHxA	-1.0404	-0.27881	-0.0967	0.14694	0.146557	-0.05397
PFHpA	-0.9013	-0.02903	-0.2117	-0.06097	0.192594	-0.06652
PFOA	-1.0003	-0.12933	0.12421	-0.00807	0.293241	-0.12566
PFNA	-0.6353	-0.47585	0.06661	0.090228	-0.06931	-0.02574
PFDA	-0.6244	-0.00734	-0.16182	-0.28773	0.031813	0.037928

PFUdA	-0.9373	-0.12312	-0.04638	0.035515	-0.24349	0.072303
PFDoA	-0.8707	0.22229	-0.21604	-0.16581	-0.0249	0.072173
PFTeDA	-1.0731	0.13212	-0.06284	0.048752	0.066782	0.031585
PFHxDA	-1.0389	0.06157	-0.09046	0.033824	0.107719	0.107188
PFBS	-1.0728	0.49648	-0.01844	0.217263	0.045644	-0.17656
PFOS	-0.5838	-0.47658	0.15852	0.053291	-0.13875	-0.00783
PFDS	-0.7074	0.60706	0.61205	-0.05297	-0.01243	0.076558
MeFOSAA	-0.6887	0.75714	-0.13804	0.087963	-0.25054	-0.14134
PFTrDA	-0.9327	-0.06493	0.02431	0.11522	-0.02083	0.066531
Sum 35 PFAS inc. ½LOQ	-1.1151	-0.20548	0.18365	-0.10876	-0.02432	-0.04588
PFDA (TOP)	-0.7699	-0.02417	-0.16421	-0.23621	0.025647	0.058642
PFDoA (TOP)	-0.7842	0.26225	-0.21992	-0.1259	-0.04142	0.062651
PFHpA (TOP)	-0.8603	-0.05412	-0.03815	0.125982	0.055922	0.047785
PFHxA (TOP)	-0.8251	-0.17699	-0.0136	0.110484	0.001473	0.047574
PFNA (TOP)	-0.9879	-0.16094	-0.03083	0.215354	-0.03899	0.013493
PFOA (TOP)	-1.0098	0.15429	0.29341	-0.06293	0.12579	0.114903
PFOS (TOP)	-0.5278	-0.42842	0.08752	0.056699	-0.17071	-0.0187
PFUdA (TOP)	-0.9959	0.03766	-0.11826	0.080936	-0.16144	0.074204
Sum 31 PFAS (TOP) inc. ½LOQ	-0.9984	-0.25004	0.12973	-0.39672	-0.14344	-0.18346

Site scores (weighted sums of species scores)

	RDA1	RDA2	RDA3	RDA4	RDA5	RDA6
2	0.49025	0.1457071	0.124531	0.242365	0.153881	0.52359
3	0.29588	-0.1310357	0.387196	-1.093034	-0.630467	-0.88255
4	0.53495	0.1280796	-0.012722	0.175948	0.126233	0.28551
5	0.54832	0.1525272	-0.045375	0.187126	0.150165	0.27481
6	0.52732	0.1322760	0.032825	0.167288	0.160001	0.36930
7	0.32858	-0.1258574	0.392235	-1.224074	-0.765906	-1.19700
8	-0.06871	-0.6637671	0.476533	-0.391710	2.351785	-1.87955
9	0.33441	-0.2089591	0.411695	-0.245940	0.426810	-0.41305
10	0.14335	-0.2538404	1.566872	-0.389991	0.842833	-0.67313
11	0.13680	-0.4821031	0.550122	-1.498648	-0.307948	-2.25335
12	0.44900	-0.0735809	0.314628	-0.137672	-0.049470	-0.10632
13	0.21605	-0.4988014	1.242324	-0.597336	-0.165794	-1.06317
14	0.22623	0.8096353	0.043328	0.811208	-1.056570	-1.50578
15	-0.09678	0.8204228	-0.012179	-0.022082	-2.614938	-1.16430
16	0.49153	-0.0247498	-0.091368	-0.104763	-0.093977	-0.10505
17	0.27183	0.5877009	0.351653	0.547140	-0.702668	-1.60779
18	0.15100	0.9779304	-0.016216	0.392556	-1.432822	-2.50271
19	0.49686	-0.0162277	-0.087617	-0.055416	-0.070921	-0.16611
20	0.64462	0.3144155	-0.429152	1.279611	0.597275	1.59156
21	0.69155	0.3778357	-0.590855	1.420199	0.631038	1.77291
22	0.55026	0.2264571	-0.505958	0.561087	-0.037589	0.80562
23	0.64726	0.3048061	-0.418831	1.276856	0.577492	1.57915
24	0.64788	0.3090628	-0.422764	1.274896	0.584350	1.58005
25	0.64516	0.3043206	-0.412065	1.266385	0.580584	1.56827

26	0.56762	0.1292546	-0.172600	0.248029	0.046435	0.07687
27	0.64775	0.3081424	-0.421914	1.275320	0.582867	1.57985
28	0.69155	0.3778357	-0.590855	1.420199	0.631038	1.77291
29	0.51573	0.1354271	-0.100483	0.456889	0.219099	-0.60087
30	0.64763	0.3073371	-0.421170	1.275690	0.581570	1.57968
31	0.64758	0.3069920	-0.420851	1.275849	0.581014	1.57961
32	-1.02870	-0.1916924	-0.741667	0.320458	0.579820	3.02725
33	-1.17006	-0.5306742	-0.884352	-0.165746	1.415852	-0.31407
34	0.48466	0.0534741	-0.196382	-0.113151	0.007309	0.24422
35	-2.25267	-0.9467333	-2.079359	-0.151974	3.208052	-0.72099
36	0.33836	-0.2003691	-0.271773	-0.264903	0.539373	-0.57025
37	0.42193	-0.0865991	-0.041908	-0.302937	-0.083392	0.08868
38	0.35845	-0.1753992	0.073804	0.047316	0.713690	-0.80520
39	0.35579	-0.2153353	0.096822	0.080891	0.701563	-0.89673
40	0.48729	-0.0673749	-0.005995	0.114147	-0.026912	-0.32552
41	-0.17629	-0.5026874	0.471104	0.110406	1.189223	-1.29440
42	0.51913	0.0187813	-0.078630	0.191662	0.084187	-0.13222
43	0.55892	0.0903083	-0.133650	0.235364	-0.025277	0.02532
44	-0.79955	1.0785457	7.840378	-2.674142	1.295752	3.44464
45	0.45953	0.0922356	0.501510	0.098267	0.388302	-0.15595
46	0.56646	0.1213118	-0.165631	0.251773	0.033021	0.07483
47	0.50241	0.0574590	0.103759	0.133506	0.195126	-0.04319
48	0.54263	0.0492093	-0.088197	0.267809	0.109922	-0.12110
49	0.55364	0.1034250	-0.124836	0.242009	0.156955	-0.06965
50	0.49606	0.0461454	-0.108579	0.039012	0.162475	0.07200
51	0.56206	0.1048951	-0.144992	0.248994	0.009146	0.06004
52	0.56867	0.1295653	-0.172983	0.268880	0.054180	0.10655
53	0.56495	0.1177503	-0.156597	0.263983	0.037718	0.09273
54	0.64392	0.2958073	-0.404197	1.270304	0.566869	1.56647
55	0.64736	0.3054964	-0.419469	1.276538	0.578604	1.57929
56	-4.00563	-0.7270780	-2.160948	5.360879	6.794294	8.10486
57	-0.05128	-0.1459162	-0.013432	-0.536760	0.310584	0.11023
58	0.32080	-0.0022853	0.172262	-0.172622	-0.168098	-0.24477
59	-0.37645	-0.7062730	0.236903	-0.290379	1.551393	-2.15792
60	0.07661	-0.7479297	0.220536	-0.064829	1.672333	-2.26074
61	0.22270	-0.3117919	0.379796	-0.262177	1.228979	-1.85666
62	-1.29725	0.7510102	-3.331725	-2.870391	0.203558	5.82812
63	-2.05959	0.0003291	-5.146369	-5.562641	0.360394	5.98795
64	-1.07605	-1.1169634	-3.402247	-7.818482	2.301476	1.53508
65	-0.16363	-0.2769179	-1.071305	-1.659134	0.517674	0.82545
66	-0.19921	-0.0713570	-1.344959	-1.392744	1.093604	0.48283
67	0.01766	-0.3330860	-1.164889	-2.540354	0.289415	0.58707
68	0.29240	-0.1995541	-0.056829	0.288599	0.927984	-1.88860
69	0.25949	0.2992362	0.217189	0.866609	0.483579	-2.99304
70	0.39683	-0.0475624	0.104299	0.472773	0.546233	-1.51025
71	0.37994	-0.1364806	-0.012090	0.505442	0.566623	-1.69737
72	0.40453	-0.0667520	-0.003047	0.474540	0.457408	-1.52414
73	0.42439	-0.1915382	0.153393	0.218470	0.653498	-0.96232
74	-1.59978	0.8373773	-1.341763	0.769211	-0.146652	5.67767
75	-1.91400	2.5291983	-1.418777	0.350914	-2.863157	2.73139
76	-1.88528	2.8390907	0.182674	-0.547743	-4.252505	2.90382
77	-2.10715	0.3397910	0.363761	-0.812300	3.086381	-5.41718

78	-2.13956	2.1004363	0.362282	-0.560409	-0.775960	-4.40200
79	-1.76997	2.6626183	0.896523	-0.462800	-3.253502	-2.99916
80	0.22342	0.0688780	0.381477	-0.129459	-0.177678	1.33431
81	-0.01691	0.6087918	1.534380	0.445768	-0.003355	0.12825
82	0.40513	0.0358285	0.791901	-0.302489	-0.228330	0.07263
83	0.05592	-0.0850445	0.505548	-0.786007	-0.383966	-2.12695
84	0.13357	0.6191510	1.316689	0.274104	-0.169314	-0.63450
85	0.37523	0.1072862	1.014393	-0.435701	-0.207185	0.15673
86	-0.93846	0.7290578	0.877374	-0.296667	-1.285885	5.30105
87	0.37304	-0.0981280	-0.028388	-1.173076	-0.731823	-1.47231
88	0.55363	0.1121651	-0.131011	0.245292	0.228287	-0.10837
89	0.20151	-0.0477371	-0.316853	-0.556490	0.378083	0.65927
90	0.52563	0.1159167	-0.235560	-0.053594	0.222855	0.09934
91	0.50901	0.0657735	-0.261648	-0.065200	0.137782	0.18557
92	-1.26117	-0.8933876	-0.636700	1.247245	0.144735	4.31537
93	0.43575	-0.0824539	-0.249228	0.108551	0.230843	-0.17161
94	0.45444	-0.1756364	-0.110430	0.213353	0.312621	-0.43405
95	0.50156	0.1092184	-0.102169	0.151159	0.214049	0.24251
96	0.56665	0.1458546	-0.156077	0.243511	0.089366	0.14777
97	0.51600	0.0238185	-0.150118	0.384911	0.412624	-0.27365
98	0.13079	-0.0107269	0.258971	-0.638503	0.183203	-0.78337
99	-1.15520	-0.2217551	-0.454159	-0.440926	1.159461	-0.50050
100	0.16212	-0.6471741	1.116638	-0.038458	1.132689	-2.18823
101	-0.24001	0.0192279	-0.226493	-0.264807	1.518874	-0.60627
102	-0.11599	0.1976716	-0.515280	-1.003370	0.138571	1.11994
103	0.64733	0.3052663	-0.419256	1.276644	0.578234	1.57924
104	-1.11702	-2.3690248	0.514637	1.296978	0.465659	-2.84742
105	-0.10665	-1.1266002	0.447219	0.746692	-2.579309	0.71067
106	0.44759	-0.2370311	0.145458	0.248361	-0.631609	-0.01964
107	-1.23431	-4.0505590	2.171436	1.125326	-6.302868	-4.38354
108	-1.49498	-6.1066564	3.697089	1.652859	-10.206392	-6.26252
109	-0.34106	-1.4199603	0.407195	1.536409	-6.151286	2.96967
110	0.17879	-0.1376880	0.745409	-0.005276	0.313371	-0.76639
111	0.20321	-0.1388268	0.526897	-0.068593	0.073984	-1.13060
112	0.31372	0.8027538	0.271162	0.126188	-1.183322	-1.61848
113	0.24603	-0.0397077	0.464399	-0.361817	0.424209	-0.13390
114	0.22132	1.4037175	0.481824	0.275406	-1.960767	-1.50718
115	0.26342	0.7153308	-0.087112	-0.314433	-1.528904	-1.66483

Site constraints (linear combinations of constraining variables)

	RDA1	RDA2	RDA3	RDA4	RDA5	RDA6
2	-0.83261	-0.550308	-0.989115	-0.75342	-0.10901	0.5610350
3	0.02101	-0.399455	-0.751743	-0.86826	-0.05692	0.4685281
4	0.32854	-0.273511	-0.609396	-1.06235	-0.29656	0.4432796
5	0.30214	-0.255889	-0.587299	-0.87542	-0.06074	0.5866228
6	0.39139	-0.152451	-0.614615	-0.98500	-0.15785	0.5605195
7	0.30845	-0.066837	0.029221	-1.36649	-0.29821	0.9039036
8	0.21651	-0.191192	0.305861	-0.22569	1.19550	-0.6118180
9	0.38141	0.386306	-0.009482	-0.35618	0.45255	-0.5207740
10	0.50005	0.050279	0.234626	-0.53860	0.78564	-0.7446360

11	0.31452	0.119027	0.181923	-0.29182	0.86834	-0.5296887
12	0.41368	0.515138	-0.058045	-0.39652	0.33613	-0.4865615
13	0.33722	0.280326	0.016404	-0.54301	0.40717	-0.6660617
14	-0.12427	0.350391	-0.354361	0.19466	-0.56642	-0.0631694
15	-0.62311	0.112652	-0.466564	0.18601	-0.47658	-0.1431333
16	0.19713	0.151218	-0.089701	-0.09497	-0.36165	-0.2972597
17	-0.41055	0.315436	-0.423551	0.13236	-0.48409	-0.0550084
18	-0.24238	0.219112	-0.354373	0.21972	-0.60385	-0.1160145
19	0.41666	0.133341	0.037906	-0.10824	-0.25996	-0.3095431
20	0.62027	0.114277	-0.266776	1.17286	0.83651	1.2647976
21	0.73139	0.346306	-0.393341	1.12137	0.45392	1.2799440
22	0.61591	0.286714	-0.457952	0.95514	0.29207	1.1417328
23	0.64940	0.346428	-0.411209	1.16481	0.51412	1.3235834
24	0.69499	0.403233	-0.445482	1.11734	0.36664	1.2981250
25	0.60510	0.284240	-0.453191	0.95248	0.29536	1.1459299
26	0.69511	0.208921	-0.300599	1.17837	0.70597	1.2860618
27	0.70757	0.296422	-0.369543	1.11853	0.52694	1.2671090
28	0.66942	0.304528	-0.440934	0.94869	0.28956	1.1442370
29	0.29602	0.210902	-0.518715	1.22409	0.52879	1.3036076
30	0.63521	0.308303	-0.418169	1.13049	0.47133	1.2739915
31	0.62421	0.291611	-0.431585	0.94684	0.29398	1.1564752
32	0.15117	-0.338227	0.225999	0.20744	0.50942	-0.2783803
33	-0.91620	0.122771	-0.607452	0.18288	-0.55653	-0.1325991
34	0.43418	0.032639	0.052700	-0.03077	-0.22594	-0.3570535
35	-0.97032	-0.976686	0.118128	0.29069	1.02943	-0.3777168
36	-0.17494	-0.154991	-0.061508	0.14348	0.10991	-0.2528463
37	0.12998	-0.009076	-0.021219	-0.07793	-0.16564	-0.3448287
38	-0.14518	0.057927	-0.163392	0.17591	-0.09034	-0.1773848
39	0.13958	0.258503	-0.176341	0.10013	-0.34165	-0.1812332
40	0.20336	0.150392	-0.116451	-0.06688	-0.38779	-0.3367882
41	0.13350	-0.321836	0.238426	0.17666	0.53720	-0.2858034
42	0.38135	0.300508	-0.055341	0.05645	-0.27477	-0.1740801
43	0.33112	0.030854	-0.083743	-0.02405	-0.22304	-0.4307168
44	-0.78954	0.290819	4.188836	-1.15905	1.06841	2.0185838
45	0.60297	1.330085	0.048368	-0.48279	-0.24272	-0.2008126
46	0.85322	-0.408605	-1.423056	0.77418	0.65371	-2.0525318
47	0.29873	0.617355	0.784804	-0.13602	0.14216	0.0171667
48	0.79943	0.846723	0.777427	-0.35038	0.18154	-0.0908704
49	1.71183	0.754155	0.922395	-0.68164	0.95691	-0.5288648
50	0.22378	-0.993351	0.749343	0.68575	1.31986	-0.7686770
51	0.93499	-0.017770	0.615085	0.31423	0.50430	-0.5572508
52	0.56659	0.017038	0.347880	0.24812	0.11179	-0.6241607
53	0.74070	-0.134322	0.635320	0.31949	0.77648	-0.5467624
54	0.79026	0.175893	0.467193	0.26543	0.25438	-0.4817329
55	0.64668	-0.138310	0.476767	0.25999	0.34496	-0.6753829
56	-2.95141	-1.084319	-0.358827	0.43163	0.85983	0.5751489
57	-0.91295	-0.129694	0.144535	0.01918	0.48099	0.7018469
58	-0.71671	-0.195977	0.269995	-0.23390	0.55429	0.5400687
59	-1.03034	-1.042519	0.657226	0.26547	1.53678	0.5254947
60	-0.13379	-0.852308	-2.928372	1.09105	1.02027	-1.7081738
61	-0.45456	0.642116	0.645993	-0.86950	0.28589	1.0581917
62	-0.69785	-0.445994	-1.148655	-1.16327	-0.07353	0.6919993

63	-0.34250	-0.554208	-0.892732	-1.27016	0.20018	0.6057590
64	0.17020	-0.536129	-0.709250	-1.44794	0.09873	0.4581159
65	-0.49412	-0.431976	-1.034942	-1.21573	0.04431	0.6845249
66	0.12075	-0.395056	-0.785356	-1.26708	0.07415	0.6267966
67	0.20986	-0.445514	-0.737110	-1.47541	0.01996	0.4806034
68	-0.36648	-0.030635	-0.039626	-0.47810	1.34359	-0.7841904
69	-0.07343	0.134533	-0.017433	-0.53314	1.10636	-0.7856588
70	0.03237	0.375706	-0.163566	-0.77831	0.69263	-0.8693753
71	-0.10075	0.088509	-0.019662	-0.44524	1.17984	-0.7677888
72	-0.02202	0.190813	-0.004945	-0.56493	1.08576	-0.7671914
73	0.15595	0.518603	-0.165667	-0.83024	0.59525	-0.8314262
74	-1.60117	1.540433	0.139703	0.42504	0.36519	-0.2671616
75	-1.40550	2.108707	-0.189493	0.39636	-0.53311	-0.1647862
76	-1.34560	1.947283	-0.104842	0.22989	-0.49901	-0.3408396
77	-1.58701	1.694126	0.013466	0.43588	0.12964	-0.2404503
78	-1.66017	2.010324	-0.209650	0.35903	-0.39197	-0.1719997
79	-1.42977	2.029067	-0.199916	0.22086	-0.62471	-0.3178006
80	-0.18841	-0.063952	0.600233	-0.64978	0.36298	0.0000771
81	0.04714	-0.158333	0.346014	0.02053	-0.70519	-0.0779258
82	0.51536	-0.100853	0.516174	-0.15249	-0.89587	-0.1883925
83	-0.79550	-0.338330	0.542553	-0.61840	0.54834	-0.0071493
84	0.08095	-0.118818	0.575367	-0.29427	-0.25146	-0.0287243
85	0.59682	-0.170437	0.627503	-0.19120	-0.70917	-0.2088539
86	-0.28823	-0.809345	0.244318	0.07583	-0.26472	-0.1375332
87	0.39064	-0.158575	0.242920	-0.15733	-0.83817	-0.0092950
88	0.32291	-0.276338	0.211345	-0.32082	-0.89768	-0.1836131
89	-0.25292	-0.615108	0.128935	0.08036	-0.55261	-0.0844586
90	0.30423	-0.119741	0.159615	-0.15302	-0.92009	-0.0036230
91	0.29695	-0.248015	0.189993	-0.32351	-0.94441	-0.1690253
92	-0.19035	-0.440876	0.179109	-0.02550	-0.69301	0.1198724
93	0.33558	-0.028725	0.128762	-0.07343	-1.24753	0.1681152
94	0.33331	-0.110911	0.129065	-0.25585	-1.31669	0.0098914
95	0.32757	-0.140908	0.200123	-0.02056	-1.01267	0.1667360
96	0.32742	-0.017434	0.093337	-0.05385	-1.29482	0.1632234
97	0.30484	-0.226741	0.166650	-0.20457	-1.23756	-0.0194407
98	-0.78913	-0.892997	0.869968	1.27064	-0.02260	-0.8611579
99	-0.28557	-0.832598	1.107316	1.19720	-0.03235	-0.8786772
100	0.21490	-0.848638	1.271315	1.04838	-0.12784	-1.0484473
101	-0.82441	-0.618083	0.666139	1.28438	-0.46462	-0.7795240
102	0.28440	0.397772	0.599216	1.00530	-1.38222	-0.6020168
103	0.31971	0.433433	0.451449	0.85741	-1.65340	-0.7730832
104	-1.18160	-1.705156	-0.051755	0.52879	-0.65042	0.2429411
105	0.16100	-0.904498	0.181005	0.21054	-1.13210	0.3644198
106	0.33234	-1.041152	0.334779	0.11161	-0.99107	0.2518334
107	-1.12068	-1.973167	0.139706	0.56640	-0.30065	0.1681983
108	-0.66261	-1.413520	0.094883	0.32257	-0.77360	0.2770920
109	0.27991	-0.765926	0.240999	-0.07293	-1.10989	0.3379293
110	0.04004	-0.098284	-0.059489	-0.40661	0.67055	-0.2054994
111	0.05647	0.461231	-0.468344	-0.43543	-0.25862	-0.0978983
112	0.17287	0.326373	-0.365118	-0.59789	-0.26049	-0.2612994
113	-0.04653	0.395118	-0.452554	-0.38191	-0.12343	-0.0685557
114	0.38139	0.277652	-0.312720	-0.39800	0.06439	-0.2294549

115 0.31860 0.172032 -0.220045 -0.58315 -0.01335 -0.3234757

Biplot scores for constraining variables

	RDA1	RDA2	RDA3	RDA4	RDA5	RDA6
R.Depth	0.29747	0.16133	-0.03003	-0.15248	-0.24717	-0.09637
\`Site age\`	-0.28668	-0.27987	-0.38965	-0.15011	-0.08449	0.581389
\`Ratio %\`	-0.22734	0.32142	0.06929	-0.18816	0.07126	-0.29277
Com	-0.64404	0.36654	-0.08966	0.11719	0.13466	0.008016
Ind	0.31906	0.14387	-0.20754	0.55108	0.23577	0.629514
\`Fuel st.\`	-0.02368	-0.4217	0.32972	0.14773	-0.634	-0.11172
\`% OM\`	-0.64842	-0.27823	0.24619	0.01182	0.22774	-0.08407
\`% Sand\`	0.3905	0.36583	-0.49025	-0.0286	-0.39018	0.210111
\`% Silt\`	-0.41311	-0.39124	0.49478	0.04687	0.40192	-0.21609
\`% Clay\`	-0.13861	-0.09703	0.35914	-0.11866	0.22266	-0.12251

References

(1) Mathews, J. Rainwater and Land Development - Ohio's Standards for Stormwater Management Land Development and Urban Stream Protection (Third Edition), 2006. https://crwp.org/wp-content/uploads/2020/09/ODNR-Rainwater-and-Land-Development-Manual.pdf (accessed 2024-10-07).

(2) DIN 38414-14. *Standard - German standard methods for the examination of water, waste water and sludge - Sludge and sediments (group S) - Part 14: Determination of selected polyfluorinated compounds (PFC) in sludge, compost and soil - Method using high performance liquid chromatography and mass spectrometric detection (HPLC-MS/MS) (S 14) DIN 38414-14 - Swedish Institute for Standards, SIS*. Svenska institutet för standarder, SIS. https://www.sis.se/en/produkter/environment-health-protection-safety/water-quality/examination-of-water-for-chemical-substances/din3841414/ (accessed 2024-05-17).

(3) Powley, C. R.; George, S. W.; Ryan, T. W.; Buck, R. C. Matrix Effect-Free Analytical Methods for Determination of Perfluorinated Carboxylic Acids in Environmental Matrixes. *Anal Chem* **2005**, *77* (19), 6353–6358. https://doi.org/10.1021/ac0508090.

(4) Houtz, E. F.; Sedlak, D. L. Oxidative Conversion as a Means of Detecting Precursors to Perfluoroalkyl Acids in Urban Runoff. *Environ. Sci. Technol.* **2012**, *46* (17), 9342–9349. https://doi.org/10.1021/es302274g.

(5) CSN EN 12879. *ČSN EN 12879 (758005)- Charakterizace kalů - Stanovení ztráty žíháním (Sludge characterization - determination of loss by annealing*. https://www.technicke-normy-csn.cz/csn-en-12879-758005-226751.html# (accessed 2024-05-17).

(6) CSN 72 0103. *ČSN 72 0103 (720103) Základní postup rozboru silikátů - Stanovení ztráty žíháním (Basic procedure for the analysis of silicates - determination of loss by annealing)*. https://www.technicke-normy-csn.cz/csn-72-0103-720103-218183.html# (accessed 2024-05-17).

(7) CSN 46 5735. *ČSN 46 5735 (465735) Průmyslové komposty (Industrial composts)*. https://www.technicke-normy-csn.cz/csn-46-5735-465735-206692.html# (accessed 2024-05-17).

(8) ASTM D2974-20. *Standard Test Methods for Determining the Water (Moisture) Content, Ash Content, and Organic Material of Peat and Other Organic Soils*. https://www.scribd.com/document/585909377/ASTM-D-2974-2020 (accessed 2024-05-17).

(9) Faé, G. S.; Montes, F.; Bazilevskaya, E.; Añó, R. M.; Kemanian, A. R. Making Soil Particle Size Analysis by Laser Diffraction Compatible with Standard Soil Texture Determination Methods. *Soil Science Society of America Journal* **2019**, *83* (4), 1244–1252. https://doi.org/10.2136/sssaj2018.10.0385.

(10) Yang, X. M.; Drury, C. F.; Reynolds, W. D.; MacTavish, D. C. Use of Sonication to Determine the Size Distributions of Soil Particles and Organic Matter. *Can. J. Soil. Sci.* **2009**, *89* (4), 413–419. https://doi.org/10.4141/cjss08063.

(11) DIN ISO 9277. *DIN ISO 9277 - Determination of the specific surface area of solids by gas adsorption - BET method (ISO 9277:2010)*. https://www.en-standard.eu/din-iso-9277-determination-of-the-specific-surface-area-of-solids-by-gas-adsorption-bet-method-iso-9277-2010/ (accessed 2024-05-17).

(12) SS-EN 12880. *Standard - Characterization of sludges - Determination of dry residue and water content SS-EN 12880 - Swedish Institute for Standards, SIS*. Svenska institutet för standarder, SIS. https://www.sis.se/en/produkter/environment-health-protection-safety/wastes/liquid-wastes-sludge/ssen12880/ (accessed 2024-05-17).

(13) SS-EN ISO 10523. *Standard - Water quality - Determination of pH ISO 10523:2008 - Swedish Institute for Standards, SIS*. Svenska institutet för standarder, SIS.

https://www.sis.se/en/produkter/environment-health-protection-safety/water-quality/examination-of-water-for-chemical-substances/iso105232008/ (accessed 2024-05-17).
(14) Nestingen, R.; Asleson, B. C.; Gulliver, J. S.; Hozalski, R. M.; Nieber, J. L. Laboratory Comparison of Field Infiltrometers. *Journal of Sustainable Water in the Built Environment* **2018**, *4* (3), 04018005. https://doi.org/10.1061/JSWBAY.0000857.
(15) Ahmed, F.; Nestingen, R.; Nieber, J. l.; Gulliver, J. s.; Hozalski, R. m. A Modified Philip–Dunne Infiltrometer for Measuring the Field-Saturated Hydraulic Conductivity of Surface Soil. *Vadose Zone Journal* **2014**, *13* (10), vzj2014.01.0012. https://doi.org/10.2136/vzj2014.01.0012.
(16) Higgins, C. P.; Luthy, R. G. Sorption of Perfluorinated Surfactants on Sediments. *Environ. Sci. Technol.* **2006**, *40* (23), 7251–7256. https://doi.org/10.1021/es061000n.
(17) Ahrens, L.; Yeung, L. W. Y.; Taniyasu, S.; Lam, P. K. S.; Yamashita, N. Partitioning of Perfluorooctanoate (PFOA), Perfluorooctane Sulfonate (PFOS) and Perfluorooctane Sulfonamide (PFOSA) between Water and Sediment. *Chemosphere* **2011**, *85* (5), 731–737. https://doi.org/10.1016/j.chemosphere.2011.06.046.
(18) Ahrens, L.; Gashaw, H.; Sjöholm, M.; Gebrehiwot, S. G.; Getahun, A.; Derbe, E.; Bishop, K.; Åkerblom, S. Poly- and Perfluoroalkylated Substances (PFASs) in Water, Sediment and Fish Muscle Tissue from Lake Tana, Ethiopia and Implications for Human Exposure. *Chemosphere* **2016**, *165*, 352–357. https://doi.org/10.1016/j.chemosphere.2016.09.007.
(19) Dalahmeh, S.; Tirgani, S.; Komakech, A. J.; Niwagaba, C. B.; Ahrens, L. Per- and Polyfluoroalkyl Substances (PFASs) in Water, Soil and Plants in Wetlands and Agricultural Areas in Kampala, Uganda. *Science of The Total Environment* **2018**, *631–632*, 660–667. https://doi.org/10.1016/j.scitotenv.2018.03.024.
(20) Aly, Y. H.; Liu, C.; McInnis, D. P.; Lyon, B. A.; Hatton, J.; McCarty, M.; Arnold, W. A.; Pennell, K. D.; Simcik, M. F. In Situ Remediation Method for Enhanced Sorption of Perfluoro-Alkyl Substances onto Ottawa Sand. *Journal of Environmental Engineering* **2018**, *144* (9), 04018086. https://doi.org/10.1061/(ASCE)EE.1943-7870.0001418.
(21) Barzen-Hanson, K. A.; Davis, S. E.; Kleber, M.; Field, J. A. Sorption of Fluorotelomer Sulfonates, Fluorotelomer Sulfonamido Betaines, and a Fluorotelomer Sulfonamido Amine in National Foam Aqueous Film-Forming Foam to Soil. *Environ. Sci. Technol.* **2017**, *51* (21), 12394–12404. https://doi.org/10.1021/acs.est.7b03452.
(22) Ruyle, B. J.; Thackray, C. P.; Butt, C. M.; LeBlanc, D. R.; Tokranov, A. K.; Vecitis, C. D.; Sunderland, E. M. Centurial Persistence of Forever Chemicals at Military Fire Training Sites. *Environ. Sci. Technol.* **2023**, *57* (21), 8096–8106. https://doi.org/10.1021/acs.est.3c00675.
(23) Li, Y.; Oliver, D. P.; Kookana, R. S. A Critical Analysis of Published Data to Discern the Role of Soil and Sediment Properties in Determining Sorption of per and Polyfluoroalkyl Substances (PFASs). *Science of The Total Environment* **2018**, *628–629*, 110–120. https://doi.org/10.1016/j.scitotenv.2018.01.167.
(24) Flanagan, K.; Blecken, G.-T.; Österlund, H.; Nordqvist, K.; Viklander, M. Contamination of Urban Stormwater Pond Sediments: A Study of 259 Legacy and Contemporary Organic Substances. *Environ. Sci. Technol.* **2021**, *55* (5), 3009–3020. https://doi.org/10.1021/acs.est.0c07782.
(25) Crane, J. L. Distribution, Toxic Potential, and Influence of Land Use on Conventional and Emerging Contaminants in Urban Stormwater Pond Sediments. *Arch Environ Contam Toxicol* **2019**, *76* (2), 265–294. https://doi.org/10.1007/s00244-019-00598-w.
(26) Wei, H.; Flanagan, K.; Lundy, L.; Muthanna, T. M.; Viklander, M. A Study of 101 Organic Substances in Gully Pot Sediments Accumulated over a One-Year Period in Stockholm, Sweden. *Science of The Total Environment* **2023**, *894*, 165028. https://doi.org/10.1016/j.scitotenv.2023.165028.
(27) Xiao, F.; Simcik, M. F.; Gulliver, J. S. Perfluoroalkyl Acids in Urban Stormwater Runoff: Influence of Land Use. *Water Research* **2012**, *46* (20), 6601–6608. https://doi.org/10.1016/j.watres.2011.11.029.
(28) Gao, Y.; Liang, Y.; Gao, K.; Wang, Y.; Wang, C.; Fu, J.; Wang, Y.; Jiang, G.; Jiang, Y. Levels, Spatial Distribution and Isomer Profiles of Perfluoroalkyl Acids in Soil, Groundwater and Tap Water

around a Manufactory in China. *Chemosphere* **2019**, *227*, 305–314. https://doi.org/10.1016/j.chemosphere.2019.04.027.
(29) Wang, Y.; Fu, J.; Wang, T.; Liang, Y.; Pan, Y.; Cai, Y.; Jiang, G. Distribution of Perfluorooctane Sulfonate and Other Perfluorochemicals in the Ambient Environment around a Manufacturing Facility in China. *Environ. Sci. Technol.* **2010**, *44* (21), 8062–8067. https://doi.org/10.1021/es101810h.
(30) Ahmadireskety, A.; Da Silva, B. F.; Robey, N. M.; Douglas, T. E.; Aufmuth, J.; Solo-Gabriele, H. M.; Yost, R. A.; Townsend, T. G.; Bowden, J. A. Per- and Polyfluoroalkyl Substances (PFAS) in Street Sweepings. *Environ. Sci. Technol.* **2022**, *56* (10), 6069–6077. https://doi.org/10.1021/acs.est.1c03766.
(31) Codling, G.; Vogt, A.; Jones, P. D.; Wang, T.; Wang, P.; Lu, Y.-L.; Corcoran, M.; Bonina, S.; Li, A.; Sturchio, N. C.; Rockne, K. J.; Ji, K.; Khim, J.-S.; Naile, J. E.; Giesy, J. P. Historical Trends of Inorganic and Organic Fluorine in Sediments of Lake Michigan. *Chemosphere* **2014**, *114*, 203–209. https://doi.org/10.1016/j.chemosphere.2014.03.080.
(32) Codling, G.; Hosseini, S.; Corcoran, M. B.; Bonina, S.; Lin, T.; Li, A.; Sturchio, N. C.; Rockne, K. J.; Ji, K.; Peng, H.; Giesy, J. P. Current and Historical Concentrations of Poly and Perfluorinated Compounds in Sediments of the Northern Great Lakes – Superior, Huron, and Michigan. *Environmental Pollution* **2018**, *236*, 373–381. https://doi.org/10.1016/j.envpol.2018.01.065.
(33) Codling, G.; Sturchio, N. C.; Rockne, K. J.; Li, A.; Peng, H.; Tse, T. J.; Jones, P. D.; Giesy, J. P. Spatial and Temporal Trends in Poly- and per-Fluorinated Compounds in the Laurentian Great Lakes Erie, Ontario and St. Clair. *Environmental Pollution* **2018**, *237*, 396–405. https://doi.org/10.1016/j.envpol.2018.02.013.
(34) Rankin, K.; Mabury, S. A.; Jenkins, T. M.; Washington, J. W. A North American and Global Survey of Perfluoroalkyl Substances in Surface Soils: Distribution Patterns and Mode of Occurrence. *Chemosphere* **2016**, *161*, 333–341. https://doi.org/10.1016/j.chemosphere.2016.06.109.
(35) Gaspéri, J.; Ayrault, S.; Moreau-Guigon, E.; Alliot, F.; Labadie, P.; Budzinski, H.; Blanchard, M.; Muresan, B.; Caupos, E.; Cladière, M.; Gateuille, D.; Tassin, B.; Bordier, L.; Teil, M.-J.; Bourges, C.; Desportes, A.; Chevreuil, M.; Moilleron, R. Contamination of Soils by Metals and Organic Micropollutants: Case Study of the Parisian Conurbation. *Environ Sci Pollut Res* **2018**, *25* (24), 23559–23573. https://doi.org/10.1007/s11356-016-8005-2.
(36) Zhu, H.; Kannan, K. Distribution and Partitioning of Perfluoroalkyl Carboxylic Acids in Surface Soil, Plants, and Earthworms at a Contaminated Site. *Science of The Total Environment* **2019**, *647*, 954–961. https://doi.org/10.1016/j.scitotenv.2018.08.051.
(37) Washington, J. W.; Henderson, W. M.; Ellington, J. J.; Jenkins, T. M.; Evans, J. J. Analysis of Perfluorinated Carboxylic Acids in Soils II: Optimization of Chromatography and Extraction. *Journal of Chromatography A* **2008**, *1181* (1), 21–32. https://doi.org/10.1016/j.chroma.2007.12.042.
(38) Li, F.; Zhang, C.; Qu, Y.; Chen, J.; Chen, L.; Liu, Y.; Zhou, Q. Quantitative Characterization of Short- and Long-Chain Perfluorinated Acids in Solid Matrices in Shanghai, China. *Science of The Total Environment* **2010**, *408* (3), 617–623. https://doi.org/10.1016/j.scitotenv.2009.10.032.
(39) Naile, J. E.; Khim, J. S.; Hong, S.; Park, J.; Kwon, B.-O.; Ryu, J. S.; Hwang, J. H.; Jones, P. D.; Giesy, J. P. Distributions and Bioconcentration Characteristics of Perfluorinated Compounds in Environmental Samples Collected from the West Coast of Korea. *Chemosphere* **2013**, *90* (2), 387–394. https://doi.org/10.1016/j.chemosphere.2012.07.033.
